# Neoadjuvant nivolumab with or without relatlimab in resectable non-small-cell lung cancer: a randomized phase 2 trial

**DOI:** 10.1038/s41591-024-02965-0

**Published:** 2024-04-30

**Authors:** Martin Schuler, Kristof Cuppens, Till Plönes, Marcel Wiesweg, Bert Du Pont, Balazs Hegedus, Johannes Köster, Fabian Mairinger, Kaid Darwiche, Annette Paschen, Brigitte Maes, Michel Vanbockrijck, David Lähnemann, Fang Zhao, Hubertus Hautzel, Dirk Theegarten, Koen Hartemink, Henning Reis, Paul Baas, Alexander Schramm, Clemens Aigner

**Affiliations:** 1 West German Cancer Center, Department of Medical Oncology, University Hospital Essen, Essen, Germany; 2 Medical Faculty, University Duisburg-Essen, Essen, Germany; 3 National Center for Tumor Diseases (NCT) West, Essen, Germany; 4 Department of Pulmonology and Thoracic Oncology, and Jessa and Science, Jessa Hospital, Hasselt, Belgium; 5 Faculty of Medicine and Life Sciences LCRC, UHasselt, Diepenbeek, Belgium; 6 West German Cancer Center, Department of Thoracic Surgery, University Medicine Essen – Ruhrlandklinik, Essen, Germany; 7 Department of Thoracic and Vascular Surgery, Jessa Hospital, Hasselt, Belgium; 8 Bioinformatics and Computational Oncology, Institute for Artificial Intelligence in Medicine, University Hospital Essen, Essen, Germany; 9 West German Cancer Center, Institute for Pathology, University Hospital Essen, Essen, Germany; 10 West German Cancer Center, Department of Pulmonary Medicine, University Medicine Essen – Ruhrlandklinik, Essen, Germany; 11 West German Cancer Center, Department of Dermatology, University Hospital Essen, Essen, Germany; 12 Laboratory Medicine Department, Laboratory for Molecular Diagnostics, Jessa Hospital, Hasselt, Belgium; 13 Department of Pathology, Jessa Hospital, Hasselt, Belgium; 14 West German Cancer Center, Department of Nuclear Medicine, University Hospital Essen, Essen, Germany; 15 Department of Surgery, Netherlands Cancer Institute, Antoni van Leeuwenhoek Hospital, Amsterdam, The Netherlands; 16 University Hospital Frankfurt, Dr Senckenberg Institute of Pathology, Goethe University, Frankfurt, Germany; 17 Department of Thoracic Oncology, Netherlands Cancer Institute, Antoni van Leeuwenhoek Hospital, Amsterdam, The Netherlands; 18 Present Address: University Hospital Carl Gustav Carus, Department of Surgery, Division of Thoracic Surgery, Technical University Dresden, Dresden, Germany; 19 Present Address: General Hospital Vienna, Department of Thoracic Surgery, Medical University Vienna, Vienna, Austria

**Keywords:** Translational research, Drug development, Randomized controlled trials

## Abstract

Antibodies targeting the immune checkpoint molecules PD-1, PD-L1 and CTLA-4, administered alone or in combination with chemotherapy, are the standard of care in most patients with metastatic non-small-cell lung cancers. When given before curative surgery, tumor responses and improved event-free survival are achieved. New antibody combinations may be more efficacious and tolerable. In an ongoing, open-label phase 2 study, 60 biomarker-unselected, treatment-naive patients with resectable non-small-cell lung cancer were randomized to receive two preoperative doses of nivolumab (anti-PD-1) with or without relatlimab (anti-LAG-3) antibody therapy. The primary study endpoint was the feasibility of surgery within 43 days, which was met by all patients. Curative resection was achieved in 95% of patients. Secondary endpoints included pathological and radiographic response rates, pathologically complete resection rates, disease-free and overall survival rates, and safety. Major pathological (≤10% viable tumor cells) and objective radiographic responses were achieved in 27% and 10% (nivolumab) and in 30% and 27% (nivolumab and relatlimab) of patients, respectively. In 100% (nivolumab) and 90% (nivolumab and relatlimab) of patients, tumors and lymph nodes were pathologically completely resected. With 12 months median duration of follow-up, disease-free survival and overall survival rates at 12 months were 89% and 93% (nivolumab), and 93% and 100% (nivolumab and relatlimab). Both treatments were safe with grade ≥3 treatment-emergent adverse events reported in 10% and 13% of patients per study arm. Exploratory analyses provided insights into biological processes triggered by preoperative immunotherapy. This study establishes the feasibility and safety of dual targeting of PD-1 and LAG-3 before lung cancer surgery.

ClinicalTrials.gov Indentifier: NCT04205552.

## Abstract (web summary)

In an open-label phase 2 trial, patients with non-small-cell lung cancer received neoadjuvant anti-PD-1 with or without anti-LAG-3, showing that curative intent surgery after combined blockade of PD-1 and LAG-3 is feasible, and leads to preliminary clinical responses.

## Main

Lung cancer is the leading cancer fatality on a global scale, with the number of deaths surpassing those of breast, colorectal and prostate cancer combined^1^. Despite advances in early detection, the majority of patients are still diagnosed with advanced stage disease. The introduction of precision therapies targeting specific oncogenic mutations in lung adenocarcinomas (LUAD), and monoclonal antibodies modulating the PD-1/PD-L1 and CTLA-4 immune checkpoints in non-small-cell lung cancers (NSCLC) and small-cell lung cancers have significantly improved treatment outcomes in metastatic disease^2,3^. More recently, these paradigms have been successfully translated to treatment algorithms for localized NSCLC that are based on curative surgery. This includes adjuvant osimertinib following resection of *EGFR*-mutated NSCLC^4^, alectinib in resected ALK-positive NSCLC^5^ and atezolizumab or pembrolizumab following resection and adjuvant chemotherapy in NSCLC^6,7^.

Clinical and biological considerations provide strong arguments for moving relapse-preventing systemic therapies to the preoperative or perioperative setting. First, preoperative treatment is not delayed or prevented by postoperative morbidity and protracted recovery from surgery. Second, the response to risk-reducing cancer medicines can be monitored by imaging and histopathology of the primary tumor. Specifically in the setting of immune checkpoint inhibitor (ICI) therapy, reinvigoration of a suppressed immune response may be more effective while tumor-infiltrating lymphocytes are still present in their native tumor context. Clinical proof-of-concept has been provided by the SWOG S1801 study in patients with resectable melanoma, which demonstrated improved disease-free survival (DFS) by moving 3 of 18 doses of pembrolizumab to the preoperative window^8^.

Several studies have piloted preoperative ICI therapy directed against PD-1, PD-L1, CTLA-4 and less-established targets in patients with resectable NSCLC^9–13^. Next to demonstrating safety and feasibility, the spectra of clinical and histopathological responses observed in these studies were correlated with exploratory biomarker analyses. More recently, preoperative PD-1/PD-L1 antibodies combined with platinum-based chemotherapy have been explored^14–18^. Although this approach resulted in impressive histopathological response rates and improved event-free survival, combined chemoimmunotherapy may obscure the contribution of the ICI component at the single patient level. Across larger studies of preoperative chemoimmunotherapy approximately 20% of patients failed to proceed to curatively intended surgery. Further, patients who might have been served perfectly well with ICI therapy alone were exposed to the additional toxicities of chemotherapy.

Studies combining two ICIs in unselected patient populations with metastatic NSCLC have so far produced similar outcomes to therapies targeting PD-1/PD-L1 alone or combined with chemotherapy^19–22^. Nevertheless, it is conceivable that simultaneous blockade of more than one immune checkpoint can extend clinical activity to yet undefined patient populations or prolong duration of disease control.

Based on their distinct and potentially synergistic mode of action, combined targeting of the immune checkpoints LAG-3 and PD-1 is a rational choice to overcome immune resistance in NSCLC. Both PD-1 and LAG-3 are expressed by exhausted T cells. Dual blockade of both immune checkpoints synergistically enhanced T cell function and antitumor activity in preclinical models^23,24^. Importantly, in a randomized phase 3 study in patients with unresectable or metastatic melanoma combining the PD-1 antibody nivolumab and relatlimab, an immunoglobulin G4 antibody blocking LAG-3 was superior to nivolumab monotherapy in terms of radiographic response and progression-free survival endpoints^25^. Combination therapy was safe despite some increase in treatment-related adverse events (AEs), particularly thyroiditis, diarrhea and hepatitis. Myocarditis was reported in 1.7% of patients receiving nivolumab and relatlimab under routine troponin monitoring^25^. This study supported approvals of this novel ICI combination therapy by the U.S. Food and Drug Administration and the European Medicines Agency.

**Fig. 1:**
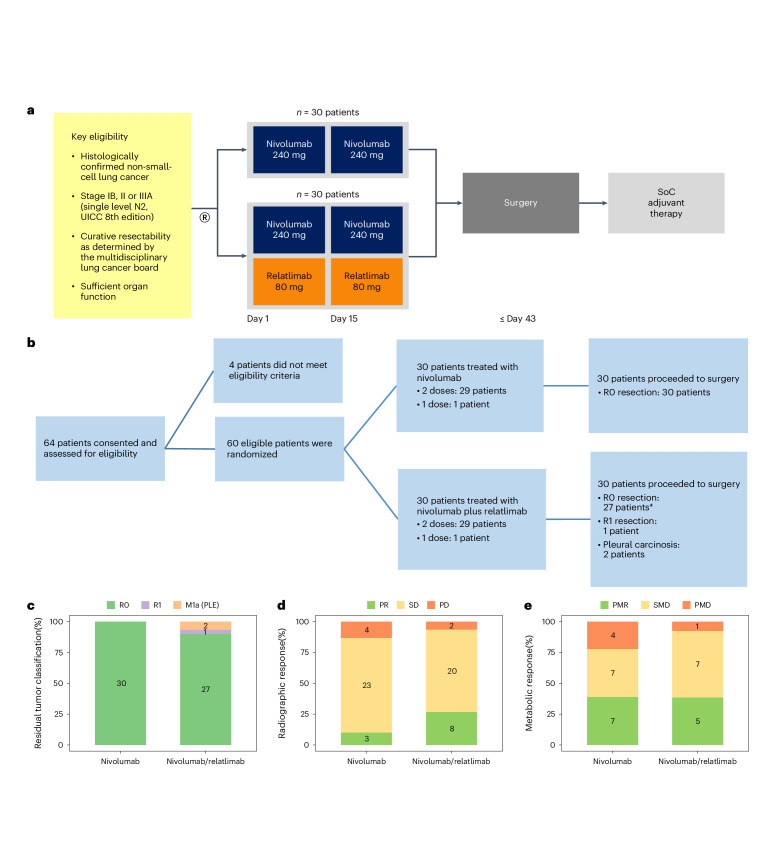
Study design, patient deposition and secondary endpoints. **a**, Graphical representation of clinical study design including key inclusion criteria. **b**, Patient deposition during the phases of the clinical study including screening, preoperative immunotherapy and curative resection. Reasons for screening failure and outcomes of surgery are summarized (*including one patient with single bone metastasis). **c**, Fraction of patients (*n* = 60) with microscopically complete (R0, green), microscopically incomplete (R1, purple) and macroscopically incomplete (pleural carcinosis, M1a (PLE), orange) resection of primary lung cancers and, if present, lymph node metastases per study arm. **d**, Fraction of patients (*n* = 60) with complete (none), partial response (PR, green), stable (SD, yellow) and progressive disease (PD, red) per RECIST evaluation of CT scans per study arm. **e**, Fraction of patients (*n* = 31) with complete (none), partial metabolic response (PMR, green), metabolically stable (SMD, yellow) and metabolically progressive disease (PMD, red) per PERCIST evaluation of positron emission tomography scans per study arm. SoC, standard of care.

Against this background, the study NEOpredict-Lung (NCT04205552) was designed to explore the feasibility and safety of preoperative dual targeting of PD-1 and LAG-3 in patients with resectable NSCLC stages IB, II or IIIA (Fig. 1a and Supplementary information). Secondary endpoints include the assessment of pathological and radiographic responses, survival endpoints and quality of surgical resections. Moreover, the study intends to leverage the neoadjuvant setting for exploratory analyses of specific biologies associated with response or resistance. Patients are randomly assigned to nivolumab plus relatlimab or nivolumab monotherapy, the latter serving as a reference for the evaluation of toxicity, clinical activity and biological impact of dual targeting of PD-1 and LAG-3 in resectable NSCLC.

## Results

### Study design and patient disposition

Between 4 March 2020 and 25 July 2022, 64 patients were screened and 60 patients were enrolled at three study sites. All patients provided written informed consent; study participation was not compensated. Patients were randomized between two preoperative treatments given every 14 days with nivolumab (240 mg, arm A) and nivolumab plus relatlimab (240 and 80 mg, arm B) (Fig. 1a,b). Demographics and patient characteristics are summarized in Table 1. Fifty-eight (97%) patients received the planned two doses of nivolumab or nivolumab plus relatlimab; the second dose of nivolumab or nivolumab plus relatlimab was withheld in one patient each because of immune-related AEs, which fully resolved subsequently. All 60 patients (100%) proceeded to surgery within the protocol-defined time frame. Clinical data are reported as of 16 May 2023.

**Table 1:** Patient demographics and characteristics

	Nivolumab	Nivolumab plus relatlimab
***n*** **(female, male)**	30 (15, 15)	30 (13, 17)
**Age in years, median (range)**	64 (43–77)	67 (43–81)
**ECOG PS (0, 1)**	28, 2	28, 2
**Histology**		
Adenocarcinoma	13	15
Squamous cell carcinoma	10	9
Adenosquamous carcinoma	2	2
Other	5	4
**Clinical stage (UICC eighth** **edition)**		
IB	8	10
IIA	5	1
IIB	13	16
IIIA	4	3
**PD-L1 status (TPS)**		
<1%	6	8
1–49%	14	15
≥50%	10	7
**Smoking status**		
Current	5	16
Former	22	13
Nonsmoker	3	1
**Occupational exposure**		
Yes	2	3
No	27	26
Unknown	1	1

ECOG PS, Eastern Cooperative Oncology Group performance score; TPS, tumor proportional score.

### Primary outcome

The clinical study was designed to confirm the feasibility of two preoperative doses of nivolumab plus relatlimab or nivolumab without delaying curatively intended surgery (Fig. 1a). Based on analyses of surgical registries^26^ a screening period of up to 28 days and a treatment period of up to 42 days were considered safe with respect to surgical survival outcomes. The primary study endpoint was met by all 60 randomized patients, thus confirming feasibility of both arms of preoperative ICI treatment.

### Secondary outcomes

Radiographic responses to immunotherapy were evaluated immediately before surgery per Response Evaluation Criteria In Solid Tumors (RECIST) 1.1. There were no complete radiographic responses; the partial response rates were 10% with nivolumab monotherapy and 27% with nivolumab plus relatlimab (Fig. 1d).

**Fig. 2:**
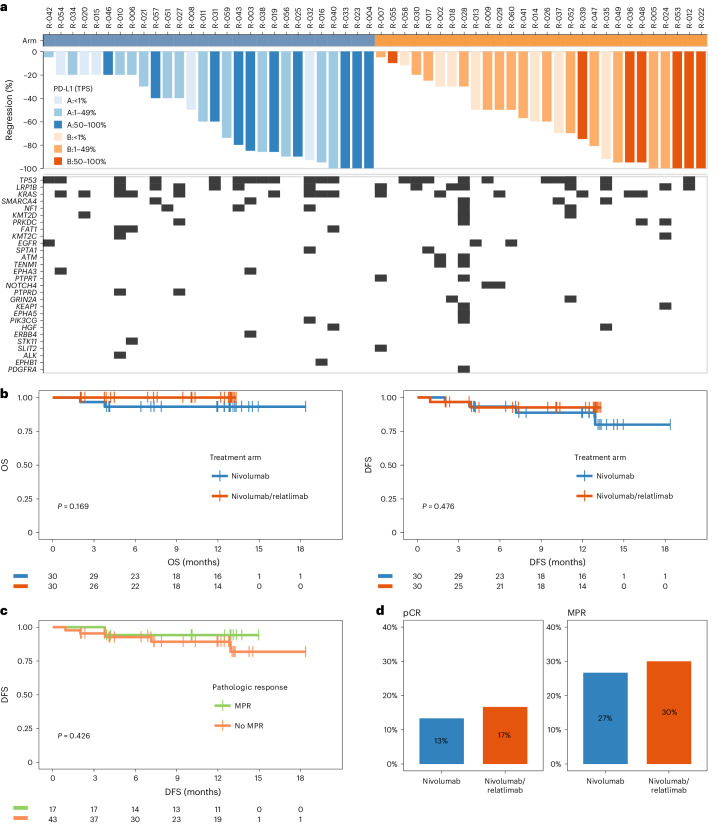
Pathological responses, biomarkers and survival outcomes. **a**, Waterfall plots of pathologic tumor regression (percentage reduction of viable tumor cells) in resected tumors and lymph nodes following neoadjuvant treatment with nivolumab (arm A, blue) or nivolumab and relatlimab (arm B, red). The color intensity encodes the category of PD-L1 expression by tumor cells (TPS <1% light color, TPS 1–49% medium dark color, TPS 50–100% dark color). The lower panel depicts the oncogram of each tumor using next-generation DNA sequencing of 500 cancer-related genes. Boxes represent pathogenic genomic aberrations in the respective gene. Genes with pathogenic aberrations in at least two study patients are listed. **b**, Kaplan–Meier plots for OS (left) and DFS (right) per study arm (arm A nivolumab, blue; arm B nivolumab and relatlimab, red). **c**, Kaplan–Meier plot for DFS in patients achieving a MPR (≤10% viable tumor cells (green)), and not achieving a MPR (>10% viable tumor cells (orange)). Statistical comparisons by log-rank test, vertical lines indicate censored patients. Two patients (both arm A) had died from noncancer causes. Six patients (four in arm A and two in arm B) have recurred or died. No patient with MPR has recurred, one patient with MPR had died from a noncancer cause. **d**, Fraction and number of patients with complete pathological response (pCR, upper) and MPR (lower) in study arms A (nivolumab (blue)) and B (nivolumab and relatlimab (red)).

Pathological response was evaluated in resected tumors and lymph nodes from 59 patients (30 in arm A and 29 in arm B) at each study site following standardized criteria^27^. There were four (13%) complete pathological responses with nivolumab and five (17%) complete pathological responses with nivolumab plus relatlimab (Fig. 2d). The rates of major pathological responses (MPR, ≤10% viable tumor cells) were 27% and 30% (Fig. 2d), pathological responses (≤50% viable tumor cells) were observed in 60% and 72% of resected tumors and lymph nodes, respectively. In both study arms, deeper pathological responses clustered in patients with PD-L1-positive tumors (Fig. 2a).

Complete surgical resection (R0) was achieved in 57 patients (95%) (Fig. 1c). One patient had R1 resection; pleural carcinosis was detected intraoperatively in two patients, which had been undetectable by preoperative imaging studies. In one patient, a single small bone metastasis was detected during perioperative hospitalization. The treatment plan remained curative for oligometastatic disease: primary tumor and lymph nodes were R0 resected, followed by postoperative standard of care systemic therapy and stereotactic radiotherapy (Fig. 1b). Including this patient, 30 patients have received standard of care postoperative chemotherapy (15 per arm), whereas 30 patients underwent no further adjuvant treatment.

With a median duration of follow-up of 12 months, rates of DFS and overall survival (OS) at 12 months were 89% and 93% with nivolumab monotherapy, and 93% and 100% with nivolumab plus relatlimab (Fig. 2b). So far, no patient achieving MPR has relapsed; one patient with MPR died from pulmonary embolism during extended follow-up (Fig. 2c).

### Safety

Of 60 randomized and treated patients, 92% experienced at least one AE during preoperative immunotherapy. The most common AEs included mild to moderate respiratory symptoms, thyroid function abnormalities, gastrointestinal symptoms, fatigue, laboratory abnormalities and musculoskeletal symptoms (Table 2). Serious AEs were observed in 30% (arm A) and 33% (arm B) of patients, respectively. Treatment-emergent AEs were reported in 53% (arm A) and 63% (arm B) of patients (Table 2). The most common immune-related AEs were hyperthyroidism and hypothyroidism. Grade 3 hyperthyroidism was observed in one patient (arm A). Additional immune-related AEs included increased liver enzymes and arthralgia (Table 2). In arm A there were two cases of pneumonitis (grade 1 and 2); likewise there were two cases in arm B (both grade 1).

**Table 2:** Summary of adverse events

	Nivolumab *n* (%)	Nivolumab plus relatlimab *n* (%)
**AE**	27 (90)	28 (93)
Grade ≥ 3	12 (40)	16 (53)
Serious	9 (30)	10 (33)
Led to death	2 (7)	—
Prolonged hospitalization	8 (27)	10 (33)
Important medical event	1 (3)	1 (3)
**Treatment-emergent AE**	16 (53)	19 (63)
Grade ≥ 3	3 (10)	4 (13)
Led to death	1 (3)	—
**Treatment-emergent AE with incidence ≥10% at least in one arm**		
Atrial fibrillation	5 (17)	2 (7)
Hyperthyroidism	7 (23)	7 (23)
Hypothyroidism	3 (10)	5 (17)
Diarrhea	3 (10)	3 (10)
Nausea	1 (3)	3 (10)
Fatigue	8 (27)	4 (13)
Dyspnea	4 (13)	2 (7)
Pleural effusion	1 (3)	3 (10)
Pruritus	3 (10)	4 (13)
Noncardiac chest pain	1 (3)	3 (10)
Embolism	3 (10)	1 (3)
Hypertension	4 (13)	—
ALT increase	2 (7)	4 (13)
AST increase	2 (7)	4 (13)
Arthralgia	1 (3)	4 (13)
**Immune-related AE with incidence ≥10% at least in one arm**		
Hyperthyroidism	7 (23)	7 (23)
Hypothyroidism	3 (10)	5 (17)
Arthralgia	1 (3)	4 (13)
ALT increase	2 (7)	4 (13)
AST increase	2 (7)	4 (13)

ALT, alanine aminotransferase; AST, aspartate aminotransferase.

No patient died during preoperative immunotherapy, the postoperative 90-day mortality was 3%. Two patients (both arm A) died during extended follow-up. One patient succumbed to acute pulmonary embolism 62 days after the first dose of nivolumab. Another patient developed cryptogenic liver failure 103 days after start of study treatment with fatal outcome. A relation to nivolumab could not be excluded (Fig. 2b).

### Exploratory outcomes

#### Metabolic responses

**Extended Data Fig. 1:**
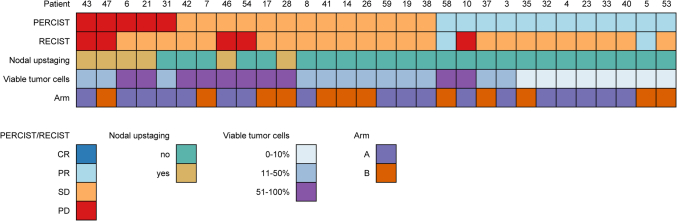
Response assessment per FDG-PET/CT and CT in relation to pathological response and nodal upstaging. Metabolic responses (PERCIST), radiographic responses (RECIST), nodal upstaging (yes, no), pathological response category (Viable tumor cells), and treatment arm (A – nivolumab, B – nivolumab/relatlimab) of 30 patients from study site Essen, who underwent FDG-PET/CT scanning following preoperative immunotherapy. One patient was excluded because surgery was aborted due to pleural carcinosis.

In 31 patients enrolled at site Essen radiographic and metabolic responses to study therapy were evaluated by positron emission tomography/computed tomography using the tracer [^18^F]-fluorodeoxyglucose (FDG-PET/CT) (Supplementary Fig. 1). The metabolic response rates per Positron Emission tomography Response Criteria In Solid Tumors (PERCIST)^28^ were 38% in both study arms (Fig. 1e). All patients with MPR had a partial metabolic response, and 8 of 12 patients (67%) with partial metabolic response had achieved MPR (Extended Data Fig. 1). By comparison of preoperative clinical and postoperative pathological tumor stage, nodal upstaging was observed in 4 of 5 patients (3 of 4 in arm A and 1 of 1 in arm B) with metabolic progression, but only in 2 of 25 patients (1 per arm) with metabolically stable disease or partial metabolic response (Extended Data Fig. 1).

#### Immune cell phenotyping

**Fig. 3:**
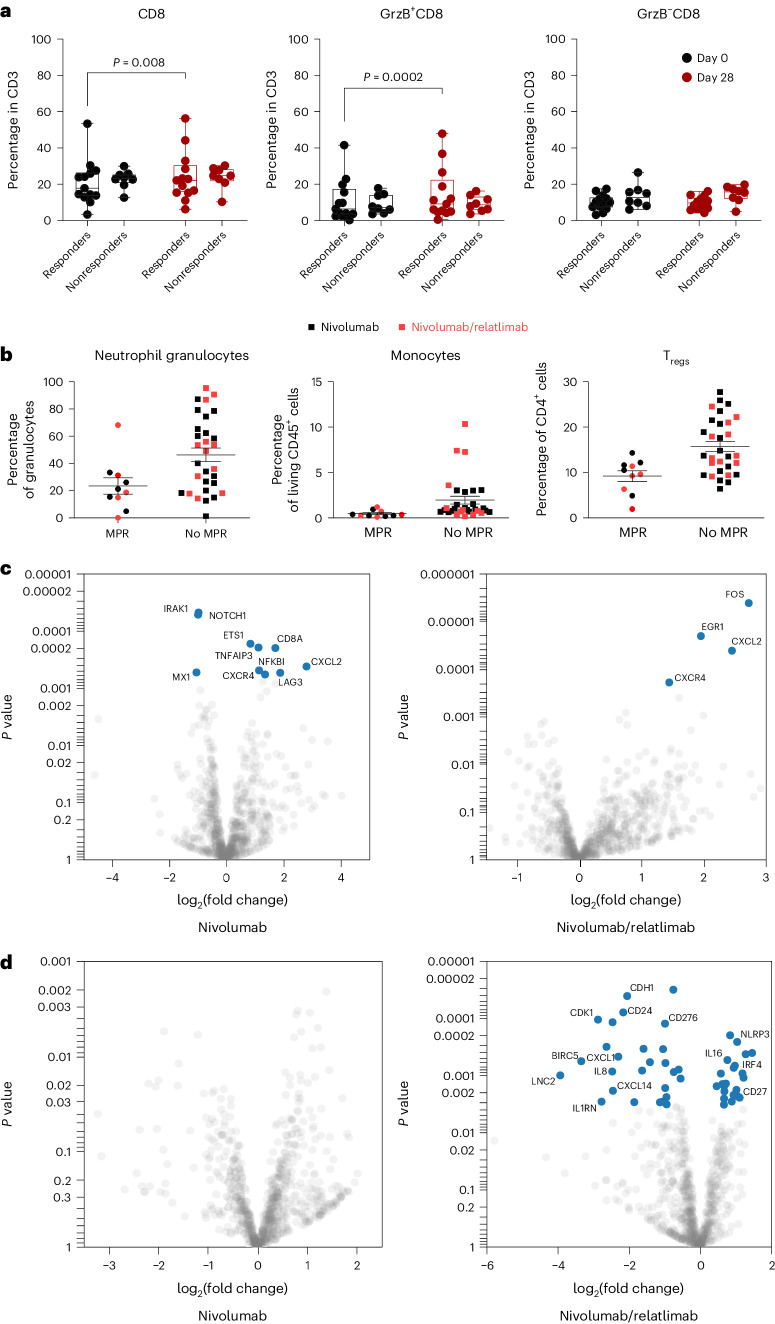
Immune cell subsets and gene expression in peripheral blood and resected tumors. **a**, Fraction of total CD8^+^ T cells (left), CD8^+^GrzB^+^ effector T cells (center) and CD8^+^GrzB^−^ T cells (right) in the peripheral blood of responding (≤50% viable tumor cells in resected tumors and lymph nodes) and nonresponding patients (>50% viable tumor cells). Each dot represents an individual patient: baseline values are in black and values at day 28 are in red. Whiskers and boxes represent the minimum, first, second and third quartiles and the maximum. Wilcoxon matched pairs signed-rank test was applied for statistical comparison. All *P* values are two-sided, no adjustments were made for multiple comparisons. **b**, Fraction of CD16^+^ neutrophil granulocytes (left), CD14^+^ monocytes (center) and CD4^+^CD25^+^ regulatory T cells (T_reg_, right) in single-cell suspensions from resected tumors. Each dot or box represents a single patient (black, nivolumab; red, nivolumab and relatlimab; MPR, ≤10% viable tumor cells in resected tumors and lymph nodes; no MPR, >10% viable tumor cells). Horizontal lines indicate the mean and s.e.m. **c**, Differential expression of immune-related and cancer pathway-related genes in response to treatment with nivolumab (left) and nivolumab and relatlimab (right) are presented as volcano plots. Significantly (FDR ≤ 0.05) upregulated (right of 0 line on *x* axes) and downregulated (left of 0 line on *x* axes) genes are depicted as blue closed circles. Selected significantly regulated genes are indicated. *P* values on the *y* axes were calculated using the two-sided quasi-likelihood F-test approach of EdgeR. **d**, Differential expression of immune-related genes and cancer pathway-related genes in resected tumors with MPR following treatment with nivolumab (left) and nivolumab and relatlimab (right) compared with resected tumors without MPR. Significantly (FDR ≤ 0.05) upregulated (right of 0 line on *x* axes) and downregulated (left of 0 line on *x* axes) genes in tumors with MPR are depicted as blue closed circles. Selected significantly regulated genes are indicated. *P* values on the *y* axes were calculated using the two-sided quasi-likelihood F-test approach of EdgeR. There was no significant interaction with MPR following nivolumab treatment.

**Extended Data Fig. 2:**
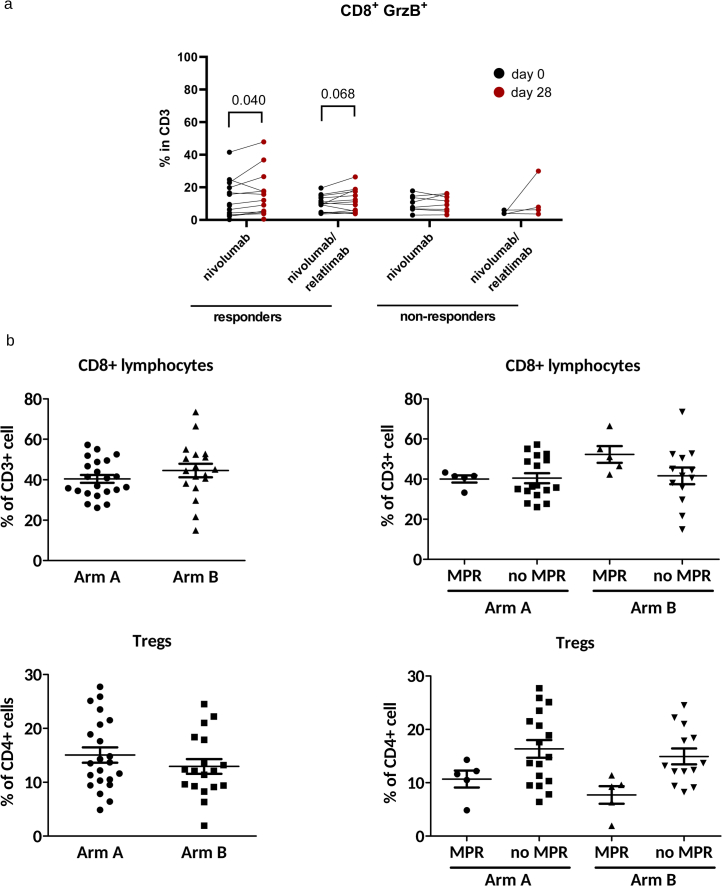
Immune cell subsets in peripheral blood and resected tumors. **a**, Induction of CD8+GrzB+ effector T cells in the peripheral blood in response to neoadjuvant nivolumab or nivolumab and relatlimab treatment. Each dot represents an individual patient, base line values in black, values at day 28 (after neoadjuvant immunotherapy) in red. Responders are defined by ≤ 50% viable tumor cells in resected tumors and lymph nodes, non-responders by >50% viable tumor cells. The Wilcoxon matched pairs signed-rank test was applied for statistical comparison. All p-values are two-sided, no adjustment was made for multiple comparisons. **b**, Characterization of infiltrating T lymphocytes in resected tumors in relation to study treatment (arm A: nivolumab, arm B: nivolumab and relatlimab) and achieving a major pathological response (MPR, ≤ 10% viable tumor cells in resected tumors and lymph nodes) or not achieving a MPR (no MPR). Each symbol represents an individual patient. Tregs – regulatory T lymphocytes. Horizontal lines indicate the mean value and standard error of the mean.

Immune cell subsets were studied by multiparametric flow cytometry in the peripheral blood (*n* = 38) (Supplementary Fig. 2a) and in resected primary tumors (*n* = 40) (Supplementary Fig. 2b,c) whenever feasible. At baseline there was no apparent difference in CD8^+^ and CD8^+^Granzyme B^+^ (GrzB^+^) peripheral blood T cells between patients with pathological response (≤50% viable tumor cells) and nonresponders. After 4 weeks of immunotherapy responders exhibited a significant increase in CD8^+^ and CD8^+^GrzB^+^ peripheral blood T cells compared with nonresponders (Fig. 3a). Comparable effects were observed in responders treated with nivolumab monotherapy (*n* = 13, *P* = 0.04) and nivolumab plus relatlimab (*n* = 13, *P* = 0.068) (Extended Data Fig. 2a). Importantly, immune cell infiltrates of resected tumors from patients with MPR contained fewer CD16^+^ neutrophil granulocytes, CD14^+^ monocytes and CD4^+^CD25^+^ regulatory T cells compared with resected lung cancers without MPR (Fig. 3b and Extended Data Fig. 2b).

#### Expression of immune- and cancer pathway-related genes

To dissect the impact of nivolumab with or without relatlimab on immune-related and cancer pathway-related gene sets, we compared the expression profiles of 15 pretreatment tumor biopsies (6 in arm A and 9 in arm B) and 43 resected lung tumors (21 in arm A and 22 in arm B). In both study arms, *CXCL2* and *CXCR4*, encoding an inflammation-associated chemokine and receptor, were strongly induced. In addition, nivolumab modulated a diverse spectrum of genes involved in inflammation, NFκB signaling and interferon response such as *NFKBI*, *TNFAIP3*, *CD8A*, *IRAK1* and *MX1*. Expression of the immune checkpoint gene *LAG3* was significantly induced by nivolumab, but not by the nivolumab/relatlimab combination (Fig. 3c). Studying resected tumors from nivolumab-treated patients in relation to MPR, a heterogeneous pattern without statistically significant changes in gene expression levels emerged. By contrast, MPR following nivolumab plus relatlimab was significantly associated with suppressed gene programs linked to granulocytes, monocytes and macrophages such as *CD24*, *CXCL1, CXCL14*, *IL8*, *MIF* and *ISG15*. Significantly upregulated genes in responders to nivolumab plus relatlimab included *NLRP3*, *CD27*, *IRF4* and *IL16*, which are involved in inflammasome and NFκB signaling, the interferon response and T cell activation. In addition, a cluster of genes associated with epithelial and cancer cells (for example, *CDH1*, *EPCAM*, *BIRC5* and *CD276*) was significantly downregulated in resected tumors with nivolumab/relatlimab-induced MPR (Fig. 3d).

#### Shaping of cancer genomes by immunotherapy

In 43 patients, pretreatment tumor biopsies, resected tumors and normal tissue of sufficient quality and quantity were obtained to longitudinally explore the mutational profiles of a comprehensive set of cancer-related genes. Tumor biopsies taken at diagnosis revealed no apparent clustering of recurring mutations in patients with or without a histopathological response (Fig. 2a). There were three patients with *EGFR*-mutated tumors (arm A: *EGFR* insertion exon 20; arm B: *EGFR* deletion exon 19 and co-mutation of *EGFR* p.G719A and p.S768I), who had 95% (arm A) and 50% viable tumor cells (both in arm B) following study therapy. No *ALK* or *ROS1* gene fusions or other oncogenic drivers susceptible to approved targeted first-line therapies of NSCLC were identified.

**Fig. 4:**
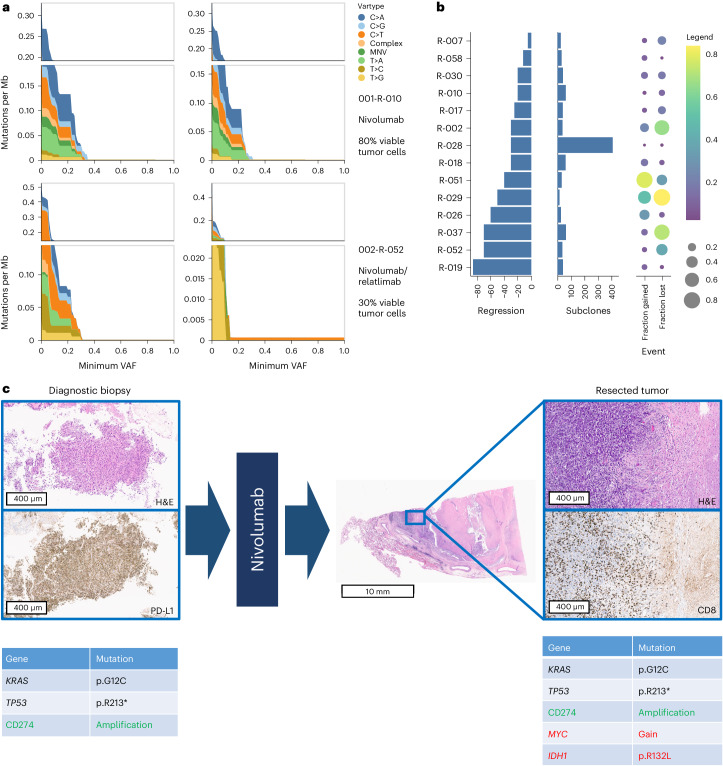
Dynamic changes in the mutational spectra in response to immunotherapy. **a**, Prevalence of mutations per megabase (Mb, *y* axes) of 500 cancer-related genes in pretherapeutic diagnostic biopsies (left) and resected tumors (right) of two exemplary patients without (001-R-010) and with response (002-R-052) to study therapy. The specific mutations (nucleotide exchanges from C to A (C>A), G (C>G) or T (C>T), from T to A (T>A), C (T>C) or G (T>G), complex nucleotide replacements (complex) or multiple nucleotide variants (MNV)) are color-coded from dark blue to yellow. The minimal VAFs are depicted on the *x* axes. **b**, Subclonal dynamics between pretherapeutic biopsies and resected tumors of 14 patients; each line depicts an individual patient. Left, pathological regression (percentage reduction of viable tumor cells) following immunotherapy. Center, estimated total number of subclones in the resected tumor. Right, fraction of subclones enriched (‘fraction gained’) and depleted (‘fraction lost’) in the resected tumors. Fractions are visualized by color (with yellow for high, purple for low), and bubble size (large for high, small for low, no bubble for zero). **c**, Selection of genomically encoded putative resistance mechanisms in one of 43 patients with pretreatment and posttreatment tumor specimens for genomic analyses. Left, representative microphotographs of the pretherapeutic diagnostic tumor biopsy stained with H&E and with an anti-PD-L1 primary antibody. DNA sequencing of the tumor biopsy revealed pathogenic mutations of *KRAS* and *TP53* and amplification of the CD274 (PD-L1)-encoding gene. Center, low magnification image of a H&E-stained section of the resected tumor showing massive necrosis, but a residual region of vital tumor cells on the left-hand margin. Right, high magnification photomicrographs representing the transition zone from necrotic tumor to residual viable tumor cells stained with H&E and with an anti-CD8 primary antibody demonstrating tumor-infiltrating T lymphocytes. DNA sequencing of the resected tumor confirmed the presence of pathogenic mutations of *KRAS* and *TP53* and amplification of the CD274 (PD-L1)-encoding gene. In addition, copy number gain of *MYC* and a pathogenic *IDH1* mutation were newly detected. A complete list of patients with enrichment of genomically encoded putative resistance mechanisms in resected tumors is presented in Supplementary Fig. 3.

Longitudinal analyses of the global mutational spectra comparing diagnostic biopsies and resected tumors at the single patient level were performed using whole-exome sequencing. These spectra appeared to be not significantly altered in resected tumors of patients who failed to substantially respond to preoperative ICI therapy, whereas reduced mutational diversity was observed in tumors with deeper pathological responses (Fig. 4a). Pretreatment and posttreatment samples from 14 patients met stringent prerequisites for inferring the dynamics of subclonal diversity (see Methods for details). This revealed strong evidence of genomic remodeling in immunotherapy responders. Mutational spectra of resected tumors from patients with deeper pathological response to immune checkpoint blockade exhibited both enrichment and depletion of subclones, whereas some tumors were skewed toward reduced diversity (Fig. 4b). In selected cases enrichment of cancer gene mutations, such as copy number gain of *MYC* and *KRAS*, and pathogenic variants of *IDH1* and *STK11*, was observed in residual tumor cells following study therapy (Fig. 4c and Supplementary Fig. 3).

## Discussion

Immunotherapy with antibodies blocking the immune checkpoint molecules PD-1, PD-L1 and CTLA-4 has become standard of care for patients with metastatic NSCLC not harboring oncogenic mutations of *EGFR*, *ALK*, *ROS1* or *RET*^21,22,29–34^. Consequently, their potential to reduce the risk of disease recurrence and death following curatively intended therapies such as chemoradiotherapy^35^ and surgery is explored in locally advanced or localized NSCLC. Preoperative ICI therapy is a particularly attractive strategy. Short-course treatment with antibodies blocking PD-1 or PD-L1 alone or combined with platinum-based chemotherapy may induce deep pathological responses, which are associated with favorable survival outcomes^36,37^. Although there is a clear interaction between ICI and chemotherapy in terms of efficacy, the added toxicities of chemotherapy are not required in all NSCLC patients to unfold the full curative potential of ICI treatment.

Against this background, combined targeting of further immune checkpoints in addition to the PD-1/PD-L1 axis is a rational next step in the development of preoperative immunotherapy of NSCLC. The phase 2 study NEOSTAR^10^ randomized 21 patients to three preoperative doses of nivolumab (anti-PD-1) and a single dose of ipilimumab (anti-CTLA-4). Of 16 patients (76%) subsequently undergoing resection, 6 achieved complete pathological responses (29%), and grade ≥3 toxicities were reported in 10%. This was further explored in the phase 3 study CheckMate 816, which in its third arm randomized 113 patients with resectable NSCLC to preoperative nivolumab and ipilimumab^13^. Of those, 74% proceeded to definitive surgery, which revealed complete pathological responses in 23 patients (20%). Grade ≥3 toxicities were observed in 20% of patients. Although the early efficacy outcomes of both studies are promising, the toxicities and relatively low fraction of operated patients leave room for improvement.

The current study, NEOpredict-Lung, aims to establish the feasibility of combining the PD-1 blocking antibody, nivolumab, and the LAG-3 blocking antibody, relatlimab, in preoperative treatment of NSCLC patients. When the study was conceived and initiated this ICI combination was still in clinical development. Therefore, patients were randomized to nivolumab with or without relatlimab, with monotherapy serving as a reference for safety, feasibility, efficacy and exploratory endpoints. The study was not designed for formal statistical comparison of both treatment arms. In the meantime, nivolumab plus relatlimab combination therapy has been globally approved for the treatment of patients with unresectable or metastatic cutaneous melanoma, thus supporting the rationale and providing an extensive safety database^25^. With all randomized patients reaching the primary study endpoint, that is proceeding to surgery within 43 days of initiation of ICI therapy, NEOpredict-Lung confirms the feasibility of both study arms. Achieving R0 resections in 95% of patients compares favorably with other studies of ICI-based neoadjuvant treatment in NSCLC with operation rates mostly around 80%^9,11–18^. The safety of preoperative nivolumab plus relatlimab was supported with no apparent difference in overall frequency and severity of AEs, treatment-related AEs and immune-related AEs compared with the reference nivolumab (Table 2).

With respect to secondary efficacy endpoints, the study NEOpredict-Lung has several limitations. First, the moderate sample size and study design preclude formal assessment of clinical efficacy, and appreciation of an additional contribution of relatlimab to pathological and radiographic response rates and survival endpoints. It seems apparent that deeper histopathological responses to nivolumab with or without relatlimab cluster in patients with PD-L1-positive NSCLC (Fig. 2a). Because randomization was not stratified for PD-L1 status, the imbalanced distribution of patients with PD-L1 highly positive NSCLC between study arms (Table 1) may have skewed deep pathological response rates in favor of nivolumab monotherapy. The exclusion of patients with extensive mediastinal lymph node metastases may have contributed to the excellent surgical results and early survival outcomes in NEOpredict-Lung compared with other studies of preoperative ICI combinations.

Obtaining comprehensive cellular and molecular portraits of lung cancers within their tissue context is key to advancing the mechanistic understanding of response and resistance to ICI therapy. Against this background, administering ICI before lung cancer surgery is an important step allowing exploratory analyses of treatment-induced biological processes. The study NEOpredict-Lung provides insights into potential mechanisms of resistance to ICI therapy in NSCLC. The patient cohort covered the entire spectrum of histopathological responses in both treatment arms (Fig. 2a). This enabled exploratory correlation of translational endpoints with patient outcomes. Immune cell phenotyping demonstrated an increase in CD8^+^GrzB^+^ effector T cells in the peripheral blood of responding patients (Fig. 3a), which is in line with findings from other studies. Likewise, correlative studies in NEOSTAR demonstrated greater infiltration of CD3^+^CD8^+^GrzB^+^ T lymphocytes upon combined blockade of PD-1 and CTLA-4 (ref. ^10^). Although these patterns are consistent across both trials, methods and endpoints of correlative analyses were not aligned precluding direct comparison.

The negative correlation of histopathological response with the intratumoral representation of suppressive immune cell subsets is a key finding of our study. This is orthogonally supported at the cellular level by the enrichment of granulocytes, monocytes and regulatory T cells in resected tumors not achieving a MPR (Fig. 3b), and at the molecular level by gene expression analyses. In tumors with MPR following nivolumab plus relatlimab there was a significant suppression of gene programs linked to granulocytes, monocytes and macrophages (Fig. 3d). These findings provide important leads for further mechanistic studies and toward the nomination of biologically rational therapeutic targets for combination therapies. Assessing dynamic changes in the expression of immune-related genes between pretreatment biopsies and resected tumors, an interesting pattern emerged. Reassuringly, *CXCL2* and *CXCR4* encoding inflammation-associated chemokine proteins were most significantly induced by nivolumab with or without relatlimab (Fig. 3c). In addition, nivolumab associated with a very diverse spectrum of genes that were significantly upregulated or downregulated. By contrast, the change in gene expression patterns following nivolumab plus relatlimab therapy was more homogenous. This is also reflected by the absence of a significant correlation of gene expression changes in nivolumab responders (Fig. 3d). These findings argue for a more consistent and directed immune activation by combined treatment with nivolumab plus relatlimab, which may provide opportunities for rational triplet combinations that may have the capacity to expand the responding patient population.

Another key observation of NEOpredict-Lung is how rapidly ICI-induced immune activation may shape the individual genomic landscapes of NSCLC (Fig. 4). Our findings suggest that in a subgroup of patients nivolumab with or without relatlimab failed to reinvigorate an immune response that significantly impacts on clonally diverse tumors. In another subgroup four weeks of nivolumab with or without relatlimab were sufficient to empower complete immune eradication of lung cancers, which precluded meaningful longitudinal genomic analyses. Interestingly, in a third subgroup of patients who achieved substantial but not complete histopathological responses the enrichment of apparently resistant clones and depletion of sensitive clones was observed under the selective pressure mounted during preoperative ICI therapy. The latter hypothesis is corroborated by selected cases in which emergence of biologically plausible genomic resistance mechanisms, such as copy number gain of *MYC* and *KRAS*, and pathogenic variants of *IDH1* and *STK11*, is observed by longitudinal genome sequencing (Fig. 4c and Supplementary Fig. 3).

In conclusion, the study NEOpredict-Lung establishes the feasibility and safety of preoperative treatment with nivolumab and relatlimab in patients with resectable NSCLC stages IB, II and IIIA. Based on early signals of clinical and biological activity obtained with this and another recently reported study in patients with metastatic NSCLC^38^ further exploration of dual targeting of PD-1 and LAG-3 in NSCLC is clearly warranted.

## Methods

### Clinical study

#### Patients

Adult patients with histologically or cytologically confirmed NSCLC eligible for anatomic resection were enrolled. Clinical stages IB, II and selected stage IIIA (T3 N1, T4 with satellite nodule in the same lung N0/N1, selected T1a–T2b N2 cases considered suitable for primary surgical approach by the multidisciplinary tumor board) according to the Union International Contre le Cancer (UICC) eighth edition were eligible. Additional inclusion criteria (see Supplementary information for full study protocol) are women and men ≥18 years of age, Eastern Cooperative Oncology Group performance score ≤1, exclusion of extensive mediastinal lymph node metastases (multilevel N2, N3), exclusion of distant metastases, measurable target tumor before immunotherapy using standard imaging techniques, sufficient pulmonary function to undergo curative lung cancer surgery (percentage of predicted forced expiratory volume at 1 s (ppFEV1) > 30%, percentage of predicted diffusion capacity of the lung for carbon monoxide (ppDLCO) > 30%, percentage of predicted maximal oxygen consumption (ppVO_2_max) ≥ 10 ml min^−1^ kg^−1^ (if cardiopulmonary exercise testing was mandated per local guidelines)), adequate hematological, hepatic and renal function parameters, sufficient cardiac left ventricular function defined as left ventricular ejection fraction ≥50% documented either by echocardiography or multigated acquisition (MUGA) scan, ability and willingness to provide written informed consent and to comply with the study protocol and with the planned surgical procedures. Gender was determined based on self-report.

Exclusion criteria (see Supplementary information for full study protocol) are: active or history of autoimmune disease or immune deficiency; subjects with a condition requiring systemic treatment with either corticosteroids (>10 mg daily prednisone equivalents) or other immunosuppressive medications within 14 days of study drug administration; subjects who have undergone organ transplant or allogeneic stem cell transplantation; ppFEV1 < 30%, ppDLCO < 30%, ppVO_2_max < 10 ml min^−1^ kg^−1^ (if cardiopulmonary exercise testing was mandated per local guidelines); uncontrolled or significant cardiovascular disease (including myocardial infarction, stroke or transient ischemic attack, uncontrolled angina, clinically significant arrhythmias, QTc prolongation >480 ms, pulmonary hypertension); history of other clinically significant cardiovascular disease (including cardiomyopathy, congestive heart failure, pericarditis, pericardial effusion, coronary artery stent occlusion, deep venous thrombosis); cardiopulmonary disease-related requirement for daily supplemental oxygen; subjects with a history of myocarditis, regardless of etiology; elevated troponin T or I; active neurological disease; active malignancy or previous malignancy within the past 3 years; known history of positive test for human immunodeficiency virus (HIV-1 and HIV-2) or known acquired immunodeficiency syndrome; any positive test result for hepatitis B virus or hepatitis C virus (HCV) indicating presence of virus, for example hepatitis B surface antigen (Australia antigen) positive or hepatitis C antibody (anti-HCV) positive (except if HCV RNA negative); any other disease, metabolic dysfunction, physical examination finding or clinical laboratory finding that contraindicates the use of an investigational drug, may affect the interpretation of the results or may render the patient at high risk from treatment complications; receipt of live attenuated vaccine within 30 days before the first dose of study medication; peripheral neuropathy National Cancer Institute Common Terminology Criteria for Adverse Events grade ≥2; history of gastric perforation or fistulae in past 6 months; serious or nonhealing wound, ulcer or bone fracture within 28 days before enrollment; major surgery within 28 days before enrollment except staging mediastinoscopy, diagnostic video-assisted thoracoscopic surgery (VATS) or implantation of a venous port-system; any other concurrent preoperative antineoplastic treatment including irradiation, pregnant or breastfeeding women; insufficient cardiac left ventricular function defined as left ventricular ejection fraction <50% by echocardiography or MUGA scan; confirmed history of encephalitis, meningitis or uncontrolled seizures in the year before informed consent; subjects with a history of severe toxicity or life-threatening toxicity (grade 3 or 4) related to previous immune therapy (for example anti-CTLA-4 or anti-PD-1/PD-L1 treatment or any other antibody or drug specifically targeting T cell co-stimulation or immune checkpoint pathways) except those that are unlikely to reoccur with standard countermeasures (for example, hormone replacement after endocrinopathy); history of severe or life-threatening (grade 3 or 4) infusion-related reactions to previous immuno therapy; previous treatment with LAG-3 targeting agent; participation in another interventional clinical study within the past 3 months before inclusion or simultaneous participation in other clinical studies; previous treatment with nivolumab or relatlimab; previous immunotherapy for lung cancer; criteria that in the opinion of the investigator preclude participation for scientific reasons, for reasons of compliance or for reasons of the subject’s safety; or any contraindications against nivolumab or relatlimab.

#### Study design and treatment

NEOpredict-Lung (NCT04205552) is an open-label, randomized phase 2 trial (see Supplementary information for the version of the study protocol pertinent to this report). This manuscript reports results from arms A and B of the study, which treated patients with two doses of nivolumab (240 mg every 14 days per intravenous infusion, arm A) or nivolumab and relatlimab (240 and 80 mg, respectively, every 14 days per intravenous infusion, arm B). The dose and schedule of nivolumab and nivolumab plus relatlimab were selected to align with the biweekly administration of nivolumab in other studies of preoperative ICI combinations in NSCLC patients, such as NEOSTAR^10^ and CheckMate 816 (ref. ^13^). It is supported by findings of the ongoing study RELATIVITY-020 (ref. ^39^), which explores multiple doses and schedules of relatlimab-based combinations.

The study was not designed to formally compare both treatment arms. No gender analysis was performed because of the limited cohort sizes and the nature of the study.

Patients were randomly assigned (1:1) via an interactive web response system provided by Alcedis GmbH (https://www.alcedis.de/en); there was no stratification or blinding. Patients were treated for a maximum of two cycles (14 days each), which was followed by standard of care surgery and, if clinically indicated, postoperative medical therapy and/or radiotherapy. Surgery and postoperative treatments were not part of the clinical study intervention. All patients are followed up to 12 months within the study protocol. Subsequent follow-up is provided within standard of care.

#### Endpoints

The primary study endpoint is the number of patients proceeding to curatively intended surgery of NSCLC within 43 days of the initiation of study therapy.

Secondary endpoints include: the objective response rate (RECIST 1.1) before surgery; the pathological response rate (complete pathological responses defined as the absence of viable tumor cells on routine H&E staining of resected tumors and lymph nodes, and rate of MPRs defined as 10% or less viable tumor cells on routine H&E staining of resected tumors); the R0 resection rate; the DFS rate at 12 months per RECIST 1.1; the OS rate at 12 months; the safety and tolerability of preoperative immunotherapy; and morbidity and mortality within 90 days of surgery.

Exploratory endpoints are assessed in tumor and lymph node samples, blood cells, plasma and serum.

All primary and secondary endpoints were assessed in the intention-to-treat population and in the full analysis set.

Clinical data are captured in the clinical database using a proprietary electronic case report system provided by Alcedis GmbH (https://www.alcedis.de/en).

#### Assessments

Radiographic and nuclear imaging assessments at baseline were conducted within standard of care at the study sites. Specifically, all 60 patients underwent whole-body imaging by FDG-PET/CT. For exclusion of brain metastases, 41 patients underwent contrast-enhanced brain magnetic resonance imaging (MRI) scanning, 18 patients underwent contrast-enhanced brain CT scanning (because of contraindications or intolerance of MRI imaging or unavailability of an MRI slot within the protocol-defined screening period). In one patient with stage IB NSCLC no brain imaging was performed as per Dutch guidelines. All patients underwent CT or PET/CT imaging immediately before surgery. Radiographic response was evaluated at the study sites following RECIST 1.1. Exploratory analyses were conducted on nuclear imaging data acquired before surgery.

Baseline assessments included the collection of tumor tissue samples for centrally performed exploratory analyses. Diagnostic tumor tissue was obtained by endobronchial ultrasound-guided biopsy (31 patients), CT-guided transthoracic biopsy (17 patients) or by other approaches including bronchoscopy-guided forceps biopsy and miniprobe/navigation-guided biopsy (13 patients). For mediastinal staging, 47 patients underwent systematic endobronchial ultrasound including sampling of suspicious lymph nodes, and 11 patients had staging mediastinoscopy.

Histology and biomarker studies were conducted within standard of care at the study sites. PD-L1 expression by tumor cells was assessed locally using the primary antibody clone 22C3 (DAKO/Agilent M3653) following validated protocols with continuous external quality assurance (QUIP, UK NEQAS, NordiQC).

Additional tumor tissue samples were collected during surgery, and blood samples were collected at protocol-defined time points.

#### Statistical analyses

Based on published results of a study with preoperative nivolumab^9^ each study arm included up to 30 evaluable patients with the expectation that at least 26 of 30 patients treated in each study arm will undergo curatively intended surgery within 6 weeks of initiation of study treatment. At maximum 4 of 30 patients may experience a delay of curatively intended surgery beyond day 43 (with study treatment being administered on day 1), either because of toxicities or disease progression, to declare the study arm feasible. Continuous monitoring of prespecified stopping boundaries was applied to facilitate early termination of nonfeasible study arms to reduce patient risks. Details can be reviewed in the clinical study protocol (Supplementary information).

All secondary parameters were evaluated in an explorative or descriptive manner, providing means, medians, ranges, standard deviations and/or confidence intervals.

#### Trial oversight

The protocol and amendments were approved by the responsible ethics committees and competent regulatory authorities at each participating study site and country. In the legislature of the study sponsor and study site Essen the Ethics Committee of the Medical Faculty of the University Duisburg-Essen, Essen, Germany, granted primary approval on 10 September 2019 (19-8828-AF). The competent regulatory authority in the legislature of the study sponsor and study site Essen, the Paul-Ehrlich-Institut (Federal Institute for Vaccines and Biomedicines), Langen, Germany, granted primary approval on 27 November 2019 (EudraCT-Nr. 2109-007278-29, Vorlage-Nr. 3834/01). For study site Hasselt, approval was granted by the Ethics Committee OLV Ziekenhuis VZW, Aalst, Belgium (EudraCT-Nr. 2109-007278-29 Pilot 262-SM001, Reference 202/082), and the Federal Agency for Medicines and Health Products, Brussels, Belgium (EudraCT-Nr. 2109-007278-29 Pilot 262, 1240640 M). For study site Amsterdam, approval was granted by the METC—The Netherlands Cancer Institute, Antoni van Leeuwenhoek (NKI-AVL), Amsterdam, The Netherlands (NL72532.031.20), and by the Centrale Commissee Mensgebonden Onderzoek, The Hague, The Netherlands (Decree NL72532.031.21 CA).

The study was conducted according to the principles of the Declaration of Helsinki and the International Conference on Harmonization Good Clinical Practice guidelines.

All patients provided written informed consent before enrollment. The study is sponsored by the University Hospital Essen and was designed by employees of the sponsor, who were also study investigators.

A data safety monitoring committee, which is independent of the sponsor and the study investigators, reviewed all safety and efficacy data, including radiographic and pathological response data.

The clinical data were collected by the investigators, analyzed by statisticians employed by a contract research organization commissioned by the sponsor, and interpreted by the authors. Authors had full access to the data and are responsible for all content and editorial decisions.

### Metabolic hybrid imaging

As per national and international practice guidelines, patients received FDG-PET/CT (or PET) at initial staging. Patients treated at study site Essen underwent a second FDG-PET/CT scan before surgery to confirm curative resectability. Images were acquired at a median of 4 days (range 1–29 days) before surgery. Imaging data were collected on three different PET/CT scanner types (Biograph Vision 600 (Siemens Healthineers), Biograph mCT (Siemens Healthineers), Vereos (Philips Healthcare)). It was ascertained that each individual patient underwent both scans on the same scanner type. Data acquisition started 67 ± 18 min (PET/CT 1) and 72 ± 12 min (PET/CT 2) after injection of 305 ± 58 MBq FDG (PET/CT 1) and 280 ± 58 MBq FDG (PET/CT 2), respectively. Patient handling and data processing were performed as detailed elsewhere^40^. After attenuation correction metabolic response rates were estimated according to PERCIST 1.0 (ref. ^28^).

### Phenotyping of peripheral blood T cells

T cell phenotypes were determined by multiparametric flow cytometry. Briefly, cryopreserved peripheral blood mononuclear cells were thawed and rested overnight in RPMI medium supplemented with 10% FCS, 100 U ml^−1^ penicillin and 100 µg ml^−1^ streptomycin (PAA Laboratories) at 37 °C in a 5% CO_2_ atmosphere. Antibody staining of cell surface molecules (30 min, 4 °C) was followed by fixation and permeabilization for staining of intracellular markers (30 min, 4 °C). Stained samples were analyzed using a Gallios flow cytometer (Beckman Coulter) and Kaluza software (Beckman Coulter). Antibodies and gating strategy are depicted in Supplementary Fig. 2a.

### Phenotyping of immune cell subsets in resected tumors

#### Dissection of resected tumors

Tumor tissue was put in 1 ml of digestion medium (Dulbeccoʼs modified Eagleʼs medium/F12/HEPES solution supplemented with penicillin/streptomycin and 1% BSA and containing collagenase, hyaluronidase and DNAse I) and cut into small pieces. To facilitate dissociation the tissue was incubated for 40 min at 37 °C and pipetted every 10 min during the incubation period. The resulting cell suspension was transferred to a 50-ml centrifuge tube and centrifuged at 300*g* for 10 min at ambient temperature. The pellet was resuspended in trypsin/EDTA and incubated for 5 min at ambient temperature. After inactivation of the trypsin by Dulbeccoʼs modified Eagleʼs medium/F12/HEPES solution containing 10% FCS, the cell suspension was again triturated and filtered through a 40-µm cell strainer. After washing the filter with 50 ml of phosphate-buffered saline (PBS) the cells were centrifuged at 400*g* for 5 min at ambient temperature. Following one more washing step with PBS, cell number and viability was measured using the NucleoCounter NC-3000 and one to two million cells per vial were cryopreserved in FCS-containing 10% DMSO.

#### Flow cytometry

The cryopreserved tumor cell suspensions were analyzed in batches using two panels of antibodies. The staining method, antibodies and gating strategy for T lymphocyte subsets (Supplementary Fig. 2b) have been described previously^41^. Myeloid immune cells were detected using a separate antibody panel and gating strategy (Supplementary Fig. 2c). Flow cytometry was run on a CytoFLEX LX (Beckman Coulter) using the CytExpert v.2.3 software. Final data analysis was performed using FlowJo Software v.10 (Tree Star).

### Gene expression analyses

#### RNA isolation and quantification

For nucleic acid isolation, two to four sections each 10-µm thick (depending on sample size) from the respective formalin-fixed paraffin-embedded (FFPE) tissue sample were used. In total, RNA isolation could be performed on 46 resected tumors as well as 17 paired biopsies. Isolation procedures have been carried out semiautomatically on the Maxwell purification system (Maxwell RSC RNA FFPE Kit; Promega, cat. no. AS1440). All steps were performed following the respective protocol provided by the manufacturer. Total RNA was eluted in 50 µl RNase-free water and quantified using the RNA broad-range assay on a Qubit 2.0 fluorometer (Life Technology). One microliter of sample isolate volume was diluted for each quantification. RNA was stored at −80 °C until further use.

#### NanoString CodeSet design

Fluorescently barcoded RNA probes were synthesized and provided by NanoString. In total, gene expression was quantified using the dedicated PanCancer Immune Profiling panel as well as the PanCancer Pathway panel. Both panels consisted of the identical 40 reference and 770 individual target genes. The PanCancer Pathway panel comprises key players of the Notch, APC (Wnt), Hedgehog, transforming growth factor β, MAPK, STAT, PI3K and RAS signaling pathways as well as chromatin modification, transcriptional regulation, DNA damage control, cell cycle and apoptosis. The PanCancer Immune Profiling panel comprises targets associated with the various immunological processes and pathways of a host anti-cancer immune response. In total, both panels combined cover 1,398 different genes. For both panels, one sample served as a blank.

#### Digital gene expression analysis by hybridization

Digital gene expression analysis was performed on the NanoString nCounter platform, utilizing the NanoString MAX/FLEX system. A minimum of 100 ng of total RNA sample input was hybridized to the probes for 21 h at 65 °C. Subsequent cartridge preparation was performed in a NanoString PrepStation using the high-sensitivity protocol. Finally, the cartridge was scanned on the DigitalAnalyzer (NanoString) at 555 fields-of-view.

#### Gene expression analysis

NanoString data was normalized and cleaned using NanoTube (v.1.6.0)^42^, entailing three steps. First, counts were scaled by comparing the geometric mean of positive control features between samples. Second, genes in which at least 50% of samples are <2 s.d. above the mean of negative controls were removed. Third, counts were scaled by comparing the geometric mean of housekeeping genes between samples. Afterwards, differential expression analysis was performed using the quasi-likelihood *F*-test approach of EdgeR (two-sided, v.3.40.0)^43^. First, genes differentially expressed between sample types (resected tumor versus biopsy) were determined, while correcting for additive batch effects induced by pathological response (MPR = 1/0) and tumor classification (LUAD, lung squamous cell carcinoma, large-cell neuroendocrine carcinoma, sarcomatoid). Second, genes differentially expressed between MPR and no MPR were determined separately within each sample type and study arm. Reproducibility was ensured by implementing above analysis as a Snakemake^44^ workflow.

### Genome sequencing

#### DNA isolation and quantification

For tumor samples, one to four FFPE sections (10-µm thick, number depending on sample size) were lysed for genomic DNA isolation. Isolation was performed semiautomatically on the Maxwell purification system (Maxwell RSC DNA FFPE Kit; Promega, cat. no. AS1450) as specified by the manufacturer. DNA was eluted in 50 µl of RNase-free water and quantified fluorescently for library preparation using a Qubit 2.0 fluorometer (Life Technology) with its appertaining DNA broad-range assay. Corresponding normal DNA was isolated from blood or peripheral blood mononuclear cells using routinely available QIAGEN technology. DNA was stored at −20 °C before use.

#### Sequencing and genomic variant calling

Whole-exome sequencing was performed using the Twist Human Core + RefSeq + Mitochondrial Panel (Twist Bioscience), and 2 × 100-bp fragment sizes were sequenced using a NovaSeq6000 (Illumina). Demultiplexing of sequenced reads was achieved using bcl2fastq (v.2.2). Further data analysis was performed using our open-source Snakemake workflow dna-seq-varlociraptor (v.3.24, https://github.com/snakemake-workflows/dna-seq-varlociraptor), entailing the following steps. Adapter trimming was performed using Cutadapt (v.4.1, 10.14806/ej.17.1.200). Quality was monitored using MultiQC (v.1.14)^45^ including FASTQC (v.0.11.9, https://www.bioinformatics.babraham.ac.uk/projects/fastqc/), Somalier (v.0.2.18)^46^ and samtools (v.1.14)^47^. Reads were mapped to GRCh38 using bwa-mem (v.0.7.17, 10.48550/arXiv.1303.3997) and deduplicated using Picard-Tools (v.2.26). Base qualities were recalibrated using GATK (v.4.2)^48^. Single nucleotide variants and small indels were detected using Freebayes (v.1.3.6, 10.48550/arXiv.1207.3907) and classified into events of interest (somatic in biopsy or resection, germline) using Varlociraptor (v.8.3)^49^. Variant calls were distinguished from noise by controlling the (Bayesian) local false discovery rate (FDR) using Varlociraptor. Variant annotation (with impact, previous knowledge) was performed using VEP (v.109.3)^50^. Extraction of variants of interest was performed using vembrane (v.1.0)^51^. Specifically, for Fig. 2a, variants were filtered to be nonsynonymous, having a REVEL score >0.5 if available (that is, being predicted as pathogenic), having a gnomad allele frequency <0.2, being not marked as benign or likely benign in ClinVar and impacting one of the TCGA LUAD 500 cancer genes. Missing whole-exome sequencing data was complemented with results from panel sequencing (TSO500) whenever available. To identify genes that had altered variant allele frequencies (VAFs) comparing the diagnostic biopsy and the resected tumor, genes defined by oncobk (https://www.oncokb.org/cancer-genes) were inspected. To adjust for the different tumor cell content between biopsies and resected tumors, probabilities were calculated that the variants were not present in the normal sample of the same patient and that the VAF had changed before surgery. Only variants that were not marked by ClinVar as benign or likely benign and had a REVEL score >0.7 are reported in Supplementary Fig. 3.

### Inference of subclonal diversity

#### Tumor purity estimation

Previous estimates *p*_1_ and *p*_2_ of the tumor purity of samples from resected tumors were obtained by two independent pathologists evaluating sections stained with H&E. For the other samples, a posterior estimate of the tumor purity of each sample was obtained as follows. We plotted the somatic VAF distribution of the pretherapeutic biopsy and the resected tumor samples of each patient. For this, the maximum a posteriori allele frequency estimates provided by Varlociraptor without adjusting for purity were used (that is, no sample contamination assigned, see https://varlociraptor.github.io/docs/calling). The expectation is that without copy number variants any somatic variant may at most have a VAF equal to the tumor purity. Read sampling variance and copy number variation can generate peaks beyond the tumor purity. For resection samples, we proceeded as follows: Let *v* be the highest VAF of the distribution or a threshold for which higher VAFs could as well be explained by sampling or copy number variation. If *v* was consistent with the previous estimates (that is, within the interval [*p*_1_,*p*_2_]) and the previous estimates were agreeing to a sufficient degree (*p*_2_ − *p*_1_ ≤ 0.2) we reported *v* as the posterior purity. Otherwise, we considered the posterior purity as unknown (28 of 56 cases). For samples in which the resected tumor had a posterior purity, we compared the distribution of the pretherapeutic biopsy and the resected tumor, and inferred a posterior estimate by scaling the biopsy distribution to match the shape of the resection distribution. Such scaling was possible in all investigated cases.

#### Subclonal diversity

For patients with posterior purity estimates, subclonal diversity was visualized in the following way. During tumor evolution, each somatic mutation that does not lead to cell death can be seen as an event generating a new subclone. We made the simplifying assumption that each nonlethal somatic mutation during development of the tumor generates one new subclone. Thus, the number of somatic variants can be seen as a proxy for the number of subclones, and each somatic variant can be considered as a representative of the subclone that originates in it. Note that this neglects the fact that multiple somatic variants can occur during one cell division. However, under the assumption that all considered samples have a similar somatic mutation rate, the subclone counts obtained would still be proportional to the true number of subclones, and thereby comparable across patients.

Thus, for each patient, we obtained the sufficiently relevant subclones by considering variants with posterior probability ≥0.95 according to Varlociraptor for being somatic in the pretreatment biopsy or in the resected tumor, and purity adjusted VAF ≥ 0.1. To be able to be certain that a variant is detectable in both the pretreatment biopsy and the resected tumor, we further filtered them such that there would be an expectation that they would be represented by at least two reads if occurring at the same frequency in the respective other sample (pretreatment biopsy for resected tumor; resected tumor for pretreatment biopsy). Patients in whom both pretreatment biopsy and resected tumor had no such somatic variants/subclones after filtering were omitted because they would not allow any statement about subclonal gains and losses. Variants with VAF = 0.0 in the resected tumor but VAF ≥ 0.1 in the pretreatment biopsy were then counted as ‘lost subclones’ following study therapy. Variants with VAF = 0.0 in the pretreatment biopsy but VAF ≥ 0.1 in the resected tumor were counted as ‘gained subclones’ following study therapy. Note that because the pretreatment biopsy may not represent the entire primary tumor, a ‘gain’ is not distinguishable from enrichment of a variant that was spatially missed in the biopsy.

### Reporting summary

Further information on research design is available in the Nature Portfolio Reporting Summary linked to this article.

## Online content

Any methods, additional references, Nature Portfolio reporting summaries, source data, extended data, supplementary information, acknowledgements, peer review information; details of author contributions and competing interests; and statements of data and code availability are available at 10.1038/s41591-024-02965-0.

### Supplementary information

Supplementary Information Supplementary Figs. 1–3 and Clinical Study Protocol.

Reporting Summary

## Data Availability

The study protocol is provided with the Supplementary Information. Once the study is formally completed, a Clinical Study Report with tabulated data listings is prepared, which will be considered for sharing upon request from qualified scientists, if there is legal authority to share the data and there is no likelihood of participant re-identification. De-identified raw data from gene expression profiling and whole-exome sequencing have been deposited in the European Genome-Phenome Archive (EGA) with accession number EGAS00001007753. Requests should be submitted to the Office of Data Governance of the study sponsor, University Hospital Essen (https://www.uk-essen.de/), which also serves as Data Access Committee (DAC). Responses can be expected within 4 weeks.

## Code availability

All code developed and used in this study is open source. The Snakemake workflows for whole-exome sequencing analysis and NanoString nCounter gene expression analysis can be found at https://zenodo.org/records/10838511 (ref. ^52^) and https://zenodo.org/doi/10.5281/zenodo.10838907 (ref. ^53^).
