# Supplementary material for: Neoadjuvant nivolumab with or without relatlimab in resectable non-small-cell lung cancer: a randomized phase 2 trial
Source: Nat Med. 2024 Apr 30;30(6):1602–11. doi: 10.1038/s41591-024-02965-0 (PMC11186754; doi:10.1038/s41591-024-02965-0)
Supplement: Supplementary file 1 — Supplementary Figs. 1–3 and Clinical Study Protocol. [file 41591_2024_2965_MOESM1_ESM.pdf]

nature medicine

Article https://doi.org/10.1038/s41591-024-02965-0

# Neoadjuvant nivolumab with or without relatlimab in resectable non-small-cell lung cancer: a randomized phase 2 trial

In the format provided by the authors and unedited

001-R-037 LUAD Nivolumab + relatlimab

001-R-006 LUAD Nivolumab

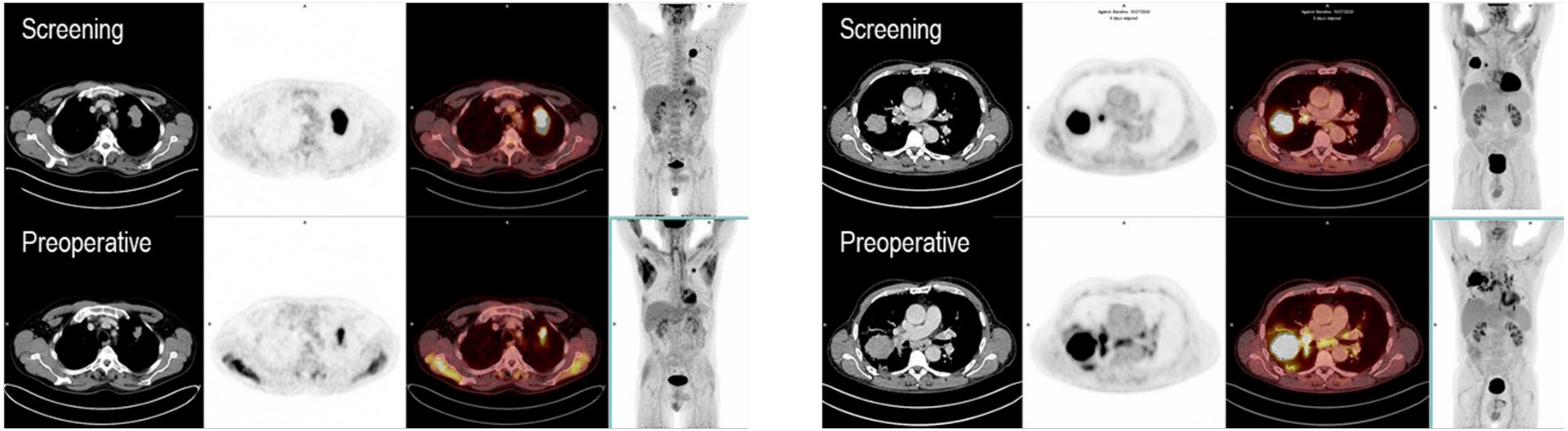


**001-R-037**

Preoperative stage: cT3 cN0 M0

PERCIST response: partial response
Postoperative stage: ypT1a ypN0 M0 R0
Pathological response: 30% viable tumor cells

**001-R-006**

Preoperative stage: cT3 cN0 M0

PERCIST response: progressive disease
Postoperative stage: ypT3 ypN2 M0 R0
Pathological response: 80% viable tumor cells

**Suppl. Fig. 1 | Metabolic response assessment per FDG-PET/CT.**
Deidentified imaging data from exemplary patients with lung adenocarcinomas stage with partial metabolic response (001-R-037) and metabolically progressive disease (001-R-006) following neoadjuvant immunotherapy. Both patients have consented publication. Representative images (computed tomography – left panels, positron emission tomography – left center panels, fusion images – right center panels, topograms – right panels) taken during screening/staging (upper lines) and following study therapy prior to surgery (lower lines) are shown. Preoperative clinical stages, metabolic response per PERCIST, postoperative histopathological stages, and pathological response to study therapy are shown. In patient 001-R-006 ipsilateral (N2) and contralateral (N3) lymph nodes were sampled preoperatively by EBUS-TBNA and intraoperatively (in total 35 lymph nodes were retrieved during surgery including sampling of FDG-avid contralateral hilar and paratracheal lymph nodes). Contralateral lymph node metastases were ruled out by both modalities.

a

Detection of T lymphocyte subsets in the peripheral blood

| Specificity | Source | Clone |
|---|---|---|
| CD3 | Beckman Coulter | A07748 |
| CD4 | BioLegend | OKT4 |
| CD8 | BioLegend | SK1 |
| Granzyme B | BioLegend | QA18A28 |

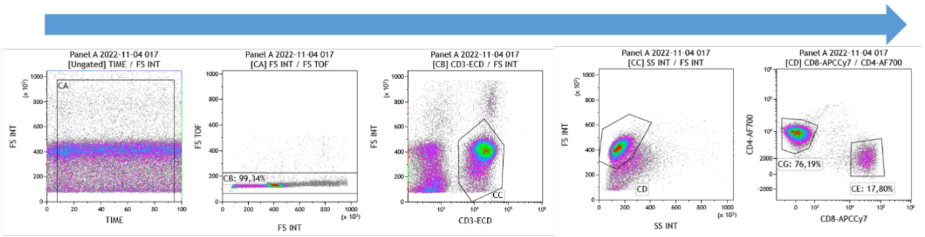


b

Detection of T lymphocyte subsets in resected tumors

| Specificity | Fluorochromes | Source | Clone |
|---|---|---|---|
| CD3 | AF790 | BioLegend | SK7 |
| CD4 | PerCP/Cy 5.5 | BioLegend | RPA-T4 |
| CD196 | BV650 | BioLegend | G034E3 |
| CD39 | BV605 | BioLegend | A1 |
| CD25 | BV421 | BioLegend | BC96 |
| CD127 | APC(IL-7Ra) | BioLegend | A019D5 |
| CD8a | BV510 | BioLegend | RPA-T8 |
| CD183 | BV785 | BioLegend | G825H7 |
| CD194 | PE/Dazzle594 | BioLegend | L291H4 |

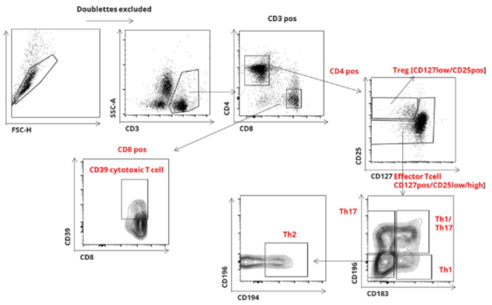


c

Detection of myeloid immune cell subsets in resected tumors

| Specificity | Fluorochromes | Source | Clone |
|---|---|---|---|
| CD11c | BV650 | BioLegend | 3.9 |
| HLA-DR | BV421 | BioLegend | L243 |
| CD4 | Per CP/Cy 5.5 | BioLegend | RPA-T4 |
| CD3 | AF700 | BioLegend | UCHT1 |
| CD8 | BV510 | BioLegend | SK1 |
| CD66b | PE | BioLegend | 6/40C |
| CD19 | PE/Cy 7 | BioLegend | HIB19 |
| CD24 | APC | BioLegend | ML5 |
| CD206 | AF488 | BioLegend | 15-2 |
| CD123 | PE/Cy5 | BioLegend | 6H6 |
| CD56 | PE/Dazzle | BioLegend | HCD56 |
| CD16 | APC/Fire750 | BioLegend | 3G8 |
| CD14 | BV785 | BioLegend | M5E2 |
| CD45 | AF488 | BioLegend | 2D1 |

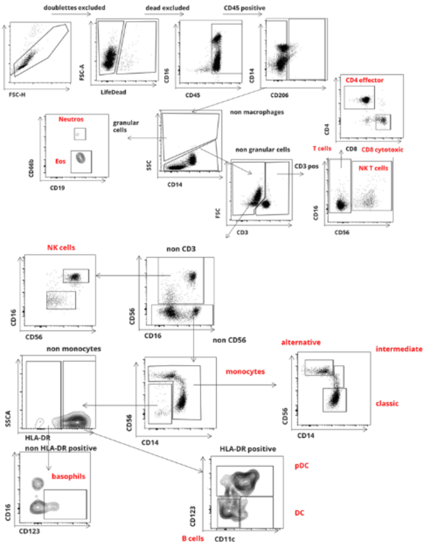


**Suppl. Fig. 2 | Primary antibody panels and gating strategies for immune cell phenotyping.**

**a**, Primary antibodies and gating strategy for detection of T lymphocyte subsets in the peripheral blood.

**b**, Primary antibodies and gating strategy for detection of T lymphocyte subsets in single cell suspensions generated from resected tumors.

**c**, Primary antibodies and gating strategy for detection of myeloid cell subsets in single cell suspensions generated from resected tumors.

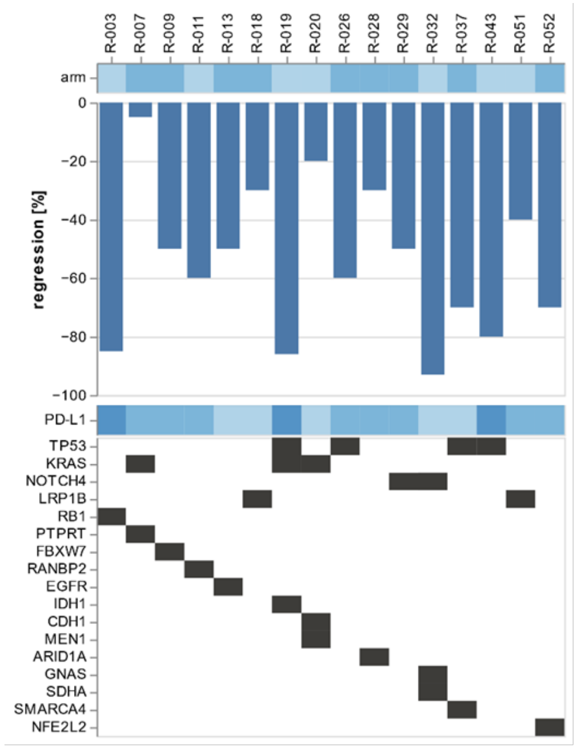


| Patient | Gene Symbol | Impact /Revel Score / ClinVar | Hgvsp | consequence | exon number / total | prob somatic normal | prob resection no change |
|---|---|---|---|---|---|---|---|
| R-003 | RB1 | HIGH | p.Ser567Ter | stop_gained | 18 / 27 | 3.31e-3 | 4.65e-8 |
| R-007 | KRAS | pathogenic | p.Gly12Cys | missense variant | 2/5 | 2.81e-3 | 7.69e-13 |
| | PTPRT | 0.73 | p.Pro193Leu | missense variant | 5/31 | 0.05 | 0.0001 |
| R-009 | FBXW7 | 0.74 | p.Gly477Ser | missense variant | 12/14 | 4.22e-8 | 4.55e-3 |
| R-011 | RANBP2 | 0.75 | p.Gln927Ter | missense variant | 20/29 | 0.03 | 4.14e-17 |
| R-013 | EGFR | pathogenic | p.Glu746_Ala750del | pathogenic variant | 19/28 | 5.36e-3 | 2.62e-5 |
| R-018 | LRP1B | 0.95 | p.Asp2835Tyr | missense variant | 53/91 | 0.03 | 0.21 |
| R-019 | TP53 | HIGH | p.Arg213Ter | stop_gained | 6/11 | 1.88e-3 | 8.87e-10 |
| | KRAS | pathogenic | p.Gly12Cys | missense variant | 2/5 | 9.92e-4 | 3.50e-13 |
| | IDH1 | 0.83 | p.Arg132Leu | missense variant | 4/10 | 1.53e-3 | 4.38e-14 |
| R-020 | KRAS | 0.88 | p.Gly12Asp | missense variant | 2/5 | 2.50e-3 | 1.28e-7 |
| | CDH1 | 0.79 | p.Gly760Ala | missense variant | 14/16 | 1.16e-6 | 2.26e-8 |
| | MEN1 | 0.95 | p.Asp252Tyr | missense variant | 4/10 | 2.66e-3 | 1.16e-18 |
| R-026 | TP53 | HIGH | p.Glu298Ter | stop_gained | 8/11 | 4.00e-3 | 3.25e-3 |
| R-028 | ARID1A | HIGH | p.Gln1519ArgfsTer8 | frameshift_variant | 18/20 | 1.96e-3 | 0.03 |
| R-029 | NOTCH4 | 0.72 | p.Gly835Val | missense variant | 16/30 | 4.44e-13 | 5.36e-3 |
| | GNAS | 0.94 | p.Arg201Cys | missense variant | 8/13 | 3.72e-3 | 5.44e-5 |
| R-032 | NOTCH4 | HIGH | p.Asp680AlafsTer13 | frameshift_variant | 13/30 | 3.16e-3 | 1.53e-12 |
| | SDHA | 0.82 | p.Thr267Met | missense variant | 7/15 | 2.86e-3 | 2.65e-7 |
| R-037 | TP53 | 0.93 | p.Arg248Gly | missense variant | 7/11 | 0.01 | n/a |
| R-043 | TP53 | 0.71 | p.Arg337Cys | missense variant | 10/11 | 1.62e-3 | 6.16e-3 |
| R-051 | LRP1B | HIGH | p.Glu2836Ter | stop_gaines | 53/91 | 3.99e-3 | 2.73e-11 |
| R-052 | NFE2L2 | pathogenic | p.Thr80Lys | missense variant | 2/5 | 3.87e-3 | 3.40e-10 |

**Suppl. Fig. 3 | Patients with significantly increased variant allele frequencies of mutated cancer genes in resected tumors compared to diagnostic biopsies.**
Left: Waterfall plot of pathologic response (% regression of viable tumor cells) in tumors and lymph nodes from patients resected following neoadjuvant study treatment (arm A light blue, arm B dark blue). The category of PD-L1 expression by tumors cells (Tumor Proportional Score, TPS <1% light blue, TPS 1 to 49% medium blue color, TPS 50 to 100% dark blue) for each patient is represented above the oncograms. The lower panel is an oncogram of cancer genes with increased variant allele frequencies following neoadjuvant immunotherapy per patient. Boxes represent pathogenic genomic aberrations in the respective gene. Right: List and details of mutated cancer genes per patient, which exhibited increased variant allele frequencies following neoadjuvant immunotherapy as compared to diagnostic pretherapeutic biopsies.

# Neoadjuvant nivolumab combination treatment in resectable non-small cell lung cancer patients: Defining optimal combinations and determinants of immunological response

**(NEOpredict-Lung)**

| | |
|---|---|
| **STUDY DRUG(s):** | Nivolumab, Relatlimab |
| **SHORT TITLE:** | NEOpredict-Lung |
| **PROTOCOL CODE:** | CA224-063 |
| **EudraCT NUMBER:** | 2019-002478-29 |
| **VERSION:** | 3.1 |
| **DATE:** | 15.12.2020 |

The information contained in this document is regarded as confidential and, except to the extent necessary to obtain informed consent, may not be disclosed to another party unless law or regulations require such disclosure. Persons to whom the information is disclosed must be informed that the information is confidential and may not be further disclosed by them.

# I. Signatures

## SPONSOR SIGNATURE PAGE

| | |
|---|---|
| **Sponsor:** | University Hospital Essen; Faculty of Medicine on behalf of the University of Duisburg-Essen, represented by Thorsten Kaatze<br><br>University Hospital Essen<br>Hufelandstrasse 55,<br>45147 Essen<br>Germany<br><br>______________________________ __________<br>Signature of Sponsor’s Representative  Date<br><br>Thorsten Kaatze<br>______________________________<br>Printed Name of Representative<br><br>By my signature, I agree to supervise the conduct of this study and to ensure its conduct in compliance with the protocol, informed consent, IRB/EC procedures, the Declaration of Helsinki, ICH Good Clinical Practices guidelines or local regulations governing the conduct of clinical studies. |

# COORDINATING INVESTIGATOR SIGNATURE PAGE
# - GLOBAL LEAD AND NATIONAL COORDINATOR GERMANY -

| | |
|---|---|
| **Global Lead and Coordinating Investigator Germany:** | Professor Dr. med. Martin Schuler<br>Department of Medical Oncology<br>West German Cancer Center<br>University Hospital Essen<br>Hufelandstrasse 55<br>45147 Essen, Germany<br>Tel.: +49 (0)201-723 2000<br>Fax: +49 (0)201-723 5924<br>martin.schuler@uk-essen.de<br><br>__________________________ __________<br>Signature of Coordinating Investigator Date<br><br>Prof. Dr. med. Martin Schuler<br>__________________________<br>Printed Name of Investigator<br><br>By my signature, I agree to conduct this study according to this protocol and to make no additions or changes without the consent of the sponsor. In addition, we agree that the study will be carried out in accordance with Good Clinical Practice (GCP), with the Declaration of Helsinki and with the laws and regulations of the country in which the study takes place. |

## SIGNATURE PAGE AUTHORS

**Authors:**

| | |
|---|---|
| Professor Dr. med. Martin Schuler<br>West German Cancer Center<br>Department of Medical Oncology<br>University Hospital Essen<br>Hufelandstrasse 55<br>45147 Essen, Germany | Date |
| Professor Dr. med. Clemens Aigner<br>West German Cancer Center<br>Department of Thoracic Surgery<br>University Medicine Essen - Ruhrlandklinik<br>Tüschener Weg 40<br>45239 Essen, Germany | Date |

**Statistician**:

| | |
|---|---|
| Dr. Christine Windemuth-Kieselbach<br>Head of Biometry<br>Alcedis GmbH<br>Winchesterstr. 3<br>35394 Gießen, Germany | Date |

## II. Synopsis

| | |
|---|---|
| Sponsor | University Hospital Essen<br>Hufelandstrasse 55<br>45147 Essen, Germany |
| Coordinating Investigator(s) | Professor Dr. med. Martin Schuler<br>West German Cancer Center<br>Department of Medical Oncology<br>University Hospital Essen<br>Hufelandstrasse 55<br>45147 Essen, Germany<br>Tel.: +49 (0) 201-723 2000<br>Fax: +49 (0) 201-723 5924<br>martin.schuler@uk-essen.de<br><br>Professor Dr. med. Clemens Aigner<br>West German Cancer Center<br>Department of Thoracic Surgery<br>University Medicine Essen - Ruhrlandklinik<br>Tüschener Weg 40<br>45239 Essen, Germany<br>Tel.: +49 (0) 201-433 4011<br>Fax: +49 (0) 201-433 4019<br>clemens.aigner@rlk.uk-essen.de |
| Title | Neoadjuvant nivolumab combination treatment in resectable non-small cell lung cancer patients: Defining optimal combinations and determinants of immunological response (NEOpredict-Lung) |
| Short title | **NEOpredict-Lung** |
| Indication | Non-small cell lung cancer stages I B, II and selected stages III A |
| Phase | Phase II |
| Study design | Multicenter, open-label, randomized, two-armed trial |

| | |
|---|---|
| Participating centers | West German Cancer Center<br>University Hospital Essen<br>Essen, Germany<br><br>Netherlands Cancer Institute<br>Antoni van Leeuwenhoek<br>Amsterdam, The Netherlands<br><br>Jessa Hospital Hasselt<br>Hasselt, Belgium |
| Number of subjects | A maximum of 30 evaluable patients will be enrolled per treatment arm. Patients will be randomized (1:1) to arm A (nivolumab) or arm B (nivolumab plus relatlimab). |

| | |
|---|---|
| Study objective(s) | **Primary objective**<br><br>The primary objective of this study is to determine the feasibility of four weeks of preoperative immunotherapy with nivolumab, and nivolumab plus relatlimab in patients with early stage or locally advanced non-small cell lung cancer eligible for curative resection.<br><br>Based on the results of a pilot study[1] it is expected that at least 26 of 30 patients treated in each study arm will undergo curatively intended surgery within 6 weeks of initiation of study treatment. A study arm will be declared non-feasible if 5 or more patients experience a delay of curatively intended surgery beyond day 43 (with study treatment being administered on day 1), either due to toxicities or disease progression.<br><br>**Secondary objectives**<br><br>• Estimation of pathological tumor response rate (rate of complete pathological responses defined as absence of viable tumor cells on routine hematoxylin and eosin staining of resected tumors and lymph nodes; rate of major pathological responses defined as 10% or less viable tumor cells on routine hematoxylin and eosin staining of resected tumors)<br>• Estimation of curative (R0) resection rate<br>• Assessment of radiologic response on computed tomography per Response evaluation criteria in solid tumors (RECIST) version 1.1<br>• Assessment of disease-free survival rate at 12 months per Response Evaluation Criteria In Solid Tumors (RECIST) version 1.1<br>• Assessment of overall survival rate at 12 months<br>• Assessment of safety and tolerability of preoperative immunotherapy<br>• Estimation of morbidity and mortality within 90 days of surgery<br>• Exploratory translational analyses for investigation of immunomodulatory and anticancer activity of preoperative treatment with nivolumab and nivolumab/relatlimab combination therapy |

<table>
<tr><td>Study end points</td><td>Primary variable<br><br>Primary variable is the number of patients undergoing curatively intended surgery of non-small cell lung cancer within 43 days of initiation of study therapy.<br><br>Secondary variables<ul><li>Objective response rate (RECIST 1.1)</li><li>Pathological response rate (complete pathological responses defined as absence of viable tumor cells on routine hematoxylin and eosin staining of resected tumors and lymph nodes; rate of major pathological responses defined as 10% or less viable tumor cells on routine hematoxylin and eosin staining of resected tumors)</li><li>R0 resection rate</li><li>Radiomorphologic response per RECIST 1.1</li><li>Disease-free survival rate at 12 months per RECIST 1.1</li><li>Overall survival rate at 12 months</li><li>Safety and tolerability of preoperative immunotherapy</li><li>Morbidity and mortality within 90 days of curative surgery</li></ul>Exploratory variables<ul><li>Translational parameters are assessed in tumor and lymph node samples, blood cells, plasma, serum, and exhalate (for details see NEOpredict-Lung Translational Protocol)</li></ul></td></tr>
<tr><td>Medical condition</td><td>Patients aged ≥ 18 years with localized or locally advanced non-small cell lung cancer stages I B, II or selected III A eligible for curatively intended surgery</td></tr>
</table>

| | |
|---|---|
| Eligibility criteria | **Principal inclusion criteria**<br>• Patients with histologically (core biopsy) or cytologically (e.g. bronchoscopy-guided biopsy) confirmed non-small cell lung cancer (NSCLC) eligible for anatomic resection, with the following specifications:<br>  o Clinical stages I B, II and selected stage III A (T3 N1, T4 with satellite nodule in the same lung N0/N1, selected T1a-T2b N2 cases considered suitable for primary surgical approach by the multidisciplinary tumor board) according to UICC 8$^{th}$ edition<br>• Males and females, ages ≥ 18 years, inclusive<br>  o Women of childbearing potential (WOCBP) must have a negative serum or urine pregnancy test (minimum sensitivity 25 IU/L or equivalent units of human chorionic gonadotropin [hCG]) within 24 hours prior to the start of study treatment.<br>  o Women of childbearing potential (WOCBP) must agree to follow instructions for highly effective method(s) of contraception for the duration of treatment with study medication plus 5 half-lives of study treatment plus 30 days (duration of ovulatory cycle) post treatment completion for a total of 165 days (approximately 24 weeks) (Appendix IV). This applies to both treatment arms, nivolumab monotherapy and nivolumab/relatlimab combination therapy.<br>  o Males who are sexually active with WOCBP must agree to follow instructions for method(s) of contraception and fetal protection (Appendix IV) for the duration of treatment with study medication plus 5 half-lives of the study treatment plus 90 days (duration of sperm turnover) for a total of 225 days (approximately 33 weeks) post-treatment completion. In addition, male participants must be willing to refrain from sperm donation during this time. This applies to both treatment arms, nivolumab monotherapy and nivolumab/relatlimab combination therapy.<br>• ECOG ≤ 1<br>• Exclusion of extensive mediastinal lymph node metastases (multilevel N2, N3) by PET/CT and/or invasive mediastinal lymph node staging by EBUS-TBNA and/or staging mediastinoscopy as indicated by current guidelines.<br>• Exclusion of distant metastases by standard of care imaging studies, which include but are not limited to PET/CT or PET/MRI, or CT or MRI |

| | |
|---|---|
| | of thorax, abdomen, pelvis, and bone scan. Asymptomatic brain metastases will be excluded by MRI or contrast-enhanced CT as indicated by current guidelines.<br>• Measurable target tumor prior to immunotherapy using standard imaging techniques.<br>• Sufficient pulmonary function to undergo curative lung cancer surgery, ppFEV1>30%, ppDLCO>30%, ppVO2max ≥ 10 ml/min/kg (if CPET was mandated per local guidelines)<br>• Adequate hematological, hepatic and renal function parameters:<br>  o Leukocytes ≥ 2,000/mm³, platelets ≥ 100,000/mm³, absolute neutrophil count (ANC) ≥ 1,500/µL, hemoglobin ≥ 9 g/dL (5.58 mmol/L),<br>  o Anti-platelet therapy (such as but not limited to clopidogrel) should be discontinued pre-operatively according to local standards. If this therapy cannot be interrupted due to severe cardiovascular comorbidity, patient is ineligible for the trial<br>  o Adequate coagulation function as defined by International Normalized Ratio (INR) ≤ 1.5, and a partial thromboplastin time (PTT) ≤ 5 seconds above the upper limit of normal (ULN) (unless receiving anticoagulation therapy). Patients receiving warfarin/phenprocoumon or direct oral anticoagulants are to be bridged according to local standards and have achieved stable coagulation profile prior to surgery.<br>  o Serum creatinine ≤ 1.5 x upper limit of normal<br>  o Bilirubin ≤ 1.5 x upper limit of normal, AST and ALT ≤ 3.0 x upper limit of normal, alkaline phosphatase ≤ 6 x upper limit of normal<br>• Sufficient cardiac left ventricular defined as LVEF ≥ 50% documented either by echocardiography or MUGA (echocardiography preferred test, MUGA not used in German site) within 6 months before first administration of study drug<br>• Patient able and willing to provide written informed consent and to comply with the study protocol and with the planned surgical procedures |

| | |
|---|---|
| Eligibility criteria | **Principal exclusion criteria**<br><br>• Active or history of autoimmune disease or immune deficiency, including, but not limited to, myasthenia gravis, myositis, autoimmune hepatitis, systemic lupus erythematosus, rheumatoid arthritis, inflammatory bowel disease, anti-phospholipid antibody syndrome, Wegener granulomatosis, Sjögren syndrome, Guillain-Barré syndrome, multiple sclerosis, vasculitis, or glomerulonephritis.<br><br>Patients with the following conditions are **not excluded** from participation:<br>o Patients with a history of autoimmune-related hypothyroidism who are on thyroid-replacement hormone are eligible for the study.<br>o Patients with controlled Type 1 diabetes mellitus who are on an insulin regimen are eligible for the study.<br>o Skin disorders (such as vitiligo, psoriasis, or alopecia) not requiring systemic immunosuppressive treatment, in particular corticosteroids are permitted to enroll.<br><br>• Subjects with a condition requiring systemic treatment with either corticosteroids (> 10 mg daily prednisone equivalents) or other immunosuppressive medications within 14 days of study drug administration. Inhaled or topical steroids, and adrenal replacement doses > 10 mg daily prednisone equivalents are permitted in the absence of active autoimmune disease.<br>• Subjects who have undergone organ transplant or allogeneic stem cell transplantation.<br>• ppFEV1<30%, ppDLCO<30%, ppVO2max < 10 ml/min/kg (if CPET was mandated per local guidelines)<br>• Uncontrolled or significant cardiovascular disease including, but not limited to, any of the following:<br>o Myocardial infarction (MI) or stroke/transient ischemic attack (TIA) within the 6 months prior to consent<br>o Uncontrolled angina within the 3 months prior to consent<br>o Any history of clinically significant arrhythmias (such as ventricular tachycardia, ventricular fibrillation, or torsades de pointes)<br>o QTc prolongation > 480 msec<br>o Pulmonary hypertension (sPAP >35 mmHg)<br>• History of other clinically significant cardiovascular disease (i.e., cardiomyopathy, congestive heart failure with New York Heart Association [NYHA] functional classification III-IV, pericarditis, |

| | |
|---|---|
| | significant pericardial effusion, significant coronary stent occlusion, deep venous thrombosis, etc. )<br>• Cardiovascular disease-related requirement for daily supplemental oxygen<br>• History of two or more myocardial infarctions or two or more coronary revascularization procedures<br>• Subjects with history of myocarditis, regardless of etiology<br>• Troponin T (TnT) or I (TnI) > 2 × institutional upper limit of normal (ULN). Subjects with TnT or TnI levels between > 1 to 2 × ULN will be permitted if repeat levels within 24 hours are within ULN. If TnT or TnI levels are >1 to 2 × ULN within 24 hours, the subject may undergo a cardiac evaluation and be considered for treatment, following a discussion with the coordinating investigator or designee. When repeat levels within 24 hours are not available, a repeat test should be conducted as soon as possible. If TnT or TnI repeat levels beyond 24 hours are < 2 x ULN, the subject may undergo a cardiac evaluation and be considered for treatment, following a discussion with the coordinating investigator or designee<br>• Patients with active neurological disease should be excluded.<br>• Active malignancy or a prior malignancy within the past 3 years. Patients with the following conditions are **not excluded** from participation:<br>    o Patients with completely resected basal cell carcinoma, cutaneous squamous cell carcinoma, cervical carcinoma in-situ, breast carcinoma in-situ, and patients with isolated elevation in prostate-specific antigen or low risk prostate cancer managed with active surveillance or watchful waiting in the absence of radiographic evidence of metastatic prostate cancer.<br>• Known history of positive test for human immunodeficiency virus (HIV-1 and HIV-2) or known acquired immunodeficiency syndrome (AIDS). NOTE: Testing for HIV-1 and HIV-2 must be performed at screening.<br>• Any positive test result for hepatitis B virus or hepatitis C virus indicating presence of virus, e.g., hepatitis B surface antigen (HBsAg, Australia antigen) positive, or hepatitis C antibody (anti-HCV) positive (except if HCV RNA negative).<br>• Any other disease, metabolic dysfunction, physical examination finding, or clinical laboratory finding that contraindicates the use of an investigational drug, may affect the interpretation of the results, or may render the patient at high risk from treatment complications<br>• Receipt of live attenuated vaccine within 30 days prior to the first dose of study medication |

| | |
|---|---|
| | • Peripheral polyneuropathy NCI CTCAE Grade ≥ 2<br>• History of gastric perforation or fistulae in past 6 months<br>• Serious or non-healing wound, ulcer, or bone fracture within 28 days prior to enrollment.<br>• The patient has undergone major surgery within 28 days prior to enrollment except staging mediastinoscopy, diagnostic VATS or implantation of a venous port-system.<br>• Any other concurrent preoperative antineoplastic treatment including irradiation<br>• Pregnant women<br>• Breastfeeding women<br>• Insufficient cardiac left ventricular function defined as LVEF<50% by echocardiography (outside Germany: or MUGA scan)<br>• A confirmed history of encephalitis, meningitis, or uncontrolled seizures in the year prior to informed consent<br>• Subjects with history of severe toxicity or life-threatening toxicity (grade 3 or 4) related to prior immune therapy (e.g. anti-CTLA-4 or anti-PD-1/PD-L1 treatment or any other antibody or drug specifically targeting T-cell co-stimulation or immune checkpoint pathways) except those that are unlikely to re-occur with standard countermeasures (e.g., hormone replacement after endocrinopathy).<br>• Subjects with history of severe or life-threatining (grade 3 or 4) infusion-related reactions to prior immune therapy<br>• Prior treatment with LAG-3 targeting agent<br><br>**Other**<br>• Participation in another interventional clinical study within the last 3 months prior to inclusion or simultaneous participation in other clinical studies<br>• Previous treatment with nivolumab or relatlimab<br>• Previous immunotherapy for lung cancer<br>• Criteria which in the opinion of the investigator preclude participation for scientific reasons, for reasons of compliance, or for reasons of the subject's safety<br>• Any contraindications against nivolumab or relatlimab |

<table>
<tr><td>Study treatment(s)</td><td>

**Arm A**: Nivolumab 2 cycles, every two weeks (q2w)

- nivolumab 240 mg i.v. over 30 min

**Arm B**: Nivolumab/Relatlimab 2 cycles, every two weeks (q2w)

- nivolumab 240 mg i.v. over 30 min
- relatlimab 80 mg i.v. over 30 min (within 30 min of nivolumab)

</td></tr>
<tr><td>Statistical methods</td><td>

The objective of this study is to determine the feasibility of two cycles of preoperative immunotherapy in patients eligible for curative anatomic resection of NSCLC. Sequential boundaries will be used to monitor the non-feasibility rate. The accrual for each study arm will be halted if excessive numbers of delays of curative surgery beyond 6 weeks from initiation of study treatment are seen, that is, if the number of delayed surgeries is equal to or exceeds $b_n$ out of $n$ patients with sufficient follow-up (see table). This is a Pocock-type stopping boundary that yields the probability of crossing the boundary at most 0.05 (probability of early stopping) when the rate of delayed surgeries is equal to the acceptable rate 0.05 (event probability $\theta$).

<table>
<tr><td>n</td><td>1</td><td>2</td><td>3</td><td>4</td><td>5</td><td>6</td><td>7</td><td>8</td><td>9</td><td>10</td><td>11</td><td>12</td><td>13</td><td>14</td><td>15</td><td>16</td><td>17</td><td>18</td><td>19</td><td>20</td></tr>
<tr><td>b<sub>n</sub></td><td>-</td><td>2</td><td>2</td><td>2</td><td>3</td><td>3</td><td>3</td><td>3</td><td>3</td><td>3</td><td>3</td><td>3</td><td>4</td><td>4</td><td>4</td><td>4</td><td>4</td><td>4</td><td>4</td><td>4</td></tr>
<tr><td>n</td><td>21</td><td>22</td><td>23</td><td>24</td><td>25</td><td>26</td><td>27</td><td>28</td><td>29</td><td>30</td></tr>
<tr><td>b<sub>n</sub></td><td>4</td><td>4</td><td>5</td><td>5</td><td>5</td><td>5</td><td>5</td><td>5</td><td>5</td><td>5</td></tr>
</table>

This boundary is equivalent to testing the null hypothesis after each patient, that the event rate is equal to 0.05, using a one-sided level 0.022387 test.

All secondary parameters will be evaluated in an explorative or descriptive manner, providing means, medians, ranges, standard deviations and/or confidence intervals. All p-values will be two-sided if not stated otherwise.

The amount and subsets of tumor infiltrating lymphocytes and peripheral immune cells before and after therapy will be analyzed using t-test, Mann-Whitney-U test or ANOVA. Overall response rate (ORR), pathological response rates (complete pRR and major pRR), toxicity and other event rates in each arm will be compared using Fishers exact test and $\chi^2$ test; odds ratios with confidence intervals and p-value will be provided.

DFS and OS will be analyzed using Kaplan-Meier methods and compared by log rank test. Univariate and multivariate analyses will eventually be performed by suitable regression models (logistic regression, proportional hazard regression model).

</td></tr>
<tr><td>Time plan</td><td>

**For the individual patient**

</td></tr>
</table>

| | |
|---|---|
| | Approximately 13 months (treatment: 4 weeks, preoperative period: up to 2 weeks, postoperative follow-up: 12 months)<br>**Planned study schedule**<br>First Patient In Q1/2020<br>Last Patient In Q3/2022<br>Last Patient EoT Q4/2022<br>Last Follow-Up Q4/2023<br>DBL Q3/2024<br>End of Study Q3/2024 |
| Financing | This is an investigator-initiated, international, multicenter study, which receives financial support by a research grant from Bristol–Myers Squibb |

# 1 Table of contents

I. Signatures .....2
II. Synopsis .....5
1 Table of contents .....16
II.a) List of tables .....21
II.b) List of figures .....21
III. Abbreviations .....22
2 Scientific background and rationale .....24
2.1 Introduction .....24
2.2 Rationale of the study .....24
2.2.1 Rationale and previous studies of nivolumab in non-small cell lung cancer and dose selection .....27
2.2.2 Rationale for combining nivolumab with relatlimab, dose selection and preliminary clinical data .....28
2.2.3 Mechanism of Action .....28
2.2.4 Summary of Clinical Pharmacology .....30
2.2.5 Safety Summary .....31
2.3 Risk benefit assessment .....32
3 Objective of the study .....34
3.1 Primary objective .....34
3.2 Secondary and other objectives .....34
4 Organizational and administrative aspects of the study .....36
4.1 Sponsor .....36
4.2 Statistics .....36
4.3 Investigators and study sites .....37
4.4 Financing .....38
5 Study design .....39
5.1 Design overview .....39
5.2 Study variables .....40
5.3 Primary variable .....40
5.3.1 Secondary variables .....40
5.3.2 Translational analyses .....40

5.4 Justification of study design ..... 41
5.5 End of the clinical study ..... 42
6 Study population ..... 43
6.1 Justification of selection criteria ..... 43
6.2 Justification of gender selection ..... 43
6.3 Inclusion criteria ..... 43
6.4 Exclusion criteria ..... 45
6.5 Withdrawal and replacement of study subjects ..... 47
6.6 Replacement ..... 48
6.7 Subject identification ..... 48
7 Treatment / Investigational drug ..... 48
7.1 Treatments to be given ..... 48
7.2 Description of the Investigational Medicinal Products (IMPs) ..... 49
7.2.1 Test drug ..... 49
7.2.2 Labelling of Investigational Medicinal Product (IMP) ..... 50
7.3 Storage conditions ..... 50
7.4 Drug logistics and accountability ..... 51
7.5 Assignment to treatment group ..... 51
7.6 Blinding ..... 51
7.7 Study drug administration ..... 51
7.7.1 Preparation and administration of nivolumab and relatlimab ..... 51
7.8 Dose modification guidelines ..... 52
7.8.1 Dose modification guidelines for nivolumab or nivolumab and relatlimab therapy ... 52
7.9 Previous and concomitant treatment ..... 57
7.10 Post-study therapy ..... 59
8 Procedures and variables ..... 59
8.1 Tabulated schedules ..... 59
8.1.1 Translational studies ..... 66
8.2 Description of visits ..... 66
8.2.1 Screening (Days -28 to Day 0) ..... 66
8.2.2 Allocation to treatment ..... 68
8.2.3 Treatment visits W1 and W3 ..... 68

8.2.4 Visit pre-OP ..... 68
8.2.5 Visit post-OP (D1 to 7 after surgery) ..... 69
8.2.6 Follow up visits (1, 2 and 3 months after surgery and every 3 months thereafter for up to 12 months after surgery) (± 5 days) ..... 70
8.2.7 End of study visit ..... 70
8.3 Laboratory assessments ..... 71
8.4 Demographic characteristics ..... 71
8.5 Evaluation of clinical endpoints ..... 72
8.6 Safety examinations ..... 72
9 Response criteria ..... 74
10 Safety ..... 74
10.1 Definitions of adverse events and adverse drug reactions ..... 74
10.1.1 Classification of adverse events ..... 74
10.1.2 Serious adverse events (SAEs) ..... 74
10.1.3 Adverse drug reactions and serious adverse drug reactions ..... 75
10.1.4 Unexpected adverse drug reaction ..... 75
10.1.5 SUSAR - Suspected unexpected serious adverse reactions ..... 76
10.1.6 Events of special interest ..... 76
10.2 Documentation and follow-up of adverse events and serious adverse events ..... 77
10.2.1 SAE documentation responsibilities ..... 78
10.2.2 Collection and Reporting of AE/SAEs ..... 79
10.2.3 Events not to be treated as AEs ..... 79
10.2.4 Events not to be treated as SAEs ..... 80
10.3 Contact information for safety reporting ..... 81
10.4 Investigator reporting responsibilities. ..... 81
10.5 Notification of SAEs to the EC/IRB ..... 81
10.6 Notification of the competent authority ..... 81
10.7 Notification of the manufacturer ..... 82
10.8 Sponsors reporting responsibilities ..... 82
10.9 Development Safety Update Report (DSUR) ..... 82
10.10 Expected adverse events ..... 83
10.10.1 Expected adverse events related to treatment with OPDIVO® (nivolumab) ..... 83

10.10.2 Expected adverse events related to treatment with relatlimab ..............................83
10.11 Pregnancies..........................................................................................83
10.12 Overdose...............................................................................................85
10.13 Data monitoring committee ...................................................................85
11 Statistical methods and sample size calculation ......................................85
11.1 General statistical considerations ...........................................................85
11.2 Analysis sets...........................................................................................86
11.3 Sample size ............................................................................................86
11.4 Randomization / Stratification ................................................................87
11.5 Variables and planned statistical analyses.............................................87
11.5.1 Variables ..............................................................................................87
11.5.2 Statistical methods................................................................................87
11.6 Subgroup analyses.................................................................................88
11.7 Interim analysis.......................................................................................88
11.8 Analysis and reporting ............................................................................88
12 Documentation .........................................................................................88
12.1 Study records requirements....................................................................89
12.2 Data management ..................................................................................89
12.3 Archiving.................................................................................................89
12.4 Monitoring...............................................................................................90
12.5 Audits/Inspections...................................................................................91
13 Ethical and regulatory aspects..................................................................91
13.1 Independent ethics committee ................................................................91
13.2 Notification of the authorities, approval and registration.........................92
13.3 Ethical basis for the clinical study ...........................................................92
13.4 Obtaining informed consent from study subjects.....................................92
13.5 Protocol deviations .................................................................................93
13.6 Compensation for health damage of subjects ........................................93
13.7 Confidentiality.........................................................................................93
14 Closure of study sites/Premature termination of the clinical study..........94
15 Publication...............................................................................................94
16 Amendments to the study protocol...........................................................95

17 References ........................................................................................ 96
18 Appendices ........................................................................................ 99
18.1 Appendix I: CTCAE Version 4.03 ........................................................ 99
18.2 Appendix II: ECOG-Performance Status Scale ....................................... 99
18.3 Appendix III: Revised RECIST response criteria (V1.1) .......................... 99
18.3.1 Evaluation of target lesions ............................................................ 100
18.3.2 Evaluation of non-target lesions ....................................................... 100
18.3.3 Evaluation of best overall response .................................................. 100
18.4 Appedix IV: Contraception Guidelines .................................................. 101
18.4.1 Women of childbearing potential definitions and methods of contraception .......... 101
18.4.2 Contraception guidance for female participants of child bearing potential ............ 101
18.4.3 Contraception guidance for male participants with partner(s) of child bearing potential ........................................................................................ 103
18.4.4 Collection of pregnancy information .................................................. 104
18.5 APPENDIX V: Medications Associated with QT Prolongation ........................ 104

## II.a) List of tables

Table 1: Treatments ....................................................................................................48

Table 2: Test drug .......................................................................................................50

Table 3: Permitted and prohibited concomitant therapy (All study arms) ..............................58

Table 4: Schedule of assessments.....................................................................................59

Table 5: Laboratory parameters .......................................................................................71

Table 6: Overview of AE reporting requirements .................................................................78

## II.b) List of figures

Figure 1: Study design NEOpredict-Lung ...............................................................................40

## III. Abbreviations

| | |
|---|---|
| ADR | Adverse drug reaction |
| AE | Adverse Event |
| AEG | Adenocarcinoma of the esophago-gastric junction |
| ALT | Alanine aminotransferase |
| AMG | Arzneimittelgesetz *(German Drug Law)* |
| ANC | Absolute neutrophil count |
| AST | Aspartate aminotransferase |
| BMI | Body Mass Index |
| BSC | Best supportive care |
| CIAC/CEAC | Central independent adjudication endpoint committee |
| CPI | Coordinating Principal Investigator (*Leiter der klinischen Prüfung*) |
| CT | Computer tomography |
| CTCAE | Common Terminology Criteria for Adverse Events |
| DFS | Disease free survival |
| DSMB | Data and Safety Monitoring Board |
| DSUR | Drug Safety Update Report |
| EC | Ethics committee |
| ECOG | Eastern Cooperation Oncology Group |
| eCRF | Electronic case report form |
| FAS | Full analysis set |
| FDG | Flour-desoxy-glucose |
| fT 3,4 | free trijodthyronine 3, 4 |
| FU | Follow-up |
| GC | Gastric Cancer |
| GCP | Good clinical practice |
| GEJ | Estrophago-gastric junction |
| GFR | Glomerular filtration rate |
| ICF | Informed consent form |
| ICI | Immune Checkpoint Inhibitor |
| ICMJE | International Committee of Medical Journal Editors |

| | |
|---|---|
| IEC | Independent ethics committee |
| IMP | Investigational medicinal product |
| INR | International normalized ratio |
| ISF | Investigator site file |
| ITT | Intent-to-treat |
| i.v. | Intravenous |
| LPLV | Last patient last visit |
| MTD | Maximum tolerated dose |
| NSCLC | Non-small cell lung cancer |
| ORR | Overall response rate |
| PET | Positron emission tomography |
| PP | Per protocol |
| (P)PK | (Population) Pharmocokinetics |
| PTT | Partial thromboplastin time |
| q2w | Every two weeks |
| qd | Every day |
| QoL | Quality of life |
| RCC | Renal cell cancer |
| RECIST | Response evaluation criteria in solid tumors |
| SADR | Serious adverse drug reaction |
| SAE | Serious adverse event |
| SAP | Statistical Analysis Plan |
| SAR | Suspected adverse reaction |
| SmPC | Summary of Product Characteristics |
| SUSAR | Suspected unexpected serious adverse reaction |
| TMB | Tumor mutational burden |
| TMF | Trial master file |
| TSH | Thyroid-stimulating hormone |
| ULN | Upper limit of normal |
| WOCBP | Women of child-bearing potential |

# 2 Scientific background and rationale

## 2.1 Introduction

NEOpredict-Lung is a phase II, randomized, two-arm, clinical study in subjects with histologically or cytologically confirmed, early stage or locally advanced non-small cell lung cancer (NSCLC) eligible for curative resection. This study will determine the feasibility of two cycles of preoperative immunotherapy with nivolumab or nivolumab plus relatlimab. Primary endpoint is the number of patients experiencing a delay of curatively intended surgery beyond 14 days after the end of preoperative immunotherapy; this will be separately assessed for each treatment arm. It is hypothesized that at least 26 of 30 patients per treatment arm undergo curative surgery within 6 weeks after start of preoperative immunotherapy to declare the respective treatment arm feasible. Sequential boundaries will be used to continuously monitor the non-feasibility rate. This allows early closure of treatment arms with a high likelihood of being unfeasible. Additional objectives include pathological response rates, resection rates, disease-free survival (DFS) rates and overall survival (OS) rates at 12 months, safety and tolerability of preoperative immunotherapy, and morbidity and mortality rates within 90 days of curative surgery. Furthermore, translational endpoints will be investigated.

## 2.2 Rationale of the study

Anatomic resection with systematic mediastinal lymph node dissection is the standard of care for patients with localized NSCLC who are medically and technically eligible for surgery. Long-term survival rates ranging from 92% for patients with stage I A1, to 36% in patients with stage III A after 5 years are reported by the IASLC data base. Main denominators of risk of death in resectable lung cancer include tumor size and presence of lymph node metastases. For example, a tumor size of 2 to 3 cm (pT1c) already doubles the risk of death as compared to tumors of 1 cm and below[2]. To reduce the risk of disease recurrence and improve disease-related mortality adjuvant chemotherapy is recommended in suitable patients with stage II and stage III disease. Data from large registries have even suggested a significant risk reduction by adjuvant chemotherapy in patients with tumor sizes of 3.1 to 3.9 cm, which are staged as pT2a (stage I B) according to the current staging system[3]. Adjuvant radiotherapy is suggested if N2 lymph node involvement is postoperatively documented (Leitlinienprogramm Onkologie [Deutsche Krebsgesellschaft, Deutsche Krebshilfe, AWMF]: Prävention, Diagnostik, Therapie und Nachsorge des Lungenkarzinoms, Langversion 1.0, 2018, AWMF-Registernummer: 020/007OL). The current standard of adjuvant systemic therapy consists of four cycles of cisplatin-based chemotherapy. With this an absolute increase of overall survival in the range of 8 to 15% at 5 years is reported in phase III studies[4–6]. Nevertheless, a substantial proportion of

patients relapse despite adjuvant chemotherapy and radiation. Moreover, patient populations of the pivotal studies of adjuvant chemotherapy do not fully represent today's "real life population" presenting in lung cancer centers. With recent improvements in operative therapy and perioperative management, particularly advances in minimally invasive surgery by video-assisted and robotic approaches, more elderly and comorbid NSCLC patients become eligible for curative surgery. For such patients there is limited evidence for the value of cisplatin-based adjuvant chemotherapy, which may not provide the appropriate risk-benefit balance. In summary, new systemic therapies are required to increase cure rates in patients with localized NSCLC eligible for surgery.

Recently, agents targeting regulatory mechanisms of the immune response have been introduced as a new therapeutic modality in medical oncology. Conceptionally, these so-called "immune checkpoint inhibitors" (ICI) either relieve endogenous suppression of an ongoing antitumoral immune response, or they facilitate and enhance the induction of anticancer immunity. Most prominent examples for clinically active ICI are monoclonal antibodies disrupting the communication between immune cells and antigen-presenting cells including cancer cells through the PD-1/PD-L1 and CTLA-4/CD80/CD86 receptor-ligand systems. These agents have been clinically explored in a wide range of cancer entities including NSCLC. Antibodies targeting PD-1 (nivolumab, pembrolizumab) and PD-L1 (atezolizumab, durvalumab) have shown superiority over standard chemotherapy in several pivotal phase III studies in patients with advanced/metastatic or recurrent NSCLC[7–12]. Combining the ICIs nivolumab and ipilimumab (anti-CTLA-4) demonstrated superiority over platinum-based standard chemotherapy in treatment-naïve NSCLC patients which was selected based on high tumor mutational burden[13]. More recently, superiority of nivolumab plus ipilimumab over platinum-based standard chemotherapy in first-line treatment of patients with metastatic NSCLC was reported[27].

The anticancer activity of ICI is thought to be executed through indirect mechanisms involving cytotoxic T cells that recognize cancer neoantigens presented in the appropriate MHC class I context[14]. Based on these considerations the clinical activity of ICI should not be restricted to advanced stage, metastatic cancers. Susceptibility to ICI therapy of a given cancer cell or population is thought to be determined by the presence of neoantigens, their accessibility to immune cells, and the containment of local and systemic immune evasion mechanisms. Recently, positive pivotal studies of adjuvant ICI therapy have been reported in patients with high risk localized malignant cutaneous melanoma[15,16]. Moreover, consolidation therapy with the anti-PD-L1 antibody durvalumab significantly improved progression-free survival and overall survival in patients with locally advanced NSCLC who had been treated with concurrent radiochemotherapy[12,17]. This strongly supports the efficacy of ICI therapy in preventing local and distant recurrence in curatively treated NSCLC patients.

Numerous studies in several cancer entities including breast cancer, bladder cancer, gastric cancer, rectal cancer and NSCLC have demonstrated the feasibility and clinical benefit of moving risk-reducing systemic therapies to the preoperative window. This concept of "neoadjuvant therapy" provides multiple advantages over adjuvant treatment: with measurable tumor lesions still present the individual clinical activity of the respective systemic treatment can be monitored *in vivo*. In many cases "downstaging" by preoperative treatment enables less radical, organ-preserving surgical approaches. It may also allow technical resectability at all, and

increases the likelihood of clear resection margins (R0 resection). Perioperative complications or prolonged postoperative recovery may defer or even prevent delivery of postoperative, adjuvant systemic therapy. Severall studies in patients with localized NSCLC confirmed that neoadjuvant chemotherapy is equally effective as postoperatively administered, adjuvant chemotherapy[18–21]. Recently, a small pilot study was reported which explored preoperative ICI with two courses of nivolumab in patients planned for curative resection of NSCLC. Safety, feasibility and a high rate of histopathological responses were demonstrated in a limited and selected patient population. Interestingly, radiomorphologic response did not correlate with histopathological response. Importantly, 20 of 21 eligible patients subsequently underwent complete tumor resection; in one patient the tumor was found to invade the trachea at surgery preventing a complete resection[1]. This promising early report on the use of an ICI in the neoadjuvant setting has already led to the start of follow-up studies. Additional studies of preoperative atezolizumab, pembrolizumab, nivolumab or nivolumab combined with ipilimumab are conducted in patients with early stage resectable cancer. Safety and preliminary efficacy data of such trials in NSCLC[22,23], colon cancer[24] and urothelial bladder cancer[25] were presented and confirm the feasibility of the concept of preoperative ICI therapy. Currently, many more checkpoint inhibitors have been developed for clinical application and hold promise for the future. The high number of new possible combinations would take years to decide which combination is most effectively activating cancer immunity if one would use the conventional approach in patients with stage IV disease. Early recognition of responses is key in managing this problem.

Against this background the present study NEOpredict-Lung was designed to further explore and optimize neoadjuvant ICI therapy in patients with stages I B, II or selected stage III A NSCLC eligible for curative resection. The study is designed to enable comparison of alternative strategies of neoadjuvant ICI therapy with nivolumab monotherapy used as reference. All patients will receive two 14-day-cycles of nivolumab-based ICI therapy. This is followed by standard of care curative surgery, which shall be conducted no later than 14 days after the end of the second ICI therapy cycle. Based on the pathological tumor stage, current treatment guidelines and the recommendation of the interdisciplinary lung cancer tumor board of each participating institution postoperative standard of care therapy will be offered to all patients, which is delivered outside of the NEOpredict-Lung study. The primary aim of the study is to demonstrate feasibility of each arm of preoperative ICI therapy. Towards this end the rate of patients who do not proceed to curative surgery within the predefined time frame is continuously monitored for each study arm. This allows early closure of unfeasible treatment arms based on prespecified criteria. Secondary study endpoints include tumor response (histopathological and radiological), safety of preoperative ICI therapy, and survival rates at 12 months. Translational endpoints, which are described in a separate translational study protocol, include markers of immune modulation by preoperative ICI therapy, and predictive biomarkers. The following study arms are planned:

**Arm A:** Nivolumab 240 mg q2w, 2 cycles. As there is still limited data on safety and efficacy of short-term preoperative treatment with nivolumab or similar antibodies against PD-1/PD-L1 in patients with resectable lung cancer, arm A will provide an important benchmark for indirect comparison with ongoing or completed trials at other centers and translational research [1,23]. Patients will be allocated by 1:1 randomization to Arm A and Arm B for up to 30 patients in each arm.

**Arm B:** Nivolumab 240 mg q2w plus relatlimab 80 mg q2w, 2 cycles. Relatlimab is a monoclonal antibody targeting the negative immune regulator LAG-3. Safety, recommended dose and early clinical efficacy of relatlimab in combination with nivolumab has been established in clinical trials. Currently, pivotal clinical studies of nivolumab plus relatlimab are ongoing in several entities including malignant melanoma and gastric cancer.

### 2.2.1 Rationale and previous studies of nivolumab in non-small cell lung cancer and dose selection

Nivolumab is an effective drug in NSCLC. It is globally approved for treatment of patients with metastatic or recurrent NSCLC of squamous and non-squamous histology, who have progressed after at least one line of standard chemotherapy. The randomized studies CheckMate 017 and CheckMate 057 have demonstrated superior overall survival with nivolumab monotherapy as compared to standard second-line chemotherapy with docetaxel. Most responses with nivolumab were durable[7,8]. The randomized study CheckMate 026 has compared nivolumab to platinum-based chemotherapy in previously untreated patients with stage IV NSCLC. Patients were selected based on expression of PD-L1 in at least 5% of tumor cells of a diagnostic biopsy. Superiority of nivolumab could not be established in this study[26]. The randomized study CheckMate 227 compared three treatment arms in two populations of previously untreated patients with stage IV NSCLC. Depending on the PD-L1 status of the diagnostic tumor biopsies, patients were either treated with nivolumab, nivolumab plus ipilimumab, nivolumab plus platinum-based chemotherapy or chemotherapy. In patients selected by high tumor mutational burden (TMB) the combination of nivolumab plus ipilimumab demonstrated superior objective response and progression-free survival rates as compared to standard chemotherapy[13]. A recent report of this large study demonstrated increased overall survival in patients treated with nivolumab plus ipilimumab combination therapy over platinum-based chemotherapy[27]. This study provides definitive evidence that nivolumab-based ICI combinations can improve outcomes in patients with NSCLC. In the setting of resectable early stage NSCLC at least three studies have demonstrated feasibility, safety and preliminary efficacy of short courses of nivolumab- or atezolizumab-based treatment prior to surgical resection[1,22,23].

The safety and toxicities of nivolumab in NSCLC patients are well established by clinical studies and by extensive post-marketing experience. Importantly, management guidelines of immune-related toxicities that may occur under ICI therapy of NSCLC with nivolumab are well established, known in the medical oncology community and validated in their clinical efficacy[28]. In the studies CheckMate 017, 026 and 057 patients with NSCLC were treated with nivolumab at a dose of 3 mg/kg body weight q2w[7,8,26]. Extensive pharmacokinetic (PK) analyses of a fixed dose of nivolumab 240 mg q2w in subjects with less than 80 kg body weight predicted exposures to be within the range of observed exposures at body weight-adapted doses (up to 10 mg/kg q2w) used in the nivolumab clinical program, and are not considered to put subjects at increased risk. For subjects with greater body weights, the simulated ranges of exposures are also not expected to affect efficacy, because the exposures predicted following administration of a 240 mg q2w are on the flat part of the exposure-response curves for previously investigated tumors, including melanoma and NSCLC. Given the similarity of nivolumab PK across tumor

types and the similar exposures predicted following administration of a 240 mg flat dose compared to a dose of 3 mg/kg body weight, it was expected that the safety and efficacy profile of 240 mg nivolumab will be similar to that of 3 mg/kg nivolumab. Accordingly, the more recent study in NSCLC patients, CheckMate 227, used a flat dose of 240 mg nivolumab q2w for first-line nivolumab monotherapy of patients with PD-L1 positive NSCLC[13]. Collectively, these data support the use of nivolumab at a dose of 240 mg q2w in all arms of the present study, NEOpredict-Lung.

### 2.2.2 Rationale for combining nivolumab with relatlimab, dose selection and preliminary clinical data

LAG-3 (CD223) is a negative regulatory T cell receptor that is implicated in the control of conventional and regulatory T cells. LAG-3 is structurally related to CD4 in domain organization, and both CD4 and LAG-3 bind to MHC class II molecules. In human T cells LAG-3 is upregulated upon activation, and consecutively associates with reduced interleukin (IL)-2 secretion. In model systems LAG-3 and PD-1 synergistically suppress T cell stimulation, and simultaneous blockade of both molecules can improve T cell responses in a supraadditive manner. Based on these findings the monoclonal LAG-3 blocking antibody relatlimab was developed for combined therapy with a PD-1 blocking antibody, nivolumab.

For a complete summary of clinical information including safety and efficacy data of relatlimab and relatlimab in combination with nivolumab refer to the current relatlimab investigator´s brochure (IB). Based on all available data patients randomized to Arm B of the NEOpredict-Lung study will be treated with 2 cycles of relatlimab 80 mg plus nivolumab 240 mg both given on day 1 of a 14 day cycle.

### 2.2.3 Investigators and regulatory authorities will be continuously informed on updated data analyses and findings relevant to this study as they emerge. Updates of IBs of nivolumab and relatlimab will be regularly submitted to the investigators and the responsible regulatory authorities in each participating country.Mechanism of Action

#### 2.2.3.1 Nivolumab

Cancer immunotherapy rests on the premise that tumors can be recognized as foreign rather than as self and can be effectively attacked by an activated immune system. An effective immune response in this setting is thought to rely on immune surveillance of tumor antigens expressed on cancer cells that ultimately results in an adaptive immune response and cancer cell death. Meanwhile, tumor progression may depend upon acquisition of traits that allow cancer cells to evade immunosurveillance and escape effective innate and adaptive immune responses (Pardoll D. Does the immune system see tumors as foreign or self?[29–31]. Current immunotherapy efforts attempt to break the apparent tolerance of the immune system to tumor cells and antigens by either introducing cancer antigens by therapeutic vaccination or by modulating regulatory checkpoints of the immune system. T-cell stimulation is a complex process involving the integration of numerous positive as well as negative co-stimulatory signals in addition to antigen recognition by the T-cell receptor[32]. Collectively, these signals govern the

balance between T-cell activation and tolerance.

PD-1 is a member of the CD28 family of T-cell co-stimulatory receptors that also includes CD28, CTLA-4, ICOS, and BTLA[33]. PD-1 signaling has been shown to inhibit CD-28-mediated upregulation of Interleukin-2 (IL-2), IL-10, IL-13, interferon-γ and Bcl-xL. PD-1 expression also been noted to inhibit T-cell activation and expansion of previously activated cells. Evidence for a negative regulatory role of PD-1 comes from studies of PD-1 deficient mice, which develop a variety of autoimmune phenotypes[34]. These results suggest that PD-1 blockade has the potential to activate anti-self T-cell responses, but these responses are variable and dependent upon various host genetic factors. Thus, PD-1 deficiency or inhibition is not accompanied by a universal loss of tolerance to self-antigens.

*In vitro*, nivolumab binds to PD-1 with high affinity (EC50: 0.39 -2.62 nM), and inhibits the binding of PD-1 to its ligands PD-L1 and PD-L2 (IC50: ± 1 nM). Nivolumab binds specifically to PD-1 and not to related members of the CD28 family such as cytotoxic T-cell lymphocyte associated protein 4 (CTLA-4). Blockade of the PD-1 pathway by nivolumab results in a reproducible enhancement of both proliferation and IFN-γ release in the mixed lymphocyte reaction (MLR). Using a cytomegalovirus (CMV) re-stimulation assay with human peripheral blood mononuclear cells (PBMCs), the effect of nivolumab on antigen-specific recall response indicates that nivolumab augments IFN-γ secretion from CMV-specific memory T-cells in a dose-dependent manner versus isotype-matched control. In vivo blockade of PD-1 by a murine analog of nivolumab enhances the anti-tumor immune response and results in tumor rejection in several immunocompetent mouse tumor models (MC38, SA1/N, and PAN02).

#### 2.2.3.2 Relatlimab

Lymphocyte activation gene-3 (LAG-3; CD223) is also a type I transmembrane protein that is expressed on the cell surface of activated CD4+ and CD8+ T cells and subsets of NK and dendritic cells[35]. LAG-3 is closely related to CD4, which is a co-receptor for T helper cell activation. Both molecules have 4 extracellular Ig-like domains and require binding to their ligand, major histocompatibility complex (MHC) class II, for their functional activity[36,37]. In contrast to CD4, LAG-3 is only expressed on the cell surface of activated T cells and its cleavage from the cell surface terminates LAG-3 signaling[38]. LAG-3 can also be found as a soluble protein but it does not bind to MHC class II and its function is unknown. Combined inhibition of T cell checkpoint molecules, such as CTLA-4, PD-1, and LAG-3, in preclinical models provides synergistic improvement in T cell activity, control of virus replication, and tumor inhibition in animal models[39].

It has been reported that LAG-3 plays an important role in promoting regulatory T cell (Treg) activity and in negatively regulating T cell activation and proliferation[40,41]. Both natural and induced Treg express increased LAG-3, which is required for their maximal suppressive function. Furthermore, ectopic expression of LAG-3 on CD4+ effector T cells reduced their proliferative capacity and conferred on them regulatory potential against third party T cells. Recent studies have also shown that high LAG-3 expression on exhausted lymphocytic choriomeningitis virus (LCMV)-specific CD8+ T cells contributes to their unresponsive state and limits CD8+ T cell antitumor responses[42]. In fact, LAG-3 maintained tolerance to self and tumor antigens via direct effects on CD8+ T cells in two murine models. It is proposed that targeting

LAG-3 should be considered for the treatment of multiple malignancies in combination with nivolumab as a T cell-core therapy with the aim to: (1) increase the number, type, and duration of responses in tumors known to respond to ICI therapy; (2) rescue an adaptive response where patients are refractory to ICI therapy and have progressed clinically; and/or (3) enhance the antitumor immunity in malignancies associated with chronic viral infections (e.g., HPV, HCV, HBV, etc.).

Relatlimab is a human monoclonal $IgG_4$ antibody against LAG-3 including a stabilizing hinge mutation (S228P). Relatlimab binds with high affinity ($K_D$ 0.12 nM) to LAG-3 and prevents binding of LAG-3 to cells bearing its ligand, MHC calls II. By blocking this negative regulatory pathway relatlimab revokes LAG-3 mediated immune suppression and immune exhaustion. Relatlimab is expected to exert its therapeutic activity when combined with clinically active ICI such as nivolumab.

### 2.2.4 Summary of Clinical Pharmacology

#### 2.2.4.1 Nivolumab

The PK of nivolumab was studied in subjects over a dose range of 0.1 to 10mg/kg administered as a single dose or as multiple doses of nivolumab every 2 or 3 weeks. The geometric mean (% CV%) clearance was 9.5 mL/h (49.7%), geometric mean volume of distribution at steady state was 8.0 L (30.4%), and geometric mean elimination half-life (t1/2) was 26.7 days (101%). Steady-state concentrations of nivolumab were reached by 12 weeks when administered at 3mg/kg q2w, and systemic accumulation was approximately 3-fold. The exposure to nivolumab increased dose proportionally over the dose range of 0.1 to 10 mg/kg administered every 2 weeks.

The clearance of nivolumab increased with increasing body weight. The PK analysis suggested that the following factors had no clinically important effect on the clearance of nivolumab: age (29 to 87 years), gender, race, baseline LDH, PD-L1. Although Eastern Cooperation Oncology Group (ECOG) status, baseline glomerular filtration rate, albumin and body weight had an effect on nivolumab clearance, the effect was not clinically meaningful. When nivolumab is administered in combination with ipilimumab, the clearance of nivolumab was increased by 24%, whereas there was no effect on the clearance of ipilimumab. Additionally, PK and exposure response analyses have been performed to support use of 240 mg q2w dosing in addition to the 3 mg/kg q2w regimen. Using the PK model, exposure of nivolumab at 240 mg flat dose was identical to a dose of 3 mg/kg for subjects weighing 80 kg, which was the approximate median body weight in nivolumab clinical trials.

#### 2.2.4.2 Relatlimab

The PK of relatlimab was studied using the available serum concentration data from advanced solid tumor subjects in Study CA224-020, hematological malignancy subjects in Study CA224-022, and Japanese advanced solid tumor subjects in Study CA224-034. The PK of relatlimab after the first and ninth doses following IV infusion administered as relatlimab monotherapy or relatlimab in combination with nivolumab was characterized using noncompartmental analysis methods. Approximately dose-proportional increases in relatlimab exposure were seen in both monotherapy and combination therapy with nivolumab. However, a trend of slightly higher

exposure with increment of doses was also observed. A preliminary population PK analysis was performed using combined data from all 3 studies. Relatlimab population PK was best described by a 2-compartment model with parallel linear and nonlinear CL. The linear portion represents the nonspecific CL, and the nonlinear component represents target-mediated CL. The model estimated mean T-HALF was 19 days and the typical clearance was 13.7 mL/h. The PK of relatlimab or nivolumab was not altered when given in combination. In the population PK analysis, relatlimab CL changed over time and may have been confounded by the improved disease state. CL was lower in subjects with lower baseline albumin, higher ECOG, and higher baseline LDH levels. Consistent with the mechanism of elimination for monoclonal antibodies, both CL and volume of distribution were higher in patients with higher body weight. Exposure measures ($C_{max}$ and AUC) were higher in Japanese subjects in Study CA224-034 than the overall global population in Study CA224-020. However, after accounting for the effect of other significant covariates (ie, baseline body weight, sex, albumin, and LDH), the CL in Japanese subjects did not have clinically relevant differences from non-Japanese subjects. Currently available data suggest that the immunogenicity rate of anti-relatlimab antibodies was approximately 7% and 21% when administered alone and in combination with nivolumab, respectively. The neutralizing antibody assay is currently being developed to further assess neutralizing potential of anti-relatlimab anitbodies. The clinical significance of immunogenicity will be assessed using all available data on efficacy, safety, and time of immunogenicity occurrence.

Receptor occupancy (RO) of LAG-3 on CD8 T cells in the blood was measured at trough levels (14 days after dosing) during dose escalation of relatlimab monotherapy in Study CA224-022. Preliminary analysis of the data showed that RO had incrementally similar increases, with an average of 74%, 83%, and 93% RO after flat doses of 20, 80, and 240 mg, respectively. At the highest dose level tested of 800 mg, RO was 98.7%, representing a smaller incremental increase of 6% from the lower 240 mg dose level.

### 2.2.5 Safety Summary

The overall safety experience with nivolumab, as monotherapy or in combination with other therapeutics, is based on experience in more than 23,000 subjects treated to date. Extensive details on the safety profile of nivolumab, including results from other clinical studies, are available in the current nivolumab investigator brochure (IB), and will not be repeated herein. A pattern of immune-related AEs has been defined, for which management algorithms have been developed. Most high-grade events were manageable with the use of corticosteroids or hormone replacement therapy (endocrinopathies) as instructed in these algorithms. For additional material, see the current nivolumab IB, which is regularly updated.

Overall, the safety profile of nivolumab alone or in combination with other therapeutic agents is manageable and generally consistent across completed and ongoing clinical trials, with no maximal tolerated dose (MTD) reached at any dose tested up to 10mg/kg. Most AEs were low-grade (Grade 1 to 2) with relatively few related high-grade (Grade 3 to 4) AEs. There was no pattern in the incidence, severity, or causality of AEs with respect to nivolumab dose level. Results to date suggest that the safety profile of nivolumab/ relatlimab combination therapy is consistent with the mechanisms of action of nivolumab and ipilimumab. The nature of the AEs is

similar to that observed with either agent used as monotherapy; however, both frequency and severity of most AEs are increased with the combination.

Most adverse events and serious adverse events were reversible and manageable by withholding study drug administration, providing standard medical care, and following established algorithms for immune-related adverse events. In summary, the safety profile of relatlimab/nivolumab combination therapy is consistent with the one of clinically established immune checkpoint inhibitor therapies.

Details on the safety profiles of nivolumab plus relatlimab, including results from clinical studies, are also available in the current nivolumab and relatlimab investigator brochures, which are regularly updated.

## 2.3 Risk benefit assessment

Lung cancer is the leading cancer fatality. The incidence of new lung cancers is plateauing or declining in Western countries due to some decrease in cigarette smoking. On a global scale lung cancer is on the rise, and in Western countries, lung cancer deaths still by far exceed the number of deaths from the second and third most fatal cancer entities (Krebs in Deutschland für 2013/2014. 11. Ausgabe. Robert Koch-Institut (Hrsg) und die Gesellschaft der epidemiologischen Krebsregister in Deutschland e.V. (Hrsg). Berlin, 2017). Approximately 85% of all lung cancers are grouped as NSCLC. When diagnosed at a localized disease stage, anatomic resection with systematic lymph node dissection is the preferred curative treatment approach. However, many resected patients experience disease recurrence and ultimately succumb to their NSCLC. Currently, there is strong evidence for risk reduction in resectable NSCLC by cisplatin-based chemotherapy in locally or regionally advanced cases. The clinical effect of adjuvant or neoadjuvant chemotherapy is moderate with an increase in 5-year OS in the range of up to 10% in prospective studies[4–6]. Data in elderly and comorbid patients are scarce, and adjuvant therapy is not administered in many patients because of severe comorbidities, prolonged postoperative recovery and patient preference. Hence, there is a high medical need for new effective and tolerable therapies to reduce the risk of disease recurrence in patients with resectable NSCLC. This is particularly true for patients with high risk features as defined by tumor size, presence multiple tumor lesions and/or lymph node metastasis.

ICIs are a new class of therapeutics with proven clinical activity over a broad range of cancer entities. In advanced or metastatic NSCLC, antibodies targeting PD-1 (nivolumab, pembrolizumab) and PD-L1 (atezolizumab) have shown superiority over standard chemotherapy in several pivotal phase III studies in patients with advanced/metastatic or recurrent NSCLC[7–11]. More recently, the clinical efficacy of ICI combination therapy with nivolumab and ipilimumab was demonstrated in a subgroup of NSCLC patients characterized by high tumor mutational burden, and the ICI combination showed superior overall survival as compared to standard platinum-based first-line chemotherapy[13, 27]. Several studies of adjuvant ICI therapy in patients with localized NSCLC have been conducted or are currently ongoing. Consolidation therapy with the anti-PD-L1 antibody durvalumab significantly improved progression-free survival and time to

death or metastasis in patients with locally advanced NSCLC who were treated with concurred radiochemotherapy[12,17], thus demonstrating safety and efficacy of ICI therapy in the curative setting. Overall, ICI therapy was safe and had a favorable toxicity profile in patients with localized and metastatic NSCLC. A recent pilot study explored preoperative therapy with two courses of nivolumab in patients planned for curative resection of NSCLC. Safety, feasibility and a high rate of histopathological responses were demonstrated in a still limited and highly selected patient population. Importantly, 20 of 21 eligible patients subsequently underwent complete tumor resection[1]. This promising early report on the use of an ICI in the neoadjuvant setting has already lead to the start of follow-up studies. Additional studies of preoperative atezolizumab, pembrolizumab, nivolumab or nivolumab combined with ipilimumab are conducted in patients with early stage resectable cancer. Safety and preliminary efficacy data of such trials in NSCLC[22,23], colon cancer[24]and urothelial bladder cancer[25] were presented and confirm the feasibility and preliminary safety of the concept of preoperative ICI therapy in NSCLC and additional cancer entities. Substantial clinical activity was demonstrated in multiple trials, with a particular emphasis on very durable responses and disease control in subsets of patients. Such long-term benefits in metastatic NSCLC have not been previously achieved with cytotoxic chemotherapies or targeted therapies. This provides a strong rationale to explore and refine the power of ICI therapy in curative treatment settings of NSCLC.

Today, ICI therapy is well established in thoracic oncology. Physicians treating NSCLC patients are experienced with this modality. They are well aware of its toxicities and potential hazards. A pattern of immune-related AEs has been defined across all cancer entities treated with ICIs, for which well-accepted management algorithms have been developed. Most high-grade events were manageable with the use of corticosteroids or hormone replacement therapy (endocrinopathies) as instructed in these management algorithms. This scenario applies to both arms of the NEOpredict-Lung study, as they are exploring treatments which act indirectly by reinvigoration of immune responses. It is not expected that the addition of relatlimab to nivolumab in Arm B will significantly alter the spectrum of potential treatment-associated toxicities. This is based on current clinical experience with relatlimab as single agent or combined with nivolumab from ongoing studies. Importantly, the mode of action of this antibody combination is similar. Details concerning nonclinical toxicology and safety pharmacology of relatlimab combined with nivolumab are provided in the respective investigator brochure.

The study NEOpredict-Lung will assess investigational (relatlimab) and marketed (nivolumab) drugs whose effects on pregnancy are not yet known or fully defined. Contraception is therefore required for participants who are WOCBP or male, and for female partners of male participants who are WOCBP. Contraception guidelines presented in Appendix IV were initially developed for the nivolumab development program and apply to all female participants and partners of male participants who could be exposed to the drug and who could become pregnant both during treatment and during a defined period after study treatment. In these nivolumab program contraceptive guidelines, hormone-based contraception (e.g., oral hormone-based contraceptives or hormone-releasing intrauterine device [IUD]) is considered to be a highly effective method for WOCBP who are participants in a nivolumab program study.

Throughout the NEOpredict-Lung study the rate of patients who do not proceed to curative surgery within the predefined time frame is continuously monitored for each study arm. This

allows early closure of unfeasible treatment arms based on prespecified criteria. To ensure an ongoing favorable risk/benefit assessment for subjects enrolled in NEOpredict-Lung, an independent Data Safety Monitoring Board (DSMB) will be established to monitor the safety and clinical activity of the treatments throughout the conduct of the trial. A recent analysis based on data from close to 5,000 patients with early stage NSCLC demonstrated a median time from diagnosis to surgery of 38 days. At multivariate analysis, patients who had surgery later than 38 days after diagnosis had numerically inferior OS at 5 years. However, the threshold time associated with statistically significant worse survival was not reached before 90 days [43]. The addition of the maximum screening (28 days) and preoperative treatment periods (42 days) of the NEOpredict-Lung study amounts 70 days. This still is considerably shorter than the threshold time of 90 days for a delay in surgery of NSCLC which was associated with worse survival. This supports the time lines foreseen in this study, which puts highest priority on patient safety.

# 3 Objective of the study

## 3.1 Primary objective

The primary objective of this study is to determine the feasibility of four weeks of preoperative immunotherapy with nivolumab, and nivolumab plus relatlimab in patients with early stage or locally advanced non-small cell lung cancer eligible for curative resection.

Based on the results of a pilot study[1] it is expected that at least 26 of 30 patients treated in each study arm will undergo curatively intended surgery within 6 weeks of initiation of study treatment (first given on day 1). A study arm will be declared non-feasible if 5 or more patients experience a delay of curatively intended surgery beyond day 43, either due to toxicities or disease progression. Continuous monitoring of prespecified stopping boundaries facilitates early termination of non-feasible study arm to reduce patient risks.

## 3.2 Secondary and other objectives

- Estimation of pathological tumor response rate (rate of complete pathological responses defined as absence of viable tumor cells on routine hematoxylin and eosin staining of resected tumors and lymph nodes; rate of major pathological responses defined as 10% or less viable tumor cells on routine hematoxylin and eosin staining of resected tumors)
- Estimation of complete (R0) resection rate
- Assessment of radiologic response on computed tomography per Response Evaluation Criteria in Solid Tumors (RECIST) version 1.1
- Assessment of disease-free survival rate at 12 months per RECIST version 1.1
- Assessment of overall survival rate at 12 months
- Assessment of safety and tolerability of preoperative immunotherapy

- Estimation of morbidity and mortality within 90 days of surgery
- Exploratory translational analyses for investigation of immunomodulatory and anticancer activity of preoperative treatment with nivolumab and nivolumab/relatlimab combination therapy

# 4 Organizational and administrative aspects of the study

## 4.1 Sponsor

| | |
|---|---|
| Sponsor: | University Hospital Essen<br>Hufelandstrasse 55<br>45147 Essen, Germany |
| Represented by: | Thorsten Kaatze |

## 4.2 Statistics

| | |
|---|---|
| Statistician: | Dr. Christine Windemuth-Kieselbach<br>Alcedis GmbH<br>Winchesterstr. 3<br>35394 Gießen<br>Germany |

The objective of this study is to determine the feasibility of two cycles of preoperative immunotherapy in patients eligible for curative anatomic resection of NSCLC. Sequential boundaries will be used to monitor the non-feasibility rate. The accrual for each study arm will be halted if excessive numbers of delays of curative surgery beyond 6 weeks from initiation of study treatment are seen, that is, if the number of delayed surgeries is equal to or exceeds $b_n$ out of $n$ patients with sufficient follow-up (see table). This is a Pocock-type stopping boundary that yields the probability of crossing the boundary at most 0.05 (probability of early stopping) when the rate of delayed surgeries is equal to the acceptable rate 0.05 (event probability $\theta$).

| | | | | | | | | | | | | | | | | | | | | |
|---|---|---|---|---|---|---|---|---|---|---|---|---|---|---|---|---|---|---|---|---|
| $n$ | 1 | 2 | 3 | 4 | 5 | 6 | 7 | 8 | 9 | 10 | 11 | 12 | 13 | 14 | 15 | 16 | 17 | 18 | 19 | 20 |
| $b_n$ | - | 2 | 2 | 2 | 3 | 3 | 3 | 3 | 3 | 3 | 3 | 3 | 4 | 4 | 4 | 4 | 4 | 4 | 4 | 4 |
| $n$ | 21 | 22 | 23 | 24 | 25 | 26 | 27 | 28 | 29 | 30 | | | | | | | | | | |
| $b_n$ | 4 | 4 | 5 | 5 | 5 | 5 | 5 | 5 | 5 | 5 | | | | | | | | | | |

This boundary is equivalent to testing the null hypothesis after each patient, that the event rate is equal to 0.05, using a one-sided level 0.022387 test.

All secondary parameters will be evaluated in an explorative or descriptive manner, providing means, medians, ranges, standard deviations and/or confidence intervals. All p-values will be two-sided if not stated otherwise.

The amount and subsets of tumor infiltrating lymphocytes and peripheral immune cells before and after therapy will be analyzed using t-test, Mann-Whitney-U test or ANOVA. Overall response rate (ORR), pathological response rates (complete pRR and major pRR), toxicity and other event rates in each arm will be compared using Fishers exact test and $\chi^2$ test; odds ratios with confidence intervals and p-value will be provided.

DFS and OS will be analyzed using Kaplan-Meier methods and compared by log rank test. Univariate and multivariate analyses will eventually be performed by suitable regression models (logistic regression, proportional hazard regression model).

## 4.3 Investigators and study sites

**Global Coordinating Investigator and Coordinating Investigator Germany** (*Leiter der klinischen Prüfung* according to German Drug Law)


| | |
|---|---|
| Name: | Professor Dr. med. Martin Schuler |
| Address: | West German Cancer Center, Department of Medical Oncology, University Hospital Essen, Hufelandstrasse 55, 45147 Essen, Germany |
| Phone: | +49 (0) 201-723 2000 |
| Email: | martin.schuler@uk-essen.de |


**Coordinating Investigator The Netherlands**


| | |
|---|---|
| Name: | Professor Paul Baas, M.D., Ph.D. |
| Address: | Department of Thoracic Oncology, The Netherlands Cancer Institute, Plesmanlaan 121, 1066CX Amsterdam, The Netherlands |
| Phone: | +31 (0) 205129098 |
| Email: | p.baas@nki.nl |


**Coordinating Investigator Belgium**


| | |
|---|---|
| Name: | Dr. Kristof Cuppens |

| | |
|---|---|
| Address: | Department of Pulmonology, Jessa Hospital Hasselt,<br>Stadsomvaart 11, B3500 Hasselt, Belgium |
| Phone: | +32 (0) 472565987 |
| Email: | kristof.cuppens@jessazh.be |


All other study personnel not included in this section are identified in a separate personnel list (not part of this clinical study protocol) as appropriate. This list will be updated as needed; an abbreviated version with personnel relevant for the center will be available in the center's investigator site file (ISF) and the trial master file (TMF).

Requirements for investigators and study sites

The study site will be required to have an investigator with at least 2 years' experience in conducting clinical trials according to Good Clinical Practice (GCP) as well as a substitute with comparable qualifications. The site must have a pool of suitable patients who can be recruited for the study.

All investigators must be qualified physicians. Each physician must supply her or his curriculum vitae (updated) for the TMF.

Investigators also must

- have special experience in the study indication and respective treatment as well as diagnostic procedures,
- know main features of the law in Germany ("*Arzneimittelgesetz (AMG)*") or equivalent in the center's country as well as legal and scientific basics of clinical trials in the European union,
- know the study protocol.

The investigator must sign the protocol signature sheet before subject recruitment may start. Likewise, all protocol amendments must be signed and dated by the investigator before coming into effect.

## 4.4 Financing

This is an investigator-initiated, international, multicenter study, which receives financial support by a research grant from Bristol–Myers Squibb

# 5 Study design

## 5.1 Design overview

This is an international, multicenter, open-label, randomized, two-armed, modular phase II study. Patients with histologically confirmed non-small cell lung cancer (NSCLC) of clinical stages I B, II and selected stage III A (T3 N1, T4 with satellite nodule in the same lung N0/N1, selected T1a-T2b N2 cases considered suitable for primary surgical approach by the multidisciplinary tumor board), who are eligible for anatomic resection will be included.

For treatment details see Section 7.1.

The study will involve a total of 3 participating sites in Germany, Netherlands and Belgium. It is planned to simultaneously open study Arms A and B. Patients will be randomized between Arm A or Arm B to include in total up to 60 evaluable subjects.

The study consists of a screening visit for all subjects.

Subjects in all arms will receive 2 preoperative treatment cycles with a cycle duration of 14 days. Study treatment consists of nivolumab q2w in Arm A, and nivolumab q2w plus relatlimab q2w in Arm B.

Following neoadjuvant study treatment all patients will proceed to standard of care surgery, which is followed by standard of care adjuvant therapy if clinically indicated. Surgery and adjuvant therapy are not part of the NEOpredict-Lung study treatment.

Subjects will have follow-up visits until after 12 months of study treatment every 3 months and an end-of-study visit. After 12 months, standard of care follow-up will be provided at the study centers as recommended by the applicable guidelines (e.g., Leitlinienprogramm Onkologie [Deutsche Krebsgesellschaft, Deutsche Krebshilfe, AWMF]: Prävention, Diagnostik, Therapie und Nachsorge des Lungenkarzinoms, Langversion 1.0, 2018, AWMF-Registernummer: 020/007OL). For each subject, the total duration of the study will be approximately 13 months including follow-up.

For the study as a whole, the primary outcome will be continuously evaluated for each treatment arm. Clinical outcomes will be evaluated when the last patient will have completed the follow-up period. Early termination of a study arm will occur once prespecified stopping boundaries are crossed.

The study design and treatments administered are displayed in Figure 1.

**Figure 1: Study design NEOpredict-Lung**

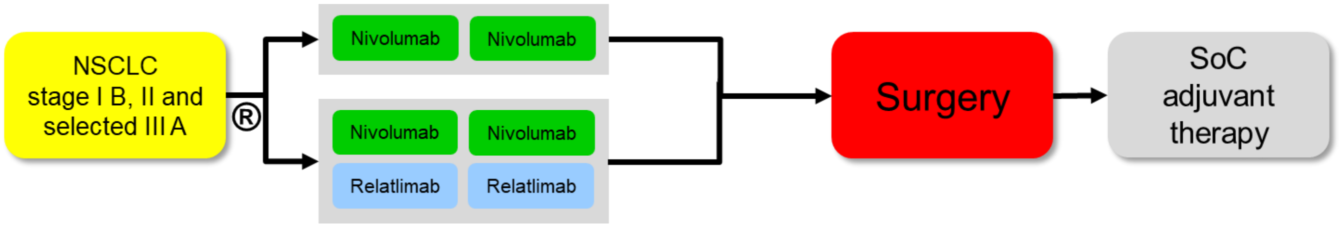


NSCLC – Non-small cell lung cancer; Soc – Standard of care.

## 5.2 Study variables

## 5.3 Primary variable

**Primary variable**

Primary variable is the number of patients undergoing curatively intended surgery of non-small cell lung cancer within 43 days of initiation of study therapy (first given on day 1).

### 5.3.1 Secondary variables

- Objective response rate (RECIST 1.1)
- Pathological response rate (complete pathological responses defined as absence of viable tumor cells on routine hematoxylin and eosin staining of resected tumors and lymph nodes; rate of major pathological responses defined as 10% or less viable tumor cells on routine hematoxylin and eosin staining of resected tumors)
- R0 resection rate
- Radiomorphologic response per RECIST 1.1
- Disease-free survival rate at 12 months per RECIST version 1.1
- Overall survival rate at 12 months
- Safety and tolerability of preoperative immunotherapy
- Morbidity and mortality within 90 days of curative surgery

### 5.3.2 Translational analyses

The complete translational program is described in the NEOpredict-Lung Translational Protocol (separate research protocol). Systematic sample acquisition and biobanking is an integral part of the NEOpredict-Lung study protocol. In brief, the following translational analyses are performed:

- Genomic profiling for determination of tumor mutational burden (TMB), HLA status, neoantigens, and HLA loss
- Phenotyping, T cell receptor sequencing, and functional ex vivo studies of tumor-infiltrating lymphocytes from resected tumors
- Phenotyping and ex vivo analyses of myeloid cells from resected tumors and peripheral blood
- Multiplexed profiling of RNA and protein expression in surplus material from pretreatment tumor biopsies and resected tumors by Digital Spatial Profiling
- Monitoring of metabolic changes induced by study therapy by quantitative LC/MS in sequentially acquired plasma samples
- Monitoring the composition of exhaled volatile organic substances using the eNose (www.enose.nl) or a comparable device
- Monitoring of dynamic changes of tumor lesions in response to study therapy by functional imaging using MRI and PET technology

## 5.4 Justification of study design

For study objectives, see Section 4.

The present trial is designed as an international, multicenter, randomized, two arm, modular phase II study. Patients with histologically or cytologically (EBUS-TBNA) confirmed, surgically resectable NSCLC of stage I B, II or selected stages III A will be included.

Preoperative therapy of surgically resectable NSCLC provides an ideal platform for comprehensive clinical and translational investigation of ICI therapies. As the study is conducted in a curative setting, highest priority is put on patient safety. A recent analysis based on data from close to 5,000 patients with early stage NSCLC demonstrated a median time from diagnosis to surgery of 38 days. At multivariate analysis, patients who had surgery later than 38 days after diagnosis had numerically inferior OS at 5 years. However, the threshold time associated with statistically significant worse survival was not reached before 90 days[42]. Therefore, the primary study endpoint is feasibility of a short period (28 days) of preoperative ICI therapy in patients eligible for curative resection of NSCLC. On an individual patient level feasibility is accepted if curative surgery is conducted no later than 42 days after initiation of study treatment. The screening period allows a maximum of 28 days, which most likely is not required in this clinical setting as all patients will have obtained recent histological confirmation and imaging studies as part of the standard of care diagnostic process in newly diagnosed NSCLC. On a study population level, feasibility is continuously monitored for each study arm. This allows early closure of a study arm in case the prespecified stopping boundary for acceptance of feasibility is crossed.

With respect to study treatments Arm A uses two doses of preoperative nivolumab to (i) confirm results of recently published pilot studies [1 23] and (ii) to serve as reference for combination therapy explored in Arm B. The data obtained in Arm A will facilitate the detection of an additive or synergistic value of the combination partner relatlimab with respect to clinical and translational endpoints. Further, Arm A will serve as reference for the impact of the combination partner on

the primary study endpoint, feasibility, as well as for safety and toxicity endpoints.

Nivolumab clearly is a safe and active agent in advanced and recurrent NSCLC. Recently published pilot studies have demonstrated early signs of efficacy in preoperative treatment of patients with resectable NSCLC [23]. Nivolumab's anticancer activity is mediated by indirect mechanisms of action leading to reinvigoration of anticancer immunity. Combining nivolumab with relatlimab is expected to further enhance this activity. The design of study NEOpredict-Lung allows the comparison of combination therapy with the nivolumab reference (Arm A).

## 5.5 End of the clinical study

As for this study, the primary outcome is continuously assessed for each study arm. In case of non-feasibility of a study arm, this may be terminated early. Main secondary outcomes will be analyzed for each study arm once the last patient underwent tumor resection. The end of the study as a whole will be the date of last patient last end-of-study visit at follow-up / clean database.

# 6 Study population

## 6.1 Justification of selection criteria

The selection criteria are chosen to ensure that subjects with specific risks for administration of the study medication and / or subjects with conditions which may have an impact on the aims of the study are excluded. Patients will be identified by the investigators from the patient population of the study centers according to the inclusion and exclusion criteria. The annual case load of the study centers is sufficiently high to ensure enrolment as planned.

## 6.2 Justification of gender selection

Male and female subjects will be included in the present study representing the population of patients with histological/cytologically confirmed NSCLC with clinical stages I B, II or selected stages III A who are eligible for curative surgery without any gender preference.

## 6.3 Inclusion criteria

Subjects must fulfill all of the following criteria before inclusion in the study:

**Principal inclusion criteria**

- Patients with histologically (core biopsy) or cytologically (e.g. bronchoscopy-guided biopsy) confirmed non-small cell lung cancer (NSCLC) eligible for anatomic resection, with the following specifications:
    - Clinical stages I B, II and selected stage III A (T3 N1, T4 with satellite nodule in the same lung N0/N1, selected T1a-T2b N2 cases considered suitable for primary surgical approach by the multidisciplinary tumor board) according to UICC 8th edition.
- Males and females, ages ≥ 18 years, inclusive
    - Women of childbearing potential (WOCBP) must have a negative serum or urine pregnancy test (minimum sensitivity 25 IU/L or equivalent units of human chorionic gonadotropin [hCG]) within 24 hours prior to the start of study treatment.
    - Women of childbearing potential (WOCBP) must agree to follow instructions for highly effective method(s) of contraception for the duration of treatment with study medication plus 5 half-lives of study treatment plus 30 days (duration of ovulatory cycle) post treatment completion for a total of 165 days (approximately 24 weeks) (Appendix IV). This applies to both treatment arms, nivolumab monotherapy and nivolumab/relatlimab combination therapy.
    - Males who are sexually active with WOCBP must agree to follow instructions for

highly active method(s) of contraception and fetal protection (Appendix IV) for the duration of treatment with study medication plus 5 half-lives of the study treatment plus 90 days (duration of sperm turnover) for a total of 225 days (approximately 33 weeks) post-treatment completion. In addition, male participants must be willing to refrain from sperm donation during this time. This applies to both treatment arms, nivolumab monotherapy and nivolumab/relatlimab combination therapy.

- ECOG ≤ 1
- Exclusion of extensive mediastinal lymph node metastases (multilevel N2, N3) by PET/CT and/or mediastinal lymph node sampling by EBUS-TBNA and/or staging mediastinoscopy.
- Exclusion of distant metastases by standard of care imaging studies, which include but are not limited to PET/CT or PET/MRI, or CT or MRI of thorax, abdomen, pelvis, and bone scan. Asymptomatic brain metastases will be excluded by MRI or contrast-enhanced CT as indicated by current guidelines.
- Measurable target tumor prior to immunotherapy using standard imaging techniques.
- Sufficient pulmonary function to undergo curative lung cancer surgery, ppFEV1>30%, ppDLCO>30%, ppVO2max ≥ 10 ml/min/kg (if CPET was mandated per local guidelines)
- Adequate hematological, hepatic and renal function parameters:
    - Leukocytes ≥ 2,000/mm³, platelets ≥ 100,000/mm³, absolute neutrophil count (ANC) ≥ 1,500/µL, hemoglobin ≥ 9 g/dL (5.58 mmol/L),
    - Anti-platelet therapy (such as but not limited to clopidogrel) should be discontinued pre-operatively according to local standards. If this therapy cannot be interrupted due to severe cardiovascular comorbidity, patient is ineligible for the trial
    - Adequate coagulation function as defined by International Normalized Ratio (INR) ≤ 1.5, and a partial thromboplastin time (PTT) ≤ 5 seconds above the upper limit of normal (ULN) (unless receiving anticoagulation therapy). Patients receiving warfarin/phenprocoumon or direct oral anticoagulants are to be bridged according to local standards and have achieved stable coagulation profile prior to surgery.
    - Serum creatinine ≤ 1.5 x upper limit of normal
    - Bilirubin ≤ 1.5 x upper limit of normal, AST and ALT ≤ 3.0 x upper limit of normal, alkaline phosphatase ≤ 6 x upper limit of normal
- Sufficient cardiac left ventricular defined as LVEF ≥ 50% documented either by echocardiography or MUGA (echocardiography preferred test, MUGA not used in German site) within 6 months before first administration of study drug
- Patient able and willing to provide written informed consent and to comply with the study protocol and with the planned surgical procedures

## 6.4 Exclusion criteria

Subjects are to be excluded from the study if they display any of the following criteria:

- Active or history of autoimmune disease or immune deficiency, including, but not limited to, myasthenia gravis, myositis, autoimmune hepatitis, systemic lupus erythematosus, rheumatoid arthritis, inflammatory bowel disease, anti-phospholipid antibody syndrome, Wegener granulomatosis, Sjögren syndrome, Guillain-Barré syndrome, multiple sclerosis, vasculitis, or glomerulonephritis.

  Patients with the following conditions are **not excluded** from participation:

  - Patients with a history of autoimmune-related hypothyroidism who are on thyroid-replacement hormone are eligible for the study.
  - Patients with controlled type 1 diabetes mellitus who are on an insulin regimen are eligible for the study.
  - Skin disorders (such as vitiligo, psoriasis, or alopecia) not requiring systemic immunosuppressive treatment, in particular corticosteroids are permitted to enroll.
- Subjects with a condition requiring systemic treatment with either corticosteroids (> 10 mg daily prednisone equivalents) or other immunosuppressive medications within 14 days of study drug administration. Inhaled or topical steroids, and adrenal replacement doses > 10 mg daily prednisone equivalents are permitted in the absence of active autoimmune disease.
- Subjects who have undergone organ transplant or allogeneic stem cell transplantation.
- ppFEV1<30%, ppDLCO<30%, ppVO2max < 10 ml/min/kg (if CPET was mandated per local guidelines)
- Uncontrolled or significant cardiovascular disease including, but not limited to, any of the following:
  - Myocardial infarction (MI) or stroke/transient ischemic attack (TIA) within the 6 months prior to consent
  - Uncontrolled angina within the 3 months prior to consent
  - Any history of clinically significant arrhythmias (such as ventricular tachycardia, ventricular fibrillation, or torsades de pointes)
  - QTc prolongation > 480 msec
  - Pulmonary hypertension (sPAP >35 mmHg)
- History of other clinically significant cardiovascular disease (i.e., cardiomyopathy, congestive heart failure with New York Heart Association [NYHA] functional classification III-IV, pericarditis, significant pericardial effusion, significant coronary stent occlusion, deep venous thrombosis, etc )
- Cardiovascular disease-related requirement for daily supplemental oxygen

- History of two or more myocardial infarctions or two or more coronary revascularization procedures
- Subjects with history of myocarditis, regardless of etiology
- Troponin T (TnT) or I (TnI) > 2 × institutional upper limit of normal (ULN). Subjects with TnT or TnI levels between > 1 to 2 × ULN will be permitted if repeat levels within 24 hours are within ULN. If TnT or TnI levels are >1 to 2 × ULN within 24 hours, the subject may undergo a cardiac evaluation and be considered for treatment, following a discussion with the coordinating investigator or designee. When repeat levels within 24 hours are not available, a repeat test should be conducted as soon as possible. If TnT or TnI repeat levels beyond 24 hours are < 2 x ULN, the subject may undergo a cardiac evaluation and be considered for treatment, following a discussion with the coordinating investigator or designee
- Patients with active neurological diease should be excluded.
- Active malignancy or a prior malignancy within the past 3 years.

  Patients with the following conditions are **not excluded** from participation:

  - Patients with completely resected basal cell carcinoma, cutaneous squamous cell carcinoma, cervical carcinoma in-situ, breast carcinoma in-situ, and patients with isolated elevation in prostate-specific antigen or low risk prostate cancer managed with active surveillance or watchful waiting in the absence of radiographic evidence of metastatic prostate cancer.
- Known history of positive test for human immunodeficiency virus (HIV-1 and HIV-2) or known acquired immunodeficiency syndrome (AIDS). NOTE: Testing for HIV-1 and HIV-2 must be performed at screening.
- Any positive test result for hepatitis B virus or hepatitis C virus indicating presence of virus, e.g., hepatitis B surface antigen (HBsAg, Australia antigen) positive, or hepatitis C antibody (anti-HCV) positive (except if HCV RNA negative).
- Any other disease, metabolic dysfunction, physical examination finding, or clinical laboratory finding that contraindicates the use of an investigational drug, may affect the interpretation of the results, or may render the patient at high risk from treatment complications
- Receipt of live attenuated vaccine within 30 days prior to the first dose of study medication.
- Peripheral polyneuropathy NCI CTCAE Grade ≥ 2
- History of gastric perforation or fistulae in past 6 months
- Serious or non-healing wound, ulcer, or bone fracture within 28 days prior to enrollment.
- The patient has undergone major surgery within 28 days prior to enrollment except staging mediastinoscopy, diagnostic VATS or implantation of a venous port-system.
- Any other concurrent preoperative antineoplastic treatment including irradiation
- Pregnant women
- Breastfeeding women

- Insufficient cardiac left ventricular function defined as LVEF<50% by echocardiography (outside Germany: or MUGA)
- A confirmed history of encephalitis, meningitis, or uncontrolled seizures in the year prior to informed consent.
- Subjects with history of severe or life-threatening toxicity (grade 3 or 4) related to prior immune therapy (e.g. anti-CTLA-4 or anti-PD-1/PD-L1 treatment or any other antibody or drug specifically targeting T-cell co-stimulation or immune checkpoint pathways) except those that are unlikely to re-occur with standard countermeasures (e.g., hormone replacement after endocrinopathy.
- Subjects with history severe or life-threatening (grade 3 or 4) infusion-related reactions to prior immune therapy
- Prior treatment with LAG-3 targeted agent.

**Other**

- Participation in another interventional clinical study within the last 3 months prior to inclusion or simultaneous participation in other clinical studies
- Previous treatment with nivolumab or relatlimab
- Previous immunotherapy for lung cancer
- Criteria which in the opinion of the investigator preclude participation for scientific reasons, for reasons of compliance, or for reasons of the subject’s safety
- Any contraindications against nivolumab or relatlimab

## 6.5 Withdrawal and replacement of study subjects

Subjects will be withdrawn from the study for the following reasons:

- At their own request; at any time during the study and without giving reasons, a subject may decline to participate further. The subject will not suffer any disadvantage for further treatment as a result
- If, in the investigator's opinion, continuation of study would bear a potential health risk for the patient
- At the specific request of the sponsor
- In case of violation of in- / exclusion criteria; if the subject develops conditions which would have prevented his / her entry into the study according to the in- / exclusion criteria, she / he must be withdrawn immediately if safety is concerned; in other cases, the investigator will decide whether there is a conflict with the study objectives
- Non-compliance with the study conditions or instructions from the investigator’s team

- In case of suspected or verified pregnancy

A subject who discontinues study participation prematurely for any reason is defined as ”dropout” if the subject has already received study medication. A subject who terminates the study for any reason (e.g. failure to satisfy the selection criteria) before first treatment with study medication is regarded a “screening failure”.

Any subject removed from the study will remain under medical supervision until discharge is medically acceptable. In addition, patient’s will be followed-up using a registry for further assessment of OS and DFS, if patients did provide consent to do so. In all cases, the reason for withdrawal must be recorded in the case report form (eCRF) and in the subject’s medical records.

Details for the premature termination of the whole study or study parts (e. g. treatment arms, dose steps) are provided in Section 14.

### 6.6 Replacement

Subjects who prematurely discontinue participation before resection for reasons other than associated with study therapy (e.g. patient refusal, administrative issues) will be replaced only if the number of subjects with resection becomes/is expected to become less than 30 patients in each arm.

### 6.7 Subject identification

Following written informed consent,  each subject will be assigned a screening number. Screening numbers are sequentially generated at each study center (consisting of the number of the study center followed by a three-digit number in ascending order).

## 7 Treatment / Investigational drug

### 7.1 Treatments to be given

**Arm A**: Nivolumab 2 cycles, every two weeks (q2w)
  - nivolumab 240mg i.v. over 30 min

**Arm B:** Nivolumab/Relatlimab 2 cycles, every two weeks (q2w)
  - nivolumab 240mg i.v. over 30 min
  - relatlimab 80 mg i.v. over 30 min (within 30 min of nivolumab)

**Table 1: Treatments**

| Treatment arm | Study drug | Dose | Formulation / RoA | Frequency of administration | No of subjects treated |
|---|---|---|---|---|---|
| A | Nivolumab | 240 mg | i.v. | 2 cycles, q2w | 30 |
| B | Nivolumab<br>Relatlimab | 240mg<br>80mg | i.v.<br>i.v. | 2 cycles, q2w<br>2 cycles, q2w | 30 |

i.v.=intravenous; q2w=every second week; RoA=Route of administration

## 7.2 Description of the Investigational Medicinal Products (IMPs)

### 7.2.1 Test drug

Nivolumab is indicated for the treatment of melanoma, NSCLC, renal cell cancer, Hodgkin lymphoma, squamous head and neck cancer and urothelial cancer. In the framework of the present study nivolumab will be administered as described in Section 7.7.

Relatlimab is an investigational agent blocking the LAG-3 receptor. In the framework of the present study relatlimab will be administered in combination with nivolumab as described in Section 7.7

**Table 2: Test drug**

| Product Description/ Class and Dosage Form | Relatlimab Injection (10 mg/mL) | Nivolumab Injection (10 mg/mL) |
|---|---|---|
| **Trade Name** | n.a. | OPDIVO® |
| **Potency** | 10 mg/mL | 10 mg/mL |
| **IP/Non-IMP** | IP | IP |
| **Blinded or Open-label** | Open-label, 4 vials per carton/ 100 mg/vial (10 mL vial)<br>or<br>Open-label, 4 vials per carton/ 80 mg/vial (8 mL vial) | Open-label, 4 vials per carton/ 100 mg/vial (10 mL vial) |
| **Packaging/Appearance** | Colorless to pale yellow liquid, clear to opalescent, light (few) particulates (consistent in appearance to protein particulates) may be present. | Clear to opalescent, colorless to pale yellow liquid. Light (few) particulates may be present. |
| **Storage Conditions (per label)** | Refer to the label on container | Refer to the label on container |
| **Manufacturer** | Bristol-Myers Squibb | Bristol-Myers Squibb |

### 7.2.2 Labelling of Investigational Medicinal Product (IMP)

Bristol-Myers Squibb (BMS) will provide both nivolumab and relatlimab clinical supplies at no cost for this study. All study drugs will be distributed and labeled by the manufacturer Bristol-Myers Squibb (BMS). A complete record of batch numbers and expiry dates of all study treatment will be maintained in the trial master file.

## 7.3 Storage conditions

Investigational product documentation must be maintained that includes all processes required to ensure drug is accurately administered. This includes documentation of drug storage, administration and, as applicable, storage temperatures, reconstitution, and use of required processes (e.g., required diluents, administration sets).

Infusion-related supplies (e.g., in-line filters, 0.9% NaCl solution, or pump) will not be supplied by the sponsor and are purchased locally by the center.

Please refer to the current versions of the IBs, SmPC or package inserts, and/or pharmacy manual for complete storage, handling, dispensing, and infusion/dosing information for nivolumab, and relatlimab.

## 7.4 Drug logistics and accountability

Study medication (nivolumab, relatlimab) will be provided by the manufacturer (BMS).

The investigator is responsible for ensuring that all clinical supplies received at the site are accounted for throughout the study. The study drug administered to the subject must be documented on the drug accountability form. The study drug will be supplied only to subjects participating in the study and in accordance with this protocol. The study drug may not be relabeled or reassigned for use by other subjects.

Unused study drug must be available for verification by the sponsor's study site monitor during on-site monitoring visits. When the study site is an authorized destruction unit and study drug supplies are destroyed on-site, this must also be documented on the drug return form. Potentially hazardous materials such as used ampules, needles, syringes, and vials containing hazardous liquids should be disposed of immediately in a safe manner and therefore will not be retained for drug accountability purposes. The immediate destruction of these drug supplies should be documented in the drug accountability records on site.

The responsible site personnel will confirm the receipt of study drug and will use the study drug only within the framework of this clinical study and in accordance with this protocol.

## 7.5 Assignment to treatment group

Patients will be allocated by randomization to Arm A and Arm B for up to 30 patients in each arm. All patients will be randomized during the screening visit using a central block randomization process. Randomization will be handled via eCRF.

## 7.6 Blinding

Not applicable

## 7.7 Study drug administration

### 7.7.1 Preparation and administration of nivolumab and relatlimab

Clinical supplies (nivolumab, relatlimab) will be prepared for use and labeled centrally by the manufacturer (BMS). For each individual patient and study treatment, “ready for use” medication will be shipped directly to the investigator or designee at the study site for immediate use.

All doses of study medication will be administered at the study site by a member of the investigators team. This person will control and document that the subject receives the treatment as planned.

Subjects randomized to the nivolumab/relatlimab arm should receive nivolumab 240 mg administered IV over 30 minutes followed by relatlimab 80 mg administered IV over 30.

Dosing calculations will not be necessary as flat dosing is planned. When study drugs (nivolumab and relatlimab) are to be infused on the same day, nivolumab is to be administered first. Nivolumab infusion must be promptly followed by a saline flush to clear the line of nivolumab before starting the relatlimab infusion. The second infusion will always be the relatlimab study drug and will start after the infusion line has been flushed, filters changed and the patient has been observed to ensure no infusion reaction has occurred. The time between infusions is expected to be approximately 30 minutes but may be more or less depending on the situation.

For this study, IMPs such as partially used study drug containers, vials and syringes may be destroyed on site. It is the investigator's or designee's responsibility to arrange for disposal, provided that procedures for proper disposal have been established according to applicable federal, state, local, and institutional guidelines and procedures, and provided that appropriate records of disposal are kept.

## 7.8 Dose modification guidelines

### 7.8.1 Dose modification guidelines for nivolumab or nivolumab and relatlimab therapy

#### 7.8.1.1 Dose delay or interruption criteria for nivolumab or nivolumab and relatlimab therapy

Subjects who experience the following must have all study drugs (nivolumab, relatlimab) held:

- Potential DLTs (per definition, are related to study drug) until DLT relatedness is defined.
- Select drug-related AEs and drug-related laboratory abnormalities:
  - ≥Grade 1 pneumonitis
  - Single grade increase shift from baseline (at least to grade 2) of AST, ALT and/or total bilirubin
  - ≥Grade 2 creatinine
  - ≥Grade 2 diarrhea or colitis
  - ≥Grade 2 neurological AE
  - Grade 4 amylase or lipase abnormality regardless of symptoms or clinical manifestations
- Any Grade ≥3 skin, drug-related AE

- Any Grade ≥2 non-skin, drug-related AE, with the following exception:
  - ≥Grade 2 drug-related fatigue does not require a treatment delay.
- Any Grade 3 drug-related laboratory abnormality (excluding AST, ALT or Total Bilirubin), with the following exceptions for lymphopenia:
  - Grade 3 lymphopenia does not require dose delay
- All troponin elevation upon relatlimab therapy requires dose delay and immediate cardiac evaluation including ECG and echocardiography
- Any AE, laboratory abnormality, or intercurrent illness which, in the judgment of the investigator, warrants delaying the dose of study medication.
- Any grade 3 or 4 infusion-related reaction

Subjects receiving relatlimab in combination with nivolumab who have drug-related toxicities that meet the criteria for dose delay should have both drugs (relatlimab and nivolumab) delayed until retreatment criteria are met (exceptions apply to the retreatment criteria after dose delay of relatlimab and nivolumab for Grade ≥3 amylase and lipase abnormalities that are not associated with symptoms or clinical manifestations of pancreatitis and that are attributed to relatlimab and/or nivolumab). Subjects who require delay of nivolumab and relatlimab should be re-evaluated weekly or more frequently if clinically indicated and resume nivolumab and/or relatlimab dosing when re-treatment criteria are met.

Subjects receiving ongoing treatment with relatlimab and nivolumab, treatment should be delayed for the following:

- Myocarditis (any grade). For Myocarditis Adverse Event Management Algorithm refer to the investigator´s brochure relatlimab
- All troponin elevations require a dose delay to allow for prompt cardiac evaluation (e.g., monitoring of cardiac troponin, troponin T or I. Following this evaluation, determination of further treatment will be based on discussion with the coordinating PI.

#### 7.8.1.2 Criteria for resuming treatment for nivolumab or relatlimab

In NEOpredict-Lung maximum duration of the preoperative treatment period is 42 days (6 weeks). Preoperative treatment is considered non-feasible in patients who cannot proceed to curative surgery within 43 days from first dose of study treatment. Accordingly, study treatment with nivolumab (Arm A) or nivolumab plus relatlimab (Arm B) may not be resumed in patients fulfilling criteria for treatment delays after cycle 1 that will result in a delay of surgery beyond day 43.

Participants who require delay or interruption of any study treatment should be re-evaluated weekly or more frequently if clinically indicated and resume dosing when re-treatment criteria are met. If the criteria to resume treatment are met, the subject should restart treatment at the next scheduled time point per protocol. Importantly, the total preoperative study treatment must not

be extended more than 6 weeks to ensure timely resection. However, in the absence of toxicities patients may proceed to surgery prior to day 43.

If a nivolumab-related infusion reaction prevents subsequent infusion of relatlimab on the same day, the dose of relatlimab should be replaced as soon as possible.

#### 7.8.1.3 Guidelines for permanent discontinuation of investigational drugs

Subjects meeting any of the following criteria will be required to permanently discontinue all investigational study drugs:

- Any Grade 2 drug-related uveitis, eye pain or blurred vision that does not respond to topical therapy and does not improve to Grade 1 severity within the re-treatment period or requires systemic treatment
- Any Grade 3 non-skin drug-related AE lasting >7days or recurs with the following exceptions for laboratory abnormalities, drug-related uveitis, pneumonitis, bronchospasm, hypersensitivity reactions, infusion reactions and endocrinopathies
- Grade 3 drug-related uveitis, pneumonitis, bronchospasm, hypersensitivity reactions, or infusion reaction of any duration requires discontinuation
- Grade 3 myocarditis
- Grade 3 drug-related endocrinopathies adequately controlled with only physiologic hormone replacement do not require discontinuation
- Grade 3 drug-related laboratory abnormalities do not require discontinuation except:
    - Grade 3 drug-related thrombocytopenia > 7days or associated with bleeding
    - Grade 3 drug-related AST, ALT or total bilirubin abnormalities require discontinuation
    - Concurrent AST or ALT > 3x ULN and total bilirubin > 2x ULN
    - Grade 3 elevation of troponin associated with any other sign of cardiac toxicity
- Any Grade 4 drug-related AE or laboratory abnormality (including but not limited to creatinine, AST, ALT or bilirubin), except for the following events which do not require discontinuation:
    - Grade 4 neutropenia ≤7days
    - Grade 4 lymphopenia or leukopenia
    - Isolated grade 4 amylase or lipase abnormalities that are not associated with symptoms or clinical manifestations of pancreatitis
    - Isolated grade 4 electrolyte imbalances / abnormalities that are not associated with clinical sequelae and are corrected with supplementation / appropriate management within 72 hours of their onset
- Grade 4 drug-related endocrinopathy AEs, such as adrenal insufficiency, ACTH deficiency, hyper- or hypothyroidism, or glucose intolerance, which resolves or are adequately controlled with physiologic hormone replacement (corticosteroids, thyroid hormones) or glucose-controlling agents may not require discontinuation after approval from the study sponsor/coordinating investigator

- Any event that leads to delay in a preoperative study period lasting > 6 weeks requires discontinuation
- Any AE, laboratory abnormality or intercurrent illness which, in the judgement of the investigator, presents a substantial clinical risk to the subject.

The consideration to re-initiate study therapy in selected cases at any time point after discontinuation could be made on a case by case basis after considering the overall benefit/risk profile and in consultation between the Investigator and the study sponsor/coordinating investigator. However, the preoperative study period must not be extended beyond 6 weeks.

If a subject meets the criteria for discontinuation of relatlimab but not nivolumab, treatment with nivolumab may not resume until the AE has fully resolved and the subject has discontinued steroids, if they were required for treatment of the AE. The relationship to relatlimab should be well documented in the source documents. If a subject in the nivolumab/relatlimab combination arm meets criteria for discontinuation and investigator is unable to determine whether the event is related to both or one study drug, the subject should discontinue both nivolumab and relatlimab and be taken off the treatment phase of the study.

#### 7.8.1.4 Dose reductions for nivolumab or nivolumab plus relatlimab therapy

There will be no dose escalations or reductions of nivolumab or relatlimab allowed.

#### 7.8.1.5 Management Algorithms for Immune Checkpoint Inhibitors

ICI agents are associated with AEs that can differ in severity and duration than AEs caused by other therapeutic classes. Nivolumab and relatlimab are considered ICI agents in this protocol. Early recognition and management of AEs associated with ICI agents may mitigate severe toxicity. Management algorithms have been developed to assist investigators in assessing and managing the following groups of AEs:

- Gastrointestinal
- Renal
- Pulmonary
- Hepatic
- Endocrinopathy
- Skin
- Neurological

The above algorithms are found in the nivolumab IB.

#### 7.8.1.6 Treatment of nivolumab- or relatlimab-related infusion reactions

Since nivolumab and relatlimab contain only human immunoglobulin protein sequences, they are unlikely to be immunogenic and induce infusion or hypersensitivity reactions. However, if such a reaction were to occur, it might manifest with fever, chills, rigors, headache, rash,

pruritus, arthralgias, hypotension, hypertension, bronchospasm, or other allergic-like reactions. All Grade 3 or 4 infusion reactions should be reported within 24 hours to the coordinating investigator and reported as an SAE if it meets the criteria. Infusion reactions should be graded according to NCI CTCAE version 4 guidelines.

Treatment recommendations are provided below and may be modified based on local treatment standards and guidelines, as appropriate:

**For Grade 1 symptoms**: (mild reaction; infusion interruption not indicated; intervention not indicated): Remain at bedside and monitor subject until recovery from symptoms. The following prophylactic premedications are recommended for future infusions: diphenhydramine 50 mg (or equivalent) and/or acetaminophen/paracetamol 325 to 1000 mg at least 30 minutes before additional nivolumab or relatlimab administrations.

**For Grade 2 symptoms**: (moderate reaction required therapy or infusion interruption but responds promptly to symptomatic treatment (eg, antihistamines, non-steroidal anti-inflammatory drugs, narcotics, corticosteroids, bronchodilators, IV fluids); prophylactic medications indicated for ≤ 24 hours):

- Stop the nivolumab or relatlimab infusion, begin an IV infusion of normal saline, and treat the subject with diphenhydramine 50 mg IV (or equivalent) and/or acetaminophen/paracetamol 325 to 1000 mg; remain at bedside and monitor subject until resolution of symptoms. Corticosteroid and/or bronchodilator therapy may also be administered as appropriate. If the infusion is interrupted, then restart the infusion at 50% of the original infusion rate when symptoms resolve; if no further complications ensue after 30 minutes, the rate may be increased to 100% of the original infusion rate. Monitor subject closely. If symptoms recur, then no further nivolumab or relatlimab will be administered at that visit.
- For future infusions, the following prophylactic premedications are recommended: diphenhydramine 50 mg (or equivalent) and/or acetaminophen/paracetamol 325 to 1000 mg should be administered at least 30 minutes before nivolumab or relatlimab infusions. If necessary, corticosteroids (up to 25 mg of hydrocortisone or equivalent) may be used.

**For Grades 3 or 4 symptoms**: (severe reaction, Grade 3: prolonged [ie, not rapidly responsive to symptomatic medication and/or brief interruption of infusion]; recurrence of symptoms following initial improvement; hospitalization indicated for other clinical sequelae (eg, renal impairment, pulmonary infiltrates). Grade 4: Life-threatening; pressor or ventilatory support indicated):

- Immediately discontinue infusion of nivolumab or relatlimab. Begin an IV infusion of normal saline and treat the subject as follows: Recommend bronchodilators, epinephrine 0.2 to 1 mg of a 1:1000 solution for subcutaneous administration or 0.1 to 0.25 mg of a 1:10,000 solution injected slowly for IV administration, and/or diphenhydramine 50 mg IV with methylprednisolone 100 mg IV (or equivalent), as needed. Subject should be monitored until the Investigator is comfortable that the symptoms will not recur. Nivolumab or relatlimab will be permanently discontinued. Investigators should follow their institutional guidelines for the treatment of anaphylaxis. Remain at bedside and monitor subject until recovery of the symptoms.

In case of late-occurring hypersensitivity symptoms (eg, appearance of a localized or generalized pruritus within 1 week after treatment), symptomatic treatment may be given (e.g., oral antihistamine or corticosteroids.

## 7.9 Previous and concomitant treatment

For restricted previous and/or concomitant treatment please refer to section 6.4, exclusion criteria.

In general, patients will be allowed to continue with any concomitant medication throughout the duration of this study in accordance with the treating physician´s best clinical judgment.

Standard of care, treatment of any pre-existing medical conditions and treatment of any emergent adverse drug reaction (ADR) will be provided to patients as per their treating physician´s best clinical judgment and according to the reference documents of the study medication.

However, the following treatments are prohibited during the study (unless utilized to treat a drug related adverse event):

- Immunosuppressive agents
- Immunosuppressive doses of systemic corticosteroids (> 10 mg daily prednisone equivalents) or other immunosuppressive medications within 14 days of study drug administration. Inhaled or topical steroids, and adrenal replacement doses > 10 mg daily prednisone equivalents are permitted in the absence of active autoimmune disease.
- Any concurrent anti-neoplastic therapy (i.e., chemotherapy, hormonal therapy, immunotherapy, radiotherapy)
- Botanical formulations with an approved indication for cancer treatment [e.g., traditional Chinese medicines], extensive, non-palliative radiation therapy, or standard or investigational agents for treatment of NSCLC.
- The application of live vaccine is prohibited within 30 days prior to the first dose of study medication. Patients also should not receive live vaccine while receiving study medication and up to 100 days after the last dose of study drug.

The administration of all types of pain killers according local guidelines is also permitted.

Since the study drugs nivolumab and relatlimab are of cardiotoxic potential the possible risk of any concomitant medication must be assessed. There is a range of cardiac and also non-cardiac medicines that may cause QT prolongation as an unwanted side effect (Appendix V).

**A list of restricted and excluded concomitant therapies is provided in Table 3.**

**Table 3**

**Table 3: Permitted and prohibited concomitant therapy (All study arms)**

| Therapy | As Needed | Chronic Use | Conditions for Use |
|---|---|---|---|
| Anticoagulants other than warfarin/ Phenprocoumon | yes | yes | • Careful evaluation is required if patients need to be administered anticoagulation during study treatment.<br>• Use of warfarin/phenprocoumon is prohibited. Refer to Inclusion Criterion "Adequate coagulation function". |
| Biologic response modifiers | no | no | |
| Other Chemotherapy | no | no | |
| Colony-stimulating factors | yes | yes | Follow local guidelines. |
| Erythroid growth factors | yes | no | Follow local guidelines. |
| Experimental medicines | no | no | |
| Investigational agents | no | no | |
| Nonsteroidal anti-inflammatory drugs | yes | yes* | *Chronic use of aspirin up to 325 mg/day is permitted before resection. |
| Radiotherapy | no | no | |

The use of analgesic agents during the conduct of the study is permitted at the discretion of the investigator. The chronic use of nonsteroidal anti-inflammatory drugs with a high risk of bleeding (for example, indomethacin, ibuprofen, naproxen, or similar agents) is strongly discouraged before the resection except at the discretion and responsibility of the investigator after careful assessment of the individual bleeding risk of the patient. Chronic use of aspirin up to 325 mg/day is permitted before the resection. After the resection there are no restrictions for the use of nonsteroidal anti-inflammatory drugs.

Chronic use of analgesic agents with no or low bleeding risk (for example,

paracetamol/acetaminophen, dipyrone/metamizole, or propyphenazone) is acceptable during the trial.

### 7.10 Post-study therapy

Treatment duration within the framework of this study is for a maximum of two preoperative cycles of study therapy. All subsequent therapies including but not restricted to surgery, adjuvant chemotherapy, adjuvant radiotherapy will be administered following institutional guidelines and standard of care for NSCLC.

# 8 Procedures and variables

## 8.1 Tabulated schedules

Specific time points for evaluation of efficacy variables and safety measures are given in Table 4.

**Table 4: Schedule of assessments**

**Arm A Nivolumab**

| Assessment | Screening | Study treatment | | | SoC Surgery | Post SoC Surgery | Follow-up | EOS-Visit |
|---|---|---|---|---|---|---|---|---|
| | -28 to 0 | W1 1 | W3 15 | pre-OP | Anatomic resection ≤ 43 | 1-7 after surgery | 12 months 1,2,3, 6, 9 and 12 months after surgery (±5 days) | In case of premature end of trial / subject withdrawal / relapse |
| Signed Informed Consent | X | | | | | | | |
| Additional consent (biobanking, translational research) (if applicable) | X | | | | | | | |
| Medical history | X | | | | | | | |
| Demography, including baseline characteristics | X | | | | | | | |

| Assessment | Screening | Study treatment | | | SoC Surgery | Post SoC Surgery | Follow-up | EOS-Visit |
|---|---|---|---|---|---|---|---|---|
| | -28 to 0 | W1 1 | W3 15 | pre-OP | Anatomic resection ≤ 43 | 1-7 after surgery | 12 months 1,2,3, 6, 9 and 12 months after surgery (±5 days) | In case of premature end of trial / subject withdrawal / relapse |
| Histology/cytology[1] | X | | | | X | | | |
| Tumor assessment by CT/MRI/PET-CT/MRI[2] | X[2] | | | X[2] | | | X[2] | |
| Mediastinal staging confirmed by PET-CT/MRI/EBUS-TBNA/mediastinoscopy[3] | X[3] | | | | | | | |
| Physical examination | X | X | X | X | | X | X | |
| Weight | X | X | X | X | | X | X | |
| ECOG PS | X | X | X | X | | X | X | X |
| ECG (within 14 d prior to first study drug administration) | X | as clinically indicated | | | | | as clinically indicated | |
| Oxygen saturation (within 14 d prior to first administration of study drug) | X | as clinically indicated | | | | | | |
| Lung function testing | X | as clinically indicated | | | | | | |
| Echocardiography[4] | X | as clinically indicated | | | | | | |
| Concomitant medication | X | X | X | | | X | X | X |
| Nivolumab infusion | | X | X | | | | | |
| Adverse events/Toxicity | X | X | X | X | | X | X | X |
| Survival status | X | X | X | X | | X | X | X |
| SoC treatment of adjuvant / recurrent disease | | | | | | X | X | X |

**Laboratory assessments**

| Assessment | Screening | Study treatment | | | SoC Surgery | Post SoC Surgery | Follow-up | EOS-Visit |
|---|---|---|---|---|---|---|---|---|
| | -28 to 0 | W1 1 | W3 15 | pre-OP | Anatomic resection ≤ 43 | 1-7 after surgery | 12 months 1,2,3, 6, 9 and 12 months after surgery (±5 days) | In case of premature end of trial / subject withdrawal / relapse |
| Serum or urine pregnancy testing β-HCG (within 24h prior to first study drug administration) | X[9] | | | X[9] | | | X[9] | X[9] |
| Serological test for HIV-1, HIV-2, HCV, HBV | X | | | | | | | |
| Clinical chemistry[5] | X | X | X | X | | X | X | X |
| Hematology[6] | X | X | X | X | | X | X | X |
| TSH, fT3, fT4 | X | X | | X | | X | X | X |
| Cardiac Troponin I or T (within 14 d prior to first study drug administration) | X | | | | | | | |
| Urine analysis | X | as clinically indicated | | | | | | |
| | | | | | | | | |
| **Translational research** | | | | | | | | |
| FFPE tumor biopsy (lymph node samples if available) | X | | | | X | | | |
| FF tumor and lymph node samples | | | | | X | | | |
| Trisodium citrate blood (Flow cytometry) 50 ml[7] | | X | X | X | - | X | X[10] | X |
| EDTA-blood for HLA-typing 6 ml | X | | | | | | | |
| Serum blood 10 ml | X | X | X | X | | X | X[10] | X |
| EDTA Plasma blood 20 ml | X | | X | X | | X | X[10] | X |
| Exhaled volatile organic substances (eNose)[8] | X | | | X | | | | ´X |

CT=computer tomography; ECG=electrocardiogram; EBUS-TBNA=endobronchial ultrasound-guided transbronchial needle biopsy; ECOG-PS=Eastern Cooperative Oncology Group Performance Status; FF= Freshly frozen; FFPE= Formalin-fixed paraffin-embedded; HBV=hepatitis B virus; HCV=hepatitis C virus; HIV=human immunodeficiency virus; MRI=magnetic resonance imaging; PET=Positron-emission tomography; SoC=Standard of care; TSH=Thyroid-stimulating hormone

[1]Histologically/cytologically proven non-small cell lung cancer

[2]Standard of care imaging studies (CT, MRI, bone scan, PET-CT/MRI) of primary tumor localization and to exclude metastatic disease for screening; preoperative imaging (CT, MRI) if clinically indicated; after surgery imaging studies (CT, MRI) as recommended by current guidelines (e.g. every 3 months for one year and every 6 months in the second year) or if clinically indicated. During the 12 months follow-up period, imaging studies are only performed in visits at month 3, 6, 9, 12.

[3]Mediastinal staging as recommended by current local guidelines using PET-CT/MRI, EBUS-TBNA and/or staging mediastinoscopy.

[4]Within 6 months before first administration of study drug (may be replaced by MUGA scan at non-German sites).

[5]Sodium, potassium, chloride, calcium, magnesium, creatinine, blood urea nitrogen, GFR, uric acid, bilirubin, AST, ALT, LDH, alkaline phosphatase, total protein, albumin, TPT, PT-INR, pTT, CRP.

[6]Hemoglobin, hematocrit, platelet count, white blood count including differential blood count.

[7]Blood to be drawn prior to infusion of study medication (days 1 and 15), prior to surgery, and prior to first infusion of SoC adjuvant chemotherapy, respectively.

[8]Direct measurements using eNose device.

[9]Pregnancy testing should be done in all WOCBP at least until day 165 (approximately 24 weeks) post treatment

[10]Samples for Translational research at visits 3, 6, 9 and 12 months only

## Arm B Nivolumab / Relatlimab

| Assessment | Screening | Study treatment | | | SoC Surgery | Post SoC Surgery | Follow-up | EOS |
|---|---|---|---|---|---|---|---|---|
| | -28 to 0 | W1 1 | W3 15 | pre-OP | Anatomic resection ≤ 43 | 1-7 after surgery | 12 months 1,2,3, 6, 9 and 12 months after surgery (±5 days) | In case of premature end of trial / subject withdrawal / relapse |
| Signed Informed Consent | X | | | | | | | |
| Additional consent (biobanking, translational research) (if applicable) | X | | | | | | | |
| Medical history | X | | | | | | | |
| Demography, including baseline characteristics | X | | | | | | | |
| Histology/cytology[1] | X | | | | X | | | |
| Tumor assessment by CT/MRI/PET-CT/MRI[2] | X[2] | | | X[2] | | | X[2] | X[2] |
| Mediastinal staging confirmed by PET-CT/MRI/EBUS-TBNA/mediastinoscopy[3] | X[3] | | | | | | | |

| Assessment | Screening | Study treatment | | | SoC Surgery | Post SoC Surgery | Follow-up | EOS |
|---|---|---|---|---|---|---|---|---|
| | -28 to 0 | W1 1 | W3 15 | pre-OP | Anatomic resection ≤ 43 | 1-7 after surgery | 12 months 1,2,3, 6, 9 and 12 months after surgery (±5 days) | In case of premature end of trial / subject withdrawal / relapse |
| Physical examination | X | X | X | X | | X | X | |
| Weight | X | X | X | X | | X | X | |
| ECOG PS | X | X | X | X | | X | X | X |
| ECG (within 14d prior to first study drug administration) | X | X | X | X | | as clinically indicated | | |
| Oxygen saturation (within 14 d prior to first administration of study drug) | X | as clinically indicated | | | | | | |
| Lung function testing | X | as clinically indicated | | | | | | |
| Echocardiography with LVEF assessment[4] | X | as clinically indicated | | | | | | |
| Concomitant medication | X | X | X | | | X | X | X |
| Nivolumab infusion | | X | X | | | | | |
| Relatlimab infusion (within 30 min after nivolumab) | | X | X | | | | | |
| Adverse events/Toxicity | X | X | X | X | | X | X | X |
| Survival status | X | X | X | X | | X | X | X |
| SoC treatment of adjuvant / recurrent disease | | | | | | X | X | X |

| **Laboratory assessments** | | | | | | | | |
|---|---|---|---|---|---|---|---|---|
| Serum or urine pregnancy testing β-HCG (within 24h prior to first study drug administration) | X[9] | | | X[9] | | | X[9] | X[9] |
| Serological test for HIV-1, HIV-2, HCV, HBV | X | | | | | | | |
| Clinical chemistry[5] | X | X | X | X | | X | X | X |
| Hematology[6] | X | X | X | X | | X | X | X |

| Assessment | Screening | Study treatment | | | SoC Surgery | Post SoC Surgery | Follow-up | EOS |
|---|---|---|---|---|---|---|---|---|
| | -28 to 0 | W1 1 | W3 15 | pre-OP | Anatomic resection ≤ 43 | 1-7 after surgery | 12 months 1,2,3, 6, 9 and 12 months after surgery (±5 days) | In case of premature end of trial / subject withdrawal / relapse |
| TSH, fT3, fT4 | X | X | | X | | X | X | X |
| Cardiac Troponin I or T (within 14 d prior to first study drug administration) | X | X | X | X | | X | | |
| Urine analysis | X | as clinically indicated | | | | | | |
| | | | | | | | | |
| **Translational research** | | | | | | | | |
| FFPE tumor biopsy (lymph node samples if available) | X | | | | X | | | |
| FF tumor and lymph node samples | | | | | X | | | |
| Trisodium citrate blood (Flow cytometry) 50 ml[7] | | X | X | X | | X | X[10] | X |
| EDTA-blood for HLA-typing 6 ml | X | | | | | | | |
| Serum blood 10 ml | X | X | X | X | | X | X[10] | X |
| EDTA Plasma blood 20 ml | X | | X | X | | X | X[10] | X |
| Exhaled volatile organic substances (eNose)[8] | X | | | X | | | | X |

CT=computer tomography; ECG=electrocardiogram; EBUS-TBNA=endobronchial ultrasound-guided transbronchial needle biopsy; ECOG-PS=Eastern Cooperative Oncology Group Performance Status; FF= Freshly frozen; FFPE= Formalin-fixed paraffin-embedded; HBV=hepatitis B virus; HCV=hepatitis C virus; HIV=human immunodeficiency virus; MRI=magnetic resonance imaging; PET=Positron-emission tomography; SoC=Standard of care; TSH=Thyroid-stimulating hormone

[1]Histologically/cytologically proven non-small cell lung cancer

[2]Standard of care imaging studies (CT, MRI, bone scan, PET-CT/MRI) of primary tumor localization and to exclude metastatic disease for screening; preoperative imaging (CT, MRI) if clinically indicated; after surgery imaging studies (CT, MRI) as recommended by current guidelines (e.g. every 3 months for one year and every 6 months in the second year) or if clinically indicated. During the 12 months follow-up period, imaging studies are only performed in visits at month 3, 6, 9, 12.

[3]Mediastinal staging as recommended by current local guidelines using PET-CT/MRI, EBUS-TBNA and/or staging mediastinoscopy.

[4]Mandatory screening examination within 6 months before first administration of study drug (may be replaced by MUGA scan at non-German sites).

[5]Sodium, potassium, chloride, calcium, magnesium, creatinine, blood urea nitrogen, GFR, uric acid, bilirubin, AST, ALT, LDH,

alkaline phosphatase, total protein, albumin, TPT, PT-INR, pTT, CRP.

[6]Hemoglobin, hematocrit, platelet count, white blood count including differential blood count.

[7]Blood to be drawn prior to infusion of study medication (days 1 and 15), prior to surgery, and prior to first infusion of SoC adjuvant chemotherapy, respectively.

[8]Direct measurements using eNose device.

[9]Pregnancy testing should be done in all WOCBP at least until day 165 (approximately 24 weeks) post treatment

[10] Samples for Translational research at visits 3, 6, 9 and 12 months only

A time window of +/- 2 days is allowed for each visit, time deviations more than 2 days will be documented as protocol deviations, if applicable.

If not stated otherwise, the measures listed in the following sections will be performed by or under the supervision of an investigator.

### 8.1.1 Translational studies

Systematic sample acquisition and biobanking is integral part of the study NEOpredict-Lung. The study-associated translational program is described in a separate protocol. In brief, the following translational studies are assessed:

- Genomic profiling for determination of tumor mutational burden (TMB), HLA status, HLA loss, and neoantigens
- Phenotyping, T cell receptor sequencing, and functional ex vivo studies of tumor-infiltrating lymphocytes from resected tumors
- Phenotyping and ex vivo analyses of myeloid cells from resected tumors and peripheral blood
- Multiplexed profiling of RNA and protein expression in surplus material from pretreatment tumor biopsies and resected tumors by Digital Spatial Profiling
- Monitoring of metabolic changes induced by study therapy by quantitative LC/MS in sequentially acquired plasma samples
- Monitoring the composition of exhaled volatile organic substances using the eNose device (www.enose.nl)
- Monitoring of dynamic changes of tumor lesions in response to study therapy by functional imaging using MRI and PET technology

## 8.2 Description of visits

### 8.2.1 Screening (Days -28 to Day 0)

A subject information session will be held. Considering site-specific logistic requirements, the investigator may decide upon timing of the screening examinations (e.g. starting immediately after the informed consent procedure at the earliest; possible split-up into more than one visit). If the (first) screening procedures already start on the same day (same date) as the subject signs the informed consent form, the time of the subject’s signature must be recorded in the source documents.

**Note: No screening procedures may be performed unless written informed consent has been obtained. In case laboratory or imaging procedures were performed for clinical reasons prior to signing informed consent, these can be used for screening purposes with consent of the patient. However, all screening laboratory and imaging results must have been obtained within 28 days of randomization (LVEF assessment within 6 months).**

Within up -28 d prior to the first study drug administration the following measures / actions will be performed:

- Signing of Informed Consent Form
- Signing additional Informed Consent Forms (biobanking, translational research), if applicable
- Medical History
- Demography (including baseline characteristics)
- Histology / cytology
- Tumor assessment and mediastinal staging
- Physical examination
- Weight
- ECOG PS
- Lung function testing
- Echocardiography for LVEF assessment (may be replaced by MUGA scan at non-German sites), within 6 months before first administration of study drug
- Recommendation of curative surgery by the interdisciplinary thoracic oncology tumor board
- Concomitant medication
- Adverse events/toxicity
- Survival status
- Serum or urine pregnancy test (within 24 hours prior to study drug administration) in WOCBP
- Serological test for HBV, HCV
- Serological test for HIV-1 and -2
- Clinical chemistry
- Hematology
- Thyroid-stimulating hormone (TSH), free triiodothyronine (fT)3, fT4
- Urine analysis
- Tumor and blood samples for translational endpoints

Within up to two weeks (-14 d) prior to the first study drug administration the following measures / actions will be performed:

- Electrocardiogram (ECG)
- Oxygen saturation (Collected at rest and after mild exertion via pulse oximetry to

establish baseline. If subject has oxygen saturation <90%, consult the coordinating investigator prior to enrollment.

- Cardiac troponin I or T

### 8.2.2 Allocation to treatment

Patients eligible for study participation will be allocated a treatment number. Allocation of a treatment number might be performed at the screening visit or between Screening and Visit 1 providing that all results of the screening assessments are available and eligibility criteria checked before.

### 8.2.3 Treatment visits W1 and W3

At Visits W1, W3 and preoperative visit the following actions / measures will be done:

- Physical examination
- Weight
- ECOG PS
- ECG (mandatory in Arm B: nivolumab/relatlimab; as clinically indicated in Arm A)
- Echocardiography (as clinically indicated)
- Lung function testing (as clinically indicated)
- Oxygen saturation (as clinically indicated)
- Concomitant medication
- Adverse events/toxicity
- Survival status
- Clinical chemistry
- Hematology
- TSH, fT3, fT4 (visit W1 only)
- Urine analysis (as clinically indicated)
- Cardiac Troponin I or T (only in Arm B: nivolumab/relatlimab)
- Blood samples for translational studies
- Administration of study drug(s), see Section 7.7

### 8.2.4 Visit pre-OP

- Tumor assessment by CT, PET/CT or PET/MRI (if clinically indicated)

- Physical examination
- Weight
- ECOG PS
- ECG (as clinically indicated; mandatory in Arm B: nivolumab/relatlimab)
- Echocardiography (as clinically indicated)
- Echocardiography (as clinically indicated)
- Lung function testing (as clinically indicated)
- Oxygen saturation (as clinically indicated)
- Concomitant medication
- Adverse events/toxicity
- Survival status
- Clinical chemistry
- Hematology
- Thyroid-stimulating hormone (TSH), free triiodothyronine (fT)3, fT4
- Pregnancy test in WOCBP
- Urine analysis (as clinical indicated)
- Cardiac Troponin I or T (only in Arm B: nivolumab/relatlimab)
- Blood samples for translational studies

### 8.2.5 Visit post-OP (D1 to 7 after surgery)

- Physical examination
- Weight
- ECOG PS
- Concomitant medication
- Adverse events/toxicity
- Survival status
- Clinical chemistry
- Hematology
- Cardiac Troponin I or T (only in Arm B: nivolumab/relatlimab)
- Thyroid-stimulating hormone (TSH), free triiodothyronine (fT)3, fT4
- Urine analysis (as clinically indicated)

- Tumor samples for translational studies (taken during surgery)
- Blood samples for translational studies

### 8.2.6 Follow up visits (1, 2 and 3 months after surgery and every 3 months thereafter for up to 12 months after surgery) (± 5 days)

- Tumor assessment (at 3, 6, 9 and 12 months after surgery, per clinical guidelines)
- Physical examination
- Weight
- ECOG PS
- ECG (as clinical indicated)
- Echocardiography (as clinically indicated)
- Lung function testing (as clinically indicated)
- Oxygen saturation (as clinically indicated)
- Concomitant medication
- Adverse events/toxicity
- Survival status
- Clinical chemistry
- Hematology
- Urine analysis (as clinical indicated)
- Thyroid-stimulating hormone (TSH), free triiodothyronine (fT)3, fT4
- Pregnancy test in WOCBP at least until day 165 (approximately 24 weeks) post treatment
- Tumor sample for translational studies (relapse visit only, if surplus tissue from clinically indicated diagnostic or therapeutic procedure is available and patient provides consent)
- Blood samples for translational studies (visits 1, 3, 6, 9 and 12 months post surgery)

### 8.2.7 End of study visit

End of the clinical trial is the date on which the last patient has her or his last visit (12 months Follow Up Visit (LPLV). In the event a patient discontinues prematurely from the study (relapse, decision by the patient and/or the investigator) or if the study is prematurely terminated the following measures / actions will be performed within approximately 28 days:

- ECOG PS
- ECG (as clinically indicated)

- Echocardiography (as clinically indicated)
- Adverse events/toxicity
- Concomitant medication
- Survival status
- Treatment for recurrent disease (if applicable)
- Clinical chemistry
- Hematology
- Pregnancy test in WOCBP at least until day 165 (approximately 24 weeks) post study treatment
- Thyroid-stimulating hormone (TSH), free triiodothyronine (fT)3, fT4
- Blood samples for translational studies

## 8.3 Laboratory assessments

For laboratory assessment, the following assessments will be done:

**Table 5: Laboratory parameters**

| |
|---|
| **Hematology:** Hemoglobin, hematocrit, platelet count, white blood count including differential blood count |
| **Clinical chemistry:** sodium, potassium, chloride, calcium, magnesium, creatinine, blood urea nitrogen, glomerular filtration rate (GFR), uric acid, bilirubin, AST, ALT, lactate dehydrogenase (LDH), alkaline phosphatase, total protein, albumin, thromboplastin time, PT-INR, PTT, C reactive protein (CRP) and TSH, fT3, fT4, troponin |
| **Serology (only at screening):** hepatitis B virus surface antigen, hepatitis C virus antibodies, human immunodeficiency virus 1 and 2 antigen/antibody (if required by local standards) |
| **Translational parameters:** tumor sample, flow cytometry, HLA-typing, metabolic profiling, circulating nucleic acids |

## 8.4 Demographic characteristics

For demographic assessment, the following parameters will be recorded: Year of birth / age, sex, race / ethnicity, body weight, height, and body mass index (BMI).

## 8.5 Evaluation of clinical endpoints

Primary endpoint is the fesibility of each treatment arm as determined by the number of patients undergoing surgery within 43 days after first administration of study therapy.

Study evaluations will take place in accordance with the flow chart (Table 4). Baseline tumor assessments should be performed within 28 days prior to randomization, utilizing CT, MRI, PET/CT or PET/MRI as clinically indicated. Subsequent assessments should include all sites that were assessed at baseline and should use the same imaging method as was used at baseline. Changes in tumor measurements detected by preoperative imaging studies are assessed per RECIST 1.1 criteria. Subjects will be evaluated for disease progression within standard of care (e.g., every 3 months after surgery for 12 months). Disease-free survival is defined as the time from surgery to recurrence of a tumor lesion (RECIST 1.1).

**Definition of FDG-PET response** (Only applies to patients in which repeat FDG-PET imaging is performed): SUV-decrease ≥ 35%.

**Definition of PET non-response** (Only applies to patients in which repeat FDG-PET imaging is performed): SUV-decrease < 35%

**Definition of histopathological response**: Complete pathological response is defined as absence of viable tumor cells on routine hematoxylin and eosin staining of resected tumors and lymph nodes; major pathological response is defined as 10% or less viable tumor cells on routine hematoxylin and eosin staining of resected tumors; minor pathological response is defined as 50% to 10% viable tumor cells on routine hematoxylin and eosin staining of resected tumors.

**Overall survival** (OS) will be defined as the time from start of study treatment to death from any cause. Time to last observation will be used if a patient has not died or is lost to follow-up and OS for the patient will be considered censored.

**Disease-free survival** (DFS) will be defined as the time from surgery to recurrence or death for all patients with R0-resection. Time to last observation will be used if a patient is disease-free or has not died or is lost to follow-up and DFS for the patient will be considered censored.

## 8.6 Safety examinations

The following safety examinations will be performed at the time points specified in the study flowchart, see also section 8.1.

Physical examination

The physical examination will be performed by a physician at the study site including at least the organs of the cardiovascular, respiratory, and abdominal system. Orientating tests of neurological function will also be performed. Abnormal physical examination findings are recorded either as medical history or as AEs.

Body weight, height, and BMI

Body weight will be measured by a member of the investigator’s team with the subject in light clothes and without shoes after having emptied his/her bladder. The subject’s height will be measured (without shoes) to calculate the BMI.

Electrocardiogram

A complete standard 12-lead ECG will be recorded with a computerized ECG device by a member of the investigator’s team after the patient has rested in supine position for at least 15 min.

Echocardiography

A transthoracic echocardiography within 6 months before study drug administration is recommended to assess the left ventricular ejection fraction (LVEF). In non-German sites echocardiography may be replaced by MUGA scan.

Oxygen Saturation

If subject has an oxygen saturation <90%, the coordinating investigator should be consulted prior to enrollment.

ECOG Performance Status

ECOG Performance Status will be performed at each visit.

Computer tomography or magnetic resonance imaging combined with positron emission tomography (PET) using fluordeoxyglucose tracer (as clinically indicated)

A CT or MRI (with or without PET) of primary tumor-localization and abdomen/thorax will be performed at screening to exclude metastatic disease. A second imaging study (using the same anatomical imaging modality that was used for base line studies) will be conducted at the preoperative visit. After surgery imaging studies will be conducted as part of standard of care follow up.

Pregnancy test

Serum or urine pregnancy testing β-HCG with a sensitivity of at least 25 mIU/mL is to be done not more than 24 hours prior to the first study drug administration in female patients with childbearing potential (WOCBP). Additional pregnancy testing is done in WOCBP at the Visit pre-OP and at Follow-up visits or premature End of study visits at least until 165 days post first study treatment.

In each case of delayed menstrual period (over one month between menstruations) confirmation of absence of pregnancy is required. This requirement also applies to women of childbearing potential with infrequent or irregular menstrual cycles. In such a case pregnancy testing is recommended once a week.

Laboratory examinations

Blood and urine samples will be collected by a member of the investigator’s team. For time points see Section 8.1.

All laboratory examinations performed in this study are standard measures and will be performed by the local laboratories. Results of laboratory tests will be available and reviewed by

the investigator prior to study drug administration.

# 9 Response criteria

Efficacy assessments are scheduled to occur as indicated in Table 4. Response will be evaluated in this study using the proposed response criteria in Section 8.5.

# 10 Safety

## 10.1 Definitions of adverse events and adverse drug reactions

### 10.1.1 Classification of adverse events

An adverse event (AE) is any untoward medical occurrence (i.e. any unfavorable and unintended sign [including abnormal laboratory findings], symptom or disease) in a patient or clinical investigation subject after providing written informed consent for participation in the study. Therefore, an AE may or may not be temporally or causally associated with the use of a medicinal (investigational) product.

A surgical procedure that was planned prior to the start of the study by any physician treating the subject should not be recorded as AE.

The different kinds of adverse events and the way they have to be reported and graded are summarized the following subsections and in Appendix I. Any SAE submitted to the sponsor shall only contain anonymous or key coded information.

In the following differentiation between medical history and AEs, the term “condition” may include abnormal e.g. physical examination findings, symptoms, diseases, or laboratory or ECG test results.

- Conditions that started before signing of informed consent and for which no symptoms or treatment are present until signing of informed consent are recorded as medical history.
- Conditions that started before signing of informed consent and for which symptoms or treatment are present after signing of informed consent, at unchanged intensity, are recorded as medical history.
- Conditions that started or worsened after signing of informed consent will be documented as adverse events.

### 10.1.2 Serious adverse events (SAEs)

A serious adverse event (SAE) or serious adverse drug reaction (SADR) is classified as any untoward medical occurrence that at any dose (including overdose) meets the following criteria:

- Results in death
- Is life-threatening
- Requires inpatient hospitalization or prolongation of existing hospitalization
- Results in persistent or significant disability or incapacity
- Is a congenital anomaly or birth defect
- Suspected transmission of an infectious agent (e.g., pathogenic or nonpathogenic) via the study drug
- Overdose: An overdose is defined as the accidental or intentional administration of any dose of a product that is considered both excessive and medically important. All occurrences of overdose must be reported as an SAE.
- Is an important medical event as judged by the investigator
- Although pregnancy, overdose, potential drug-induced liver injury (DILI), and cancer are not always serious by regulatory definition, these events must be handled as SAEs.

### 10.1.3 Adverse drug reactions and serious adverse drug reactions

An adverse drug reaction is any noxious and unintended response to an IMP related to any dose with at least a reasonably possible causal relationship with the IMP.

Toxicity will be scored using common toxicity criteria for adverse events (CTCAE) Version 4.0 for toxicity and adverse event reporting.

All appropriate treatment areas have access to a copy of the CTCAE Version 4.0 in the investigator site file or as an electronic document provided by the sponsor. All serious adverse clinical experiences, whether observed by the investigator or reported by the patient, must be recorded, with details about the duration and intensity of each episode, the action taken with respect to the test drug, and the patient’s outcome in a separate SAE-document. The investigator must evaluate each adverse event for its relationship to the test drug and for its seriousness.

The investigator must assess all abnormal laboratory results for their clinical significance. If any abnormal laboratory result is considered clinically significant, the investigator must provide details about the action taken with respect to the test drug and about the event´s outcome. All laboratory values have to be signed by the investigator. All clinical significant abnormal laboratory results must be reported as AE.

### 10.1.4 Unexpected adverse drug reaction

An unexpected ADR is an ADR for which the nature or severity is not consistent with the applicable product information available for the IMP. Expected ADRs are listed in the appropriate reference documents (IBs for nivolumab and relatlimab). An ADR/suspected adverse reaction (SAR) is suspected if the causal relationship with study medication is expected. Events assessed as no causal relationship to the IMP are not suspected ADRs/SARs.

### 10.1.5 SUSAR - Suspected unexpected serious adverse reactions

A suspected unexpected serious adverse reaction (SUSAR) is an adverse event for which the nature or severity is not consistent with the product information available for the IMP, is regarded as serious, and has at least a possible causal relationship with the IMP.

### 10.1.6 Events of special interest

**Potential Drug Induced Liver Injury (DILI)**
Wherever possible, timely confirmation of initial liver-related laboratory abnormalities should occur prior to the reporting of a potential DILI event. All occurrences of potential DILIs, meeting the defined criteria, must be reported as SAEs (see Section 10.2 for reporting details).

Potential drug induced liver injury is defined as:

- AT (ALT or AST) elevation > 3 times upper limit of normal (ULN),
  AND
- Total bilirubin > 2 times ULN, without initial findings of cholestasis (elevated serum alkaline phosphatase),
  AND
- No other immediately apparent possible causes of AT elevation and hyperbilirubinemia, including, but not limited to, viral hepatitis, pre-existing chronic or acute liver disease, or the administration of other drug(s) known to be hepatotoxic.

For relatlimab the following drug-related events will be considered a hepatic AESI

- ALT or AST <8x ULN regardless of duration or
- ALT or AST >5x and ≤8x ULN, that fails to return to ≤ Grade 1 within 2 weeks despite medical intervention or
- Total bilirubin >5xULN, or
- Potential drug-induced liver injury (DILI) event (see above)

Any of the following drug-related events will be considered a non-hematologic AESI:

- Grade 2 immune-related eye pain or reduction in visual acuity that requires systemic treatment or

Grade 2 eye pain or reduction in visual acuity that does not respond to topical treatment and that does not improve to Grade 1 within 2 weeks of initiation of topical therapy.

Any of the following drug-related events will be considered a hematologic AESI:

- Grade 4 febrile neutropenia
- Grade 4 neutropenia that lasts > 5 days
- Grade 4 thrombocytopenia
- Grade 4 anemia
- Grade 3 thrombocytopenia associated with clinically significant bleeding
- Grade 3 febrile neutropenia that lasts >48 hrs
- Grade 3 hemolysis

Immune-mediated adverse events (IMAEs) are AEs consistent with an immune-mediated mechanism or immune-mediated component for which non-inflammatory etiologies (e.g.,

infection or tumor progression) have been ruled out. IMAEs can include events with an alternate etiology which were exacerbated by the induction of autoimmunity. Information supporting the assessment will be collected on the subject's case report form.

## 10.2 Documentation and follow-up of adverse events and serious adverse events

The sponsor ensures that all persons involved in the treatment of study subjects are adequately informed about the responsibilities and actions required when AEs occur. Study subjects will be asked at each visit whether they have experienced AEs or SAEs. AEs will be documented in the study subject's medical records and in the electronic case report form (eCRF).

All serious adverse event reports sent to the sponsor must include:

- Patient number / enrolment number
- Age
- Sex
- Severity of reaction (Grade 1-5 according to CTCAE criteria)
- Relationship to the study drug(s)
- Date and time of administration of study medications and all concomitant medications
- Medical treatment provided
- Outcome

SAEs and other events should be reported in English to the sponsor mentioning the protocol number and using the Protocol SAE Form (and the Pregnancy Surveillance Form for pregnancy reports). The investigator is responsible for evaluating all adverse events to determine whether criteria for "serious" as defined above are present. AEs observed, mentioned upon open questioning by a member of the investigator's team or spontaneously reported by the subject will be documented. The observation phase for AEs will start with signing the informed consent form and will end 28 days after end of study but at least 100 days after last intake of study drug, unless the investigator suspects a delayed adverse reaction to the study drug. In case of ongoing AEs after the last follow-up visit – especially when related to treatment with the study medication – the respective AE will be followed until resolution, if possible. SAEs will be reported up 28 days after end of study but at least to100 days after the last intake of study drug.

The following events must also be submitted as SAEs:

- Cancer
- Overdose
- Pregnancy
- Potential drug induced liver injury

### 10.2.1 SAE documentation responsibilities

Any SAE submitted to the sponsor shall only contain anonymous or key coded information.

**Table 6: Overview of AE reporting requirements**

| Event | Relationship to study drug | Reporting | Timeline |
|---|---|---|---|
| AE (Adverse Event) | Not relevant for AE classification, should be evaluated in the (e)CRF and in source documents | Investigator has to document all AEs in the (e)CRF and the patient medical records. Reporting of AE becomes mandatory immediately after the ICF is signed. | While completing the (e)CRF |
| SAE (Serious Adverse Event) | Not relevant for SAE classification, should be evaluated in (e)CRF and SAE report | Investigator sends SAE report to sponsor<br>All SAEs need to be collected and reported after signature of the informed consent | Immediately but not later than 24 h after awareness<br>SAE observation will end 28 days after end of study or 100 days after the last study drug administration |
| SAR (Serious Adverse Reaction) | Yes<br>- relationship evaluated to be at least possible | Investigator sends SAR report to sponsor<br>Re-assessment of the expectedness by sponsor delegate<br>Sponsor sends annual report to competent authorities and Ethics committee (EC)/IRB | Within 24 h after awareness<br>Once a year or on demand |
| SUSAR (Suspected Unexpected Serious Adverse | Yes<br>- relationship evaluated independently by the investigator and the sponsor | Sponsor reports each case report to competent authorities and EC/IRB and informs all | Sponsor reports within 15 days<br>Within 7 days if |

| Event | Relationship to study drug | Reporting | Timeline |
|---|---|---|---|
| Reaction) | / sponsor´s delegate. If either of them evaluates the relationship to study drug as possible and SAE is assessed as unexpected according to the Reference Safety Information SAE becomes expeditedly reportable | investigators/study sites | event is fatal or life-threatening |
| Pregnancy | N/A | Investigator sends pregnancy reporting form to sponsor | Immediately but not later than 24 h after awareness |

### 10.2.2 Collection and Reporting of AE/SAEs

Adverse events will be documented after providing written informed consent. This includes AEs that occurred after written informed consent, or were present prior to this and increased in severity, changed from being not suspected to being suspected to be due to study drug or any protocol specified procedure, or developed into an SAE after the start of the treatment period.

Each AE will be documented once according to the date of onset. If the AE onset was prior to the first dose of study drug and the event does not increase in severity after initiation of study drug, the AE is then considered to be a pre-treatment adverse event and will not be reported in the treatment-emergent adverse event incidence tables. If the onset is prior to the first dose of study drug and the severity increases thereafter, the event is documented as a treatment-emergent AE. An AE with onset after the first dose of study drug will be documented as a treatment-emergent AE. This rule is consistent with the treatment-emergent signs and symptoms convention for counting AEs.

All AEs (i.e. pretreatment AE and AEs that occurred after initiation of study therapy) will be reported. All AEs will be split between pre-treatment, treatment and post-treatment phases. All AEs need to be collected and reported after signature of the informed consent. AE observation will end 28 days after end of study or 100 days after the last study drug administration, unless the investigator suspects a delayed adverse reaction to the study drug.

Specific forms will be used for expedited reporting of SAEs.

### 10.2.3 Events not to be treated as AEs

- Preplanned interventions or occurrence of endpoints specified in the study protocol are not considered AE’s, if not defined otherwise (e.g. as a result of overdose)

- Medical or surgical procedures, e.g. surgery, endoscopy, tooth extraction, transfusion that are not associated with the indication under study. However, the event leading to the procedure is an AE. If this event is serious, the procedure must be described in the SAE narrative.
- Pre-existing disease or medical condition prior to the screening visit that does not worsen
- Situations in which an adverse event change did not occur, e.g. hospitalizations for cosmetic elective surgery or for social and/or convenience reasons. In addition, the following reasons for hospitalizations are not considered AEs, and therefore not SAEs:
  - Hospitalizations for cosmetic elective surgery, social and/or convenience reasons. Treatment, which was elective or pre-planned, for a pre-existing condition that is unrelated to the indication under study and did not worsen
  - Standard monitoring of a pre-existing disease or medical condition that did not worsen, e.g., hospitalization for coronary angiography in a subject with stable angina pectoris
  - Elective treatment of a pre-existing disease or medical condition that did not worsen, e.g. elective hip replacement for arthritis
  - Routine treatment or monitoring of the studied indication, not associated with any deterioration in condition
  - Admission to a hospital or other institution for general care, not associated with any deterioration in condition; or treatment on an emergency, outpatient basis for an event not fulfilling any of the definitions of serious and not resulting in hospital admission

### 10.2.4 Events not to be treated as SAEs

Due to the seriousness of the disease in this study, certain conditions defined as SAEs will be excluded from expedited reporting on a SAE report form (exemptions allowed according to §12, Abs. 4, GCP-V):

- Elective hospitalization and surgery for treatment of the underlying disease including transfusions
- The scheduled surgical resection of the lung cancer or planned diagnostic procedures related to this surgery
- Elective hospitalization to simplify treatment or study procedures
- Events that are only and unequivocally caused by progression of the underlying disease

Primary disease progression and related SAEs e.g. hospitalization for surgery/diagnostic procedures, except for life threatening status or death caused by the underlying malignant disease will not be considered and reported as SAEs if they are unrelated to study medication according to the investigator. The assessment of relationship to study medication must also be documented.

Further information for safety reporting can be found in the study specific Safety Manual.

## 10.3 Contact information for safety reporting

The investigator will inform the sponsor of the occurrence or receipt of knowledge of the occurrence of an SAE or pregnancy without delay, at the latest within 24 hours of being made aware of the SAE/pregnancy. Each SAE and pregnancy must be followed up until resolution or stabilization by submission of updated reports (FU-report) to the designated recipient.

The above information is to be entered by qualified site staff into the respective safety reporting form of the eCRF in English language. An email report will be generated immediately and automatically after saving and will be sent to the Sponsor´s Drug Safety, to the Coordinating Investigator Prof. Dr. Martin Schuler and to BMS Global Pharmacovigilance (Worldwide.Safety@bms.com).

In the event that electronic reporting is not possible, paper safety reporting forms (see section available in the investigator site's file handed out at the beginning of the study) have to be used by the site staff for notification via conventional fax or email (by reference to the current contact information presented in the header of the paper forms).

## 10.4 Investigator reporting responsibilities.

The investigator has to keep copies of all adverse event information, including correspondence with the sponsor and the Ethics committee (EC)/IRB, on file.

If an adverse event is serious (see definition above), additional reporting to the sponsor is required.

The investigator must inform the sponsor of any SAE immediately but not later than 24 hours of being aware of the event, in case of a suspected pregnancy or fetal exposure immediately.

Information not available at the time of initial reporting should be provided in a follow-up.

## 10.5 Notification of SAEs to the EC/IRB

Notification of the IECs / IRBs about all relevant events (e.g. SAEs, suspected, unexpected, serious adverse reactions [SUSARs]) will be performed by the sponsor according to all applicable regulations.

## 10.6 Notification of the competent authority

Notification of the competent authority on all relevant events (e.g. SAEs, suspected, unexpected, serious adverse reactions [SUSARs]) will be performed by the sponsor according to all

applicable regulations.

## 10.7 Notification of the manufacturer

All Serious Adverse Events (SAEs) as well as all suspected pregnancies/lactation that occur following the subject's written consent to participate in the study through 100 days of discontinuation of dosing must be reported by the sponsor to BMS Worldwide Safety, whether related or not related to study drug. If applicable, SAEs must be collected that relate to any later protocol-specified procedure (e.g., a follow-up skin biopsy).

- SAEs, whether related or not related to study drug, and pregnancies must be reported to BMS within 24 hours / 1 Business Day of becoming aware of the event.

**SAE Email Address:** Worldwide.Safety@BMS.com

**SAE Facsimile Number:** +1 609 818-3804

If only limited information is initially available, follow-up reports are required. (Note: Follow-up SAE reports should include the same investigator term(s) initially reported.)

If an ongoing SAE changes in its intensity or relationship to study drug or if new information becomes available, a follow-up SAE report should be sent within 24 hours \ 1 Business Day to BMS using the same procedure used for transmitting the initial SAE report.

## 10.8 Sponsors reporting responsibilities

The processing and reporting of all relevant events (e.g. SAEs, SUSARs) to the authorities will be done by the sponsor according to all applicable regulations.

For all SAEs the sponsor has to carry out a separate assessment for expectedness, seriousness and causal relationship to treatment with the study medication.

Each SUSAR that becomes known in this clinical study will be reported by the sponsor to the competent authority and the IECs / IRBs and to the manufacturer of the IMP.

The sponsor will inform all investigational sites about reported relevant events (e.g. SUSARs) according to all applicable regulations.

## 10.9 Development Safety Update Report (DSUR)

Once a year or on demand, the sponsor will supply a report on the safety of study subjects in accordance with ENTR/CT 3 with all relevant information during the reference period to the competent supreme federal authority, the responsible ethics committee and to BMS.

The reference period for the DSUR begins with the date that the study is approved by the

competent supreme federal authority. This date is the reference date for the start of the year of the annual safety report. The sponsor will supply the report within 60 days of one year after the reference date (data-lock point).

Further details will be given in the study-specific Safety Manual.

## 10.10 Expected adverse events

For this study, the applicable reference documents are the most current versions of the IBs for nivolumab and relatlimab. If relevant new safety information is identified, the information will be integrated into an update of the reference documents and distributed to all participating sites.

The expectedness of AEs will be determined by the sponsor according to the applicable reference documents and according to all local regulations.

### 10.10.1 Expected adverse events related to treatment with OPDIVO® (nivolumab)

Please refer to the current IB of nivolumab for a summary of the safety profile and an exhaustive list of ADRs reported from controlled clinical studies and post-marketing experience. Updated IBs are regularly provided.

### 10.10.2 Expected adverse events related to treatment with relatlimab

Please refer to the current IB of relatlimab for a summary of the safety profile. Updated IBs are regularly provided.

## 10.11 Pregnancies

Investigators shall counsel WOCBP, and male participants who are sexually active with WOCBP, on the importance of pregnancy prevention and the implications of an unexpected pregnancy and when applicable, the potential of fetal toxicity occurring due to transmission of study drug, present in seminal fluid, to a developing fetus, even if the participant has undergone a successful vasectomy or if the partner is pregnant. Investigators shall advise on the use of highly effective methods of contraception (Appendix IV), which have a failure rate of < 1% when used consistently and correctly.

Fertile women have to agree not to become pregnant while in this study or within 6 months after receiving the last study treatment.

Any abnormal pregnancy outcomes (e.g. spontaneous abortion, fetal death, stillbirth, congenital anomalies, and ectopic pregnancy) are considered as serious adverse events and must be reported using the **Serious Adverse Event Form**.

The sponsor/investigator or study-site personnel shall report to BMS Global Pharmacovigilance (Worldwide.Safety@bms.com) within 24 hours any information related to pregnancies or suspected pregnancies (including positive pregnancy tests regardless of age or disease state)

occurring in patients or partners of patients while the patients are still treated with the study medication and during the last 5months after last application. When reporting information under this provision, the sponsor/investigator shall use the **Pregnancy Reporting Form** provided by the sponsor.

Female patients

If a patient gets pregnant while the patient is still treated with the study medication the investigator shall immediately:

- Discontinue the treatment with study medication
- Refer the patient to an obstetrician/gynecologist experienced in reproductive toxicity for further evaluation and counseling.

The investigator shall follow pregnant patients until the end of the pregnancy and must notify the sponsor and BMS Global Pharmacovigilance (Worldwide.Safety@bms.com) about the outcome of the pregnancy (including false-positive pregnancy tests) within 24 hours of having knowledge of the event.

The sponsor/investigator shall report within 24 hours:

- Any outcome of a pregnancy which qualifies as a serious adverse event (as defined above, i.e., spontaneous or therapeutic abortion, fetal and neonatal death or congenital anomaly)
- Any death of an infant which occurs in connection with in utero exposure to the study medication within 28 days of birth
- The investigator shall also document any congenital anomaly detected in an aborted fetus.

Male patients

Men should be advised not to father a child while receiving treatment and must use effective contraception during and for a total of 225 days (approximately 33 weeks)after treatment. Before starting treatment, male patients should be advised to seek counseling on sperm storage.

Because the effect of the study drug on sperm is unknown, pregnancies in partners of male subjects included in the study will be reported by the study-site personnel within 24 hours of their knowledge of the event using the appropriate **Pregnancy Reporting Form**.

The investigator shall use his/her best efforts in order to ensure that male patients:

- Inform him/her if his partner gets pregnant while the patient is still treated with the study medication
- Provide him/her the contact details of the healthcare provider who follows the pregnancy in question

If a pregnancy related event is reported in a female partner of a male subject, the investigator should ask if the female partner is willing to share information with BMS Global Pharmacovigilance (Worldwide.Safety@bms.com) and allow the pregnancy related event to be

followed up to completion. The **Pregnant Partner Form** will be provided by the sponsor.

If the partner of a male patient gets pregnant while the patient is still treated with the study medication the investigator shall:

- Advise that the partner consults her general practitioner or gynecologist as soon as possible
- Immediately provide the information to BMS Global Pharmacovigilance (Worldwide.Safety@bms.com) (contact details: see Section 10.3).

The investigator shall be responsible for any decision regarding the continued participation in the study of patients who after an initial positive pregnancy diagnosis appear to be no longer pregnant.

### 10.12 Overdose

All cases of overdose, misuse, application errors etc. should be documented, even without the occurrence of an AE or SAE. All occurrences of overdose of the IMP must be reported as an SAE.

### 10.13 Data monitoring committee

An independent Data safety monitoring board (DSMB) will be formed to oversee the safety of the trial subjects in the clinical trial by periodically assessing the safety of the trial therapy. The DSMB will consist of two physicians who are not involved in the trial and who are external to the sponsor. The DSMB will act in an advisory capacity to the sponsor and will monitor subject safety and evaluate the available efficacy data for the study. The sponsor has primary responsibility for design and conduct of the study. The study DSMB charter will elaborate the guidelines for the DSMB.

# 11 Statistical methods and sample size calculation

### 11.1 General statistical considerations

All parameters, except the primary endpoint, will be evaluated in an explorative or descriptive manner, providing means, medians, ranges, standard deviations and/or confidence intervals. All p values will be two-sided if not stated otherwise.

The amount and subsets of tumor infiltrating lymphocytes and peripheral immune cells before and after therapy will be analyzed using t-test, Mann-Whitney-U test or ANOVA. Overall response rate

(ORR), toxicity and other event rates in each arm will be compared using Fisher's exact test and χ2 test; odds ratios with confidence intervals and p-value will be provided.

DFS and OS will be analysed using Kaplan-Meier methods and compared by log rank test. Univariate and multivariate analyses will eventually be performed by suitable regression models (logistic regression, proportional hazard regression model).

Missing values will not be imputed. Outliers will be identified prior to the analyses of the efficacy endpoints and have to be checked by the study physician. According to the decision of the data management, the statistician and the study physician, outliers will be kept in the database or set to "missing".

## 11.2 Analysis sets

Intent-to-Treat Population (ITT)

The ITT population will consist of all patients who provided informed consent and received at least one dose of study drug.

Per-Protocol Population (PP)

The PP Population will consist of all patients from the ITT Population who did not show major deviations from the protocol procedures. Patients will be excluded from the per-protocol analysis set, if at least one of the following criteria is met: administration of less than two doses of study medication, no surgery.

Full analysis set (FAS)

Safety summaries will be based on the FAS, which will consist of all patients who provided informed consent and received at least one dose of study drug.

## 11.3 Sample size

The primary objective of this study is to determine the feasibility of two cycles of preoperative immunotherapy in patients eligible for curative anatomic resection of NSCLC. Per study arm 30 evaluable patients will be enrolled. Sequential boundaries will be used to monitor the non-feasibility rate. The accrual for each study arm will be halted if excessive numbers of delays of curative surgery beyond 6 weeks from initiation of study treatment are seen, that is, if the number of delayed surgeries is equal to or exceeds $b_n$ out of $n$ patients with sufficient follow-up (see table). This is a Pocock-type stopping boundary that yields the probability of crossing the boundary at most 0.05 (probability of early stopping) when the rate of delayed surgeries is equal to the acceptable rate 0.05 (event probability $\theta$).

| $n$ | 1 | 2 | 3 | 4 | 5 | 6 | 7 | 8 | 9 | 10 | 11 | 12 | 13 | 14 | 15 | 16 | 17 | 18 | 19 | 20 |
|---|---|---|---|---|---|---|---|---|---|---|---|---|---|---|---|---|---|---|---|---|
| $b_n$ | - | 2 | 2 | 2 | 3 | 3 | 3 | 3 | 3 | 3 | 3 | 3 | 4 | 4 | 4 | 4 | 4 | 4 | 4 | 4 |

| *n* | 21 | 22 | 23 | 24 | 25 | 26 | 27 | 28 | 29 | 30 |
|---|---|---|---|---|---|---|---|---|---|---|
| $b_n$ | 4 | 4 | 5 | 5 | 5 | 5 | 5 | 5 | 5 | 5 |

This boundary is equivalent to testing the null hypothesis after each patient, that the event rate is equal to 0.05, using a one-sided level 0.022387 test.

## 11.4 Randomization / Stratification

Patients will be allocated to one of the two treatment arms for up to 30 patients in each arm. Arms A and B will be opened in parallel.

No stratification is planned.

## 11.5 Variables and planned statistical analyses

### 11.5.1 Variables

Primary and secondary variables are specified in Section 5.2.

### 11.5.2 Statistical methods

A detailed Statistical Analysis Plan (SAP) will be compiled and reviewed prior to the end of the data management process.

Descriptive analyses

Summary statistics (arithmetic mean, standard deviation, median, minimum and maximum for quantitative variables) will be presented by treatment group. Frequency tables for categorical data will be provided. Medical history findings will be summarized using MedDRA terms.

Safety examinations

Individual listings of AEs will be provided. The occurrence of treatment-emergent AEs and drug-related AEs, respectively, will be summarized by treatment using MedDRA terms. All AEs starting or worsening after first study drug administration up to 150 days after last study drug administration will be considered as treatment-emergent.

Quantitative data (hematology, clinical chemistry, vital signs, clotting status) will be described descriptively. These summary statistics will be presented by treatment for the original data as well as for the difference to baseline. Frequency tables will be provided for qualitative data.

Primary and secondary endpoints

All parameters except the feasibility rate will be evaluated in an explorative or descriptive manner, providing means, medians, ranges, standard deviations and/or confidence intervals. All p values will be two-sided if not stated otherwise.

The amount and subsets of tumor infiltrating lymphocytes and peripheral immune cells before and after therapy will be analyzed using t-test, Mann-Whitney-U test or ANOVA. ORR, toxicity and other event rates in each arm will be compared using Fishers exact test and $\chi 2$ test; odds ratios with confidence intervals and p-value will be provided.

DFS and OS will be analyzed using Kaplan-Meier methods and compared by log rank test. Univariate- and multivariate analyses will be performed by suitable regression models (logistic regression, proportional hazard regression model).

### 11.6 Subgroup analyses

Subgroup analyses are not planned in the study. Each treatment arm will be analyzed separately for the study endpoints.

### 11.7 Interim analysis

Since the primary endpoint is continuously monitored, no interim analysis is planned. In case of crossing the predefined boundary for non-feasibility the respective arm will be closed for further enrolment.

### 11.8 Analysis and reporting

Data will be analyzed and the report will be prepared after the last subject has finished the follow-up period of 12 months. The integrated clinical/biometrical report will be performed within 6 months after completion and correction of all case report forms (database lock).

# 12 Documentation

All data relevant to this study are to be documented by the responsible investigator in a eCRF immediately after measurement has been taken. Entering data may be delegated to members of the study team but the investigator will have to check and sign the eCRF entries. Paper forms for reporting of SAEs have to be filled in, signed and dated by the investigator. All paper-based documentation should be completed legibly, in black or blue ink. If it is necessary to make corrections, a single line should be drawn through the original entry, the new entry written in, and the form initialed and dated by the individual making the correction. Original records, source data, printouts from medical devices will be documented in a separate patient file.

Any copies of source data printouts related directly to the study that will be collected by the monitor will have only the subject number entered as an identifier (any personal subject information will be made illegible (e.g. blacked out) by the site staff in order to maintain the subject´s confidentiality).

## 12.1 Study records requirements

The investigator must ensure that the records and documents pertaining to the conduct of the study and the distribution of the study drug, that is copies of eCRFs and source documents (original documents, data, and records [e.g., hospital records; clinical and office charts; laboratory notes; memoranda; subject’s diaries or evaluation checklists; pharmacy dispensing records; recorded data from automated instruments; copies or transcriptions certified after verification as being accurate copies; microfiches; photographic negatives, microfilm, or magnetic media; x-rays; subject files; and records kept at the pharmacy, at the laboratories, and at medico-technical departments involved in the clinical study; documents regarding subject treatment and study drug accountability; original signed informed consents, etc.]) be retained by the investigator for as long as needed to comply with national and international regulations (generally 2 years after discontinuing clinical development or after the last marketing approval). According to local regulations it is the Sponsor’s responsibility to store essential documents. The investigator agrees to adhere to the document/records retention procedures by signing the protocol.

## 12.2 Data management

Alcedis GmbH (Gießen, Germany) will provide the IT infrastructure and data management staff.

The study database will be developed and validated prior to data entry based on standard operating procedures. The data management system is based on commercial trial software and stores the data in a database, storing all changes in an audit trail. The trial software has a user and role concept that can be adjusted on a study- and site- specific basis. The database is integrated into a general IT infrastructure and safety concept with a firewall and backup system. A regularly backup of all study data will be performed. After completion and cleaning of data, the database is locked and the data exported for statistical analysis.

Study sites will enter data online via internet. Plausibility checks are run during data entry, thereby detecting discrepancies immediately. The Data Management will conduct further checks for completeness and plausibility and will clarify any questions with the study sites electronically via trial software. Discrepancies and implausible values will be clarified in writing between the Data Manager and the study site. These electronic queries have to be answered by the study site without unreasonable delay. Further details will be specified in the Data Management Manual.

Entries made in the eCRF must be either verifiable against source documents, or have been directly entered into the eCRF, in which case the entry in the eCRF will be considered as the source data.

## 12.3 Archiving

All eCRFs, informed consent forms and other important study materials will be archived for at least 25 years.

Essential documents shall be archived in such a way that ensures that they are readily available upon authorities' request.

Patient (hospital) files will be archived according to local regulations and in accordance with the maximum period of time permitted by the institution. Where the archiving procedures do not meet the minimum timelines required by the sponsor, alternative arrangements must be made to ensure the availability of the source documents for the required period.

The Investigator/institution notifies the sponsor if the archival arrangements change (e.g. relocation or transfer of ownership). The ISF is not to be destroyed without the sponsor's approval. The Investigator's contract will contain all regulations relevant for the study center.

## 12.4 Monitoring

The study site will be monitored to ensure the quality of the data collected. The objectives of the monitoring procedures are to ensure that the study subject's safety and rights as a study participant are respected, that accurate, valid and complete data are collected, and that the study is conducted in accordance with the study protocol, the principles of GCP and local legislation.

All investigators agree that the monitor will regularly visit each study site and assure that the monitor will receive appropriate support in his/her activities at the study site, as agreed in separate contracts with each site. The declaration of informed consent includes a statement to the effect that the monitor has the right – while observing the provisions of data protection legislation – to compare the eCRFs with the study subject's medical records (physician´s notes, laboratory printouts etc.). The Investigator will ensure access for the monitor to all necessary documentation for study-related monitoring. The purposes of the monitor visits are as follows:

- Check the informed consent
- Check inclusion and exclusion criteria
- Monitor study subject safety (occurrence and documentation/reporting of AEs and SAEs)
- Check the completeness and accuracy of entries on the eCRFs
- Validate the entries on the eCRFs against those in the source documents (source data verification)
- Perform drug accountability checks
- Evaluate the progress of the study
- Evaluate compliance with the study protocol
- Assess whether the study is being performed according to GCP at the study site
- Discuss with the Investigator aspects of study conduct and any deficiencies found

Monitoring at each study site will be done for 100% for the ISF, the patient's informed consent, all inclusion and exclusion criteria and the drug accountability.

In case onsite visits are not possible for a longer time period (e.g. due to a pandemic situation) remote monitoring will be performed. The monitor will schedule remote visits with the study site to discuss study data. Details will be specified in the monitoring plan.

A monitoring visit report will be prepared for each visit describing the progress of the clinical study and any problems (e.g. refusal to give access to documentation).

Further details will be given in the Monitoring Manual.

### 12.5 Audits/Inspections

As part of quality assurance, the sponsor has the right to audit the study site and any other institutions involved in the study. The aim of an audit is to verify the validity, accuracy and completeness of data, to establish the credibility of the clinical study, and to check whether the study subject’s rights and study subject safety are being maintained. The sponsor may assign these activities to persons otherwise not involved in the study (auditors). These persons are allowed access to all study documentation (especially the study protocol, case report forms, study subjects’ medical records, drug accountability documentation, and study-related correspondence).

In addition, inspections by regulatory health authority representatives and EC/IRB are possible. The Investigator should notify the sponsor immediately of any such inspection.

The Investigator/institution agrees to allow the auditor or inspector direct access to all relevant documents and allocate his/her time and the time of his/her staff to the auditor/inspector to discuss findings and any issues. Audits and inspections may occur at any time during or after completion of the study.

All persons conducting audits will keep all study subject data and other study data confidential.

# 13 Ethical and regulatory aspects

### 13.1 Independent ethics committee

The protocol, informed consent form (ICF) and any other supporting study documents will be reviewed by an independent ethics committee (IEC). Each study site may not begin the study until the responsible ethics committee for that site has given its written approval, signed by the authority chairperson or authorized personnel, and a copy of the approval letter and the approved ICF for that site have been provided to the Investigator.

## 13.2 Notification of the authorities, approval and registration

Before the start of the clinical study, all necessary documentation will be submitted to the competent supreme federal authority for approval. The state authorities in each federal state in which the study will be conducted will also be notified.

Prior to start of the study, it will be registered under *www.clinicaltrials.gov*.

## 13.3 Ethical basis for the clinical study

This protocol is designed to ensure that the sponsor and investigator(s) abide by Good Clinical Practice (GCP) guidelines, the Declaration of Helsinki and local regulations governing the conduct of clinical studies.

Documented approval from appropriate IECs will be obtained for all participating centers before start of the study. When necessary, an extension, amendment or renewal of the EC approval must be obtained. Strict adherence to all specifications laid down in this protocol is required for all aspects of study conduct, i.e. the investigator is not allowed to modify the procedures described in this protocol.

Modifications to the study protocol should be implemented only after agreement between sponsor, CPI and investigator(s). However, the investigator or the sponsor may implement a change of the protocol to eliminate an immediate hazard to the study subjects without prior IEC/sponsor approval/favorable opinion. As soon as possible, the implemented change and the reasons for it should be submitted to the IEC/head of medical institution/sponsor. Any deviations from the protocol must be explained and documented by the investigator.

All investigators and other staff involved in the study will be informed that the competent federal authorities and authorized representatives of the sponsor have the right to review study documentation and the study subjects' medical records at any time.

Details on discontinuation of the entire study or parts thereof can be found in Section 14.

## 13.4 Obtaining informed consent from study subjects

All relevant information on the study will be summarized in a subject information sheet and informed consent form (ICF) provided by the sponsor. A sample subject information and ICF is provided as a document separate to this protocol.

Based on the subject information sheet, the investigator or designee will explain all relevant aspects of the study to each subject prior to her/his entry into the study (i.e. before any examinations and procedures associated with the selection for the study are performed or any study-specific data is recorded on study-specific forms.In case laboratory or imaging procedures were performed for clinical reasons prior to signing informed consent, these can be used for screening purposes with consent of the patient. However, all screening laboratory and imaging results must have been obtained within 28 days of randomization.). The investigator will also

mention that written approval of the IEC has been obtained.

Each subject will have ample time and opportunity to ask questions and will be informed about the right to withdraw from the study at any time without any disadvantage and without having to provide reasons for this decision.

Only if the subject voluntarily agrees to sign the informed consent form and has done so, may she/he enter the study. Additionally, the investigator and other information provider (if any) will personally sign and date the form. The ICF will be signed in duplicate, one copy remains with the subject and the second original is to remain in the ISF or, if locally required, in the patient's note/file of the medical institution.

In the event that informed consent is obtained on the date that baseline study procedures are performed, the study record or subject´s clinical record must clearly show that informed consent was obtained prior to these procedures.

The ICF and any other written information provided to subjects will be revised whenever important new information becomes available that may be relevant to the subject's consent, or there is an amendment to the protocol that necessitates a change to the content of the subject information and / or the written ICF. The investigator will inform the subject of changes in a timely manner and will ask the subject to confirm her/his participation in the study by signing the revised ICF. Any revised written ICF and written information must receive the IECs approval / favorable opinion in advance of use.

Part of the monitoring activities are to check that the most recent ICF was used before the study subject was enrolled and that it was dated and signed by the study subject himself or herself.

## 13.5 Protocol deviations

When an emergency occurs that requires a deviation from the protocol for a subject, a deviation will be made only for that subject. A decision will be made as soon as possible to determine whether or not the subject (for whom the deviation from protocol was effected) is to continue in the study. The subject's medical records will completely describe the deviation from the protocol and state the reasons for such deviation.

## 13.6 Compensation for health damage of subjects

Patients will be insured by the sponsor against injury caused by their participation in the study according to legal requirements. The patients will be informed about the insurance and the requirements on their part.

## 13.7 Confidentiality

All records identifying the subject will be kept confidential and, to the extent permitted by the applicable laws and / or regulations, will not be made publicly available.

Subject names will not be supplied to the sponsor. Only the subject number will be recorded in the eCRF, and if the subject name appears on any other document, it must be obliterated before a copy of the document is supplied to the sponsor. As part of the informed consent process, the subjects will be informed in writing that representatives of the sponsor, IEC, or regulatory authorities may inspect their medical records to verify the information collected, and that all personal information made available for inspection will be handled in strictest confidence and in accordance with local data protection laws.

If the results of the study are published, the subject's identity will remain confidential. The investigator will maintain a list to enable subjects to be identified in the ISF.

# 14 Closure of study sites/Premature termination of the clinical study

The sponsor might close this study or parts thereof at any time if

- risk-benefit ratio becomes unacceptable based on safety findings or any interim analysis from this study or upcoming information from other clinical or animal studies
- the study conduct does not suggest a proper completion of the study within a reasonable time frame

The investigator has the right to close his/her center at any time.

All closures should occur only after consultation between sponsor, CPI and study center(s). All affected institutions (e.g. IEC(s), competent authorities study center) must be informed as applicable according to local law. All study materials (except documentation that has to remain stored at site) must be returned to the sponsor. The investigator will retain all other documents until notification given by the sponsor for destruction.

# 15 Publication

It is planned to publish the clinical study results, in mutual agreement with the coordinating principal investigator, in a scientific journal and at international congresses. Publication of the results of the clinical study as a whole is intended. Additional publications are foreseen reporting specific aspects of the translational research program. It is agreed that the full publication of the clinical study has to be accepted before secondary publications (subgroups, translational research) are submitted. Any publication will take account of the 'Uniform requirements for manuscripts submitted to biomedical journals (International Committee of Medical Journal Editors (ICMJE). The study publication will be led by the coordinating investigator; authorship and coauthorship will based on merit and significant patient enrolment according to international publication regulations.

The study will also be registered in a public register (www.clinicaltrials.gov) in accordance with

the recommendations of the ICMJE.

Any published data will observe data protection legislation covering the study subject and investigators. Success rates or individual findings at individual study sites are known only to the sponsor.

Publications or lectures on the findings of the present clinical study either as a whole or at individual investigation sites must be approved by the sponsor in advance, and the sponsor reserves the right to review and comment on such documentation before publication.

By signing the contract to participate in this study, the investigator declares that he or she agrees to submission of the results of this study to national and international authorities for approval and surveillance purposes, and to the Federal Physicians Association, the Association of Statutory Health Fund Physicians and to statutory health fund organizations, if required. At the same time, the investigator agrees that his or her name, address, qualifications and details of his or her involvement in the clinical study may be made known to these bodies.

# 16 Amendments to the study protocol

To ensure that comparable conditions are achieved as far as possible at individual study sites and in the interests of a consistent and valid data analysis, changes to the provisions of this study protocol are not planned. In exceptional cases, however, changes may be made to the study protocol. Such changes can only be made if agreed by the sponsor, sponsor's representative, the CPI and statistician, and all authors of this study protocol. Any changes to the study procedures must be made in writing and must be documented with reasons and signed by all authors of the original study protocol.

Amendments made in accordance with § 10 Secs. 1 and 4 GCP Regulations that require approval are submitted to the ethics committee and the supreme federal authority and will not be implemented until approved. Exceptions to this are amendments made to avoid immediate dangers.

# 17 References

**1.** Forde PM, Chaft JE, Smith KN, et al. Neoadjuvant PD-1 Blockade in Resectable Lung Cancer. N Engl J Med 2018;378:1976–86.

**2.** Goldstraw P, Chansky K, Crowley J, et al. The IASLC Lung Cancer Staging Project: Proposals for Revision of the TNM Stage Groupings in the Forthcoming (Eighth) Edition of the TNM Classification for Lung Cancer. J Thorac Oncol 2016;11:39–51.

**3.** Morgensztern D, Du L, Waqar SN, et al. Adjuvant Chemotherapy for Patients with T2N0M0 NSCLC. J Thorac Oncol 2016;11:1729–35.

**4.** Arriagada R, Bergman B, Dunant A, Le Chevalier T, Pignon J-P, Vansteenkiste J. Cisplatin-based adjuvant chemotherapy in patients with completely resected non-small-cell lung cancer. N Engl J Med 2004;350:351–60.

**5.** Winton T, Livingston R, Johnson D, et al. Vinorelbine plus cisplatin vs. observation in resected non-small-cell lung cancer. N Engl J Med 2005;352:2589–97.

**6.** Douillard J-Y, Rosell R, Lena M de, et al. Adjuvant vinorelbine plus cisplatin versus observation in patients with completely resected stage IB-IIIA non-small-cell lung cancer (Adjuvant Navelbine International Trialist Association ANITA): a randomised controlled trial. Lancet Oncol 2006;7:719–27.

**7.** Brahmer J, Reckamp KL, Baas P, et al. Nivolumab versus Docetaxel in Advanced Squamous-Cell Non-Small-Cell Lung Cancer. N Engl J Med 2015;373:123–35.

**8.** Borghaei H, Paz-Ares L, Horn L, et al. Nivolumab versus Docetaxel in Advanced Nonsquamous Non-Small-Cell Lung Cancer. N Engl J Med 2015;373:1627–39.

**9.** Herbst RS, Baas P, Kim D-W, et al. Pembrolizumab versus docetaxel for previously treated, PD-L1-positive, advanced non-small-cell lung cancer (KEYNOTE-010): a randomised controlled trial. Lancet 2016;387:1540–50.

**10.** Reck M, Rodríguez-Abreu D, Robinson AG, et al. Pembrolizumab versus Chemotherapy for PD-L1-Positive Non-Small-Cell Lung Cancer. N Engl J Med 2016;375:1823–33.

**11.** Rittmeyer A, Barlesi F, Waterkamp D, et al. Atezolizumab versus docetaxel in patients with previously treated non-small-cell lung cancer (OAK): a phase 3, open-label, multicentre randomised controlled trial. The Lancet 2017;389:255–65.

**12.** Antonia SJ, Villegas A, Daniel D, et al. Durvalumab after Chemoradiotherapy in Stage III Non-Small-Cell Lung Cancer. N Engl J Med 2017;377:1919–29.

**13.** Hellmann MD, Ciuleanu T-E, Pluzanski A, et al. Nivolumab plus Ipilimumab in Lung Cancer with a High Tumor Mutational Burden. N Engl J Med 2018;378:2093–104.

**14.** Chen DS, Mellman I. Elements of cancer immunity and the cancer-immune set point. Nature 2017;541:321–30.

**15.** Eggermont AMM, Chiarion-Sileni V, Grob J-J, et al. Prolonged Survival in Stage III Melanoma with Ipilimumab Adjuvant Therapy. N Engl J Med 2016;375:1845–55.

**16.** Weber J, Mandala M, Del Vecchio M, et al. Adjuvant Nivolumab versus Ipilimumab in Resected Stage III or IV Melanoma. N Engl J Med 2017;377:1824–35.

**17.** Antonia SJ, Villegas A, Daniel D, et al. Overall Survival with Durvalumab after Chemoradiotherapy in Stage III NSCLC. N Engl J Med 2018;379:2342–50.

**18.** Preoperative chemotherapy for non-small-cell lung cancer: a systematic review and meta-analysis of individual participant data. The Lancet 2014;383:1561–71.

**19.** Scagliotti GV, Pastorino U, Vansteenkiste JF, et al. Randomized phase III study of surgery alone or surgery plus preoperative cisplatin and gemcitabine in stages IB to IIIA non-small-cell lung cancer. J Clin Oncol 2012;30:172–8.

**20.** Felip E, Rosell R, Maestre JA, et al. Preoperative chemotherapy plus surgery versus surgery plus adjuvant chemotherapy versus surgery alone in early-stage non-small-cell lung cancer. J Clin Oncol 2010;28:3138–45.

**21.** Felip E, Rosell R, Alberola V, et al. Preoperative high-dose cisplatin versus moderate-dose cisplatin combined with ifosfamide and mitomycin in stage IIIA (N2) non small-cell lung cancer: results of a randomized multicenter trial. Clin Lung Cancer 2000;1:287–93.

**22.** Rusch V, Chaft J, Johnson B, et al. MA04.09 Neoadjuvant Atezolizumab in Resectable Non-Small Cell Lung Cancer (NSCLC): Updated Results from a Multicenter Study (LCMC3). Journal of Thoracic Oncology 2018;13:S369.

**23.** Cascone T, William WN, Weissferdt A, et al. LBA49Neoadjuvant nivolumab (N) or nivolumab plus ipilimumab (NI) for resectable non-small cell lung cancer (NSCLC). Annals of Oncology 2018;29.

**24.** Chalabi M, Fanchi LF, van den Berg JG, et al. LBA37_PRNeoadjuvant ipilimumab plus nivolumab in early stage colon cancer. Annals of Oncology 2018;29.

**25.** Necchi A, Anichini A, Raggi D, et al. Pembrolizumab as Neoadjuvant Therapy Before Radical Cystectomy in Patients With Muscle-Invasive Urothelial Bladder Carcinoma (PURE-01): An Open-Label, Single-Arm, Phase II Study. J Clin Oncol 2018:JCO1801148.

**26.** Carbone DP, Reck M, Paz-Ares L, et al. First-Line Nivolumab in Stage IV or Recurrent Non-Small-Cell Lung Cancer. N Engl J Med 2017;376:2415–26.

**27.** Hellmann MD, Paz-Ares L, Bernabe Caro R, et al. Nivolumab plus Ipilimumab in Advanced Non-Small-Cell Lung Cancer. N Engl J Med 2019 [Epub ahead of print].

**28.** Naidoo J, Wang X, Woo KM, et al. Pneumonitis in Patients Treated With Anti-Programmed Death-1/Programmed Death Ligand 1 Therapy. J Clin Oncol 2017;35:709–17.

**29.** Pardoll D. Does the immune system see tumors as foreign or self? Annu Rev Immunol 2003;21:807–39.

**30.** Zitvogel L, Tesniere A, Kroemer G. Cancer despite immunosurveillance: immunoselection and immunosubversion. Nat Rev Immunol 2006;6:715–27.

**31.** Dunn GP, Bruce AT, Ikeda H, Old LJ, Schreiber RD. Cancer immunoediting: from immunosurveillance to tumor escape. Nat Immunol 2002;3:991–8.

**32.** Greenwald RJ, Freeman GJ, Sharpe AH. The B7 family revisited. Annu Rev Immunol 2005;23:515–48.

**33.** Freeman GJ, Long AJ, Iwai Y, et al. Engagement of the PD-1 immunoinhibitory receptor by a novel B7 family member leads to negative regulation of lymphocyte activation. J Exp Med 2000;192:1027–34.

**34.** Sharpe AH, Wherry EJ, Ahmed R, Freeman GJ. The function of programmed cell death 1 and its ligands in regulating autoimmunity and infection. Nat Immunol 2007;8:239–45.

**35.** Triebel F, Jitsukawa S, Baixeras E, et al. LAG-3, a novel lymphocyte activation gene closely related to CD4. J Exp Med 1990;171:1393–405.

**36.** Huard B, Prigent P, Tournier M, Bruniquel D, Triebel F. CD4/major histocompatibility complex class II interaction analyzed with CD4- and lymphocyte activation gene-3 (LAG-3)-Ig fusion proteins. Eur J Immunol 1995;25:2718–21.

**37.** Workman CJ, Rice DS, Dugger KJ, Kurschner C, Vignali DAA. Phenotypic analysis of the murine CD4-related glycoprotein, CD223 (LAG-3). Eur J Immunol 2002;32:2255–63.

**38.** Hannier S, Tournier M, Bismuth G, Triebel F. CD3/TCR complex-associated lymphocyte activation gene-3 molecules inhibit CD3/TCR signaling. J Immunol 1998;161:4058–65.

**39.** Okazaki T, Okazaki I-m, Wang J, et al. PD-1 and LAG-3 inhibitory co-receptors act synergistically to prevent autoimmunity in mice. J Exp Med 2011;208:395–407.

**40.** Workman CJ, Vignali DAA. Negative regulation of T cell homeostasis by lymphocyte activation gene-3 (CD223). J Immunol 2005;174:688–95.

**41.** Huang C-T, Workman CJ, Flies D, et al. Role of LAG-3 in regulatory T cells. Immunity 2004;21:503–13.

**42.** Blackburn SD, Shin H, Haining WN, et al. Coregulation of CD8+ T cell exhaustion by multiple inhibitory receptors during chronic viral infection. Nat Immunol 2009;10:29–37.

**43.** Yang C-FJ, Wang H, Kumar A, et al. Impact of Timing of Lobectomy on Survival for Clinical Stage IA Lung Squamous Cell Carcinoma. Chest 2017;152:1239–50.

# 18 Appendices

## 18.1 Appendix I: CTCAE Version 4.03

Toxicity will be scored using CTCAE Version 4.03 for toxicity and adverse event reporting. PDF of CTCAE v 4.03 can be downloaded without charge at

https://evs.nci.nih.gov/ftp1/CTCAE/CTCAE_4.03/CTCAE_4.03_2010-06-14_QuickReference_8.5x11.pdf

Article last updated on: June 16, 2010

All appropriate treatment areas have access to a copy of the CTCAE Version 4.03.

## 18.2 Appendix II: ECOG-Performance Status Scale

| Grade | ECOG |
|---|---|
| 0 | Fully active, able to carry on all pre-disease performance without restriction. |
| 1 | Restricted in physically strenuous activity but ambulatory and able to carry out work of a light or sedentary nature (e. g. light house work, office work). |
| 2 | Ambulatory and capable of all self-care but unable to carry out any work activities. Up and about more than 50 % of waking hours. |
| 3 | Capable of only limited self-care, confined to bed or chair more than 50 % of waking hours. |
| 4 | Completely disabled. Cannot carry on any self-care. Totally confined to bed or chair. |
| 5 | Dead. |

## 18.3 Appendix III: Revised RECIST response criteria (V1.1)

The following response criteria are based on the International RECIST Working Group Criteria for Measurement of Response/Treatment Effect in solid tumors by *Eisenhauer et al.* [39] and have been slightly modified.

### 18.3.1 Evaluation of target lesions

| | |
|---|---|
| * Complete Response (CR): | Disappearance of all target lesions |
| * Partial Response (PR): | At least a 30% decrease in the sum of the LD of target lesions, taking as reference the baseline sum LD |
| * Progressive Disease (PD): | At least a 20% increase in the sum of the LD of target lesions, taking as reference the smallest sum LD recorded since the treatment started or the appearance of one or more new lesions |
| * Stable Disease (SD): | Neither sufficient shrinkage to qualify for PR nor sufficient increase to qualify for PD, taking as reference the smallest sum LD since the treatment started |

### 18.3.2 Evaluation of non-target lesions

| | |
|---|---|
| * Complete Response (CR): | Disappearance of all non-target lesions and normalization of tumor marker level |
| * Incomplete Response/ Stable Disease (SD): | Persistence of one or more non-target lesion(s) or/and maintenance of tumor marker level above the normal limits |
| * Progressive Disease (PD): | Appearance of one or more new lesions and/or unequivocal progression of existing non-target lesions (1) |

Although a clear progression of “non target” lesions only is exceptional, in such circumstances, the opinion of the treating physician should prevail and the progression status should be confirmed later on by the review panel (or study chair).

### 18.3.3 Evaluation of best overall response

The best overall response is the best response recorded from the start of the treatment until disease progression/recurrence (taking as reference for PD the smallest measurements recorded since the treatment started). In general, the patient's best response assignment will depend on the achievement of both measurement and confirmation criteria

| Target lesions | Non-Target lesions | New Lesions | Overall response |
|---|---|---|---|
| CR | CR | No | CR |
| CR | Incomplete response/SD | No | PR |

| | | | |
|---|---|---|---|
| PR | Non-PD | No | PR |
| SD | Non-PD | No | SD |
| PD | Any | Yes or No | PD |
| Any | PD | Yes or No | PD |
| Any | Any | Yes | PD |

## 18.4 Appedix IV: Contraception Guidelines

### 18.4.1 Women of childbearing potential definitions and methods of contraception

#### 18.4.1.1 Defintions

**Woman of Childbearing Potential (WOCBP)**

A woman is considered fertile following menarche and until becoming post-menopausal unless permanently sterile. Permanent sterilization methods include hysterectomy, bilateral salpingectomy, and bilateral oophorectomy.

**Women in the following categories are not considered WOCBP**

- Premenarchal
- Premenopausal female with 1 of the following:
  - Documented hysterectomy
  - Documented bilateral salpingectomy
  - Documented bilateral oophorectomy

Note: Documentation can come from the site personnel's review of the participant's medical records, medical examination, or medical history interview.

- Postmenopausal female
  - A postmenopausal state is defined as 12 months of amenorrhea in a woman over age 45 years in the absence of other biological or physiological causes. In addition, females under the age of 55 years must have a serum follicle stimulating hormone, (FSH) level > 40 mIU/mL to confirm menopause.

### 18.4.2 Contraception guidance for female participants of child bearing potential

One of the highly effective methods of contraception listed below is required during study duration and until the end of relevant systemic exposure. WOCBP will be instructed to adhere to contraception during study treatment and for a period of 165 days (approximately 24 weeks) after the last dose of study drug. These durations have been calculated using the upper limit of the half-live estimated for relatlimab (27 days) and were based on the recommendations that WOCBP use

contraception for 5 half-lives plus 30 days (duration of ovulatory cycle). The contraception requirements for relatlimab apply for both treatment arms, nivolumab monotherapy and nivolumab/relatlimab combination therapy, despite the slightly shorter half-live of nivolumab (25 days).

| |
|---|
| **Highly Effective Contraceptive Methods That Are User Dependent**<br>*Failure rate of <1% per year when used consistently and correctly.*[a] |
| • Combined (estrogen- and progestogen-containing) hormonal contraception associated with inhibition of ovulation[b]<br>– oral<br>– intravaginal<br>– transdermal |
| • Progestogen-only hormonal contraception associated with inhibition of ovulation[b]<br>– oral<br>– injectable |
| **Highly Effective Methods That Are User Independent** |
| • Implantable progestogen-only hormonal contraception associated with inhibition of ovulation [b]<br>• Hormonal methods of contraception including oral contraceptive pills containing a combination of estrogen and progesterone, vaginal ring, injectables, implants and intrauterine hormone-releasing system (IUS)[c]<br>• Intrauterine device (IUD)[c]<br>• Bilateral tubal occlusion |
| • Vasectomized partner<br>*A vasectomized partner is a highly effective contraception method provided that the partner is the sole male sexual partner of the WOCBP and the absence of sperm has been confirmed. If not, an additional highly effective method of contraception should be used.* |
| • Sexual abstinence<br>*Sexual abstinence is considered a highly effective method only if defined as refraining from heterosexual intercourse during the entire period of risk associated with the study drug. The reliability of sexual abstinence needs to be evaluated in relation to the duration of the study and the preferred and usual lifestyle of the participant.*<br>• It is not necessary to use any other method of contraception when complete abstinence is elected.<br>• WOCBP participants who choose complete abstinence must continue to have pregnancy tests, as specified in Section 2.<br>• Acceptable alternate methods of highly effective contraception must be discussed in the event that the WOCBP participants chooses to forego complete abstinence |

| NOTES: |
|---|
| [a] Typical use failure rates may differ from those when used consistently and correctly. Use should be consistent with local regulations regarding the use of contraceptive methods for participants participating in clinical studies. |
| [b] Hormonal contraception may be susceptible to interaction with the study drug, which may reduce the efficacy of the contraceptive method. Hormonal contraception is permissible only when there is sufficient evidence that the IMP and other study medications will not alter hormonal exposures such that contraception would be ineffective or result in increased exposures that could be potentially hazardous. In this case, alternative methods of contraception should be utilized. |
| [c] Intrauterine devices and intrauterine hormone releasing systems are acceptable methods of contraception in the absence of definitive drug interaction studies when hormone exposures from intrauterine devices do not alter contraception effectiveness. |

| **Unacceptable Methods of Contraception*** |
|---|
| • Male or female condom with or without spermicide. Male and female condoms cannot be used simultaneously<br>• Diaphragm with spermicide<br>• Cervical cap with spermicide<br>• Vaginal Sponge with spermicide<br>• Progestogen-only oral hormonal contraception, where inhibition of ovulation is not the primary mechanism of action<br>• Periodic abstinence (calendar, symptothermal, post-ovulation methods)<br>• Withdrawal (coitus interruptus).<br>• Spermicide only<br>• Lactation amenorrhea method (LAM) |

*** Local laws and regulations may require use of alternative and/or additional contraception methods.**

### 18.4.3 Contraception guidance for male participants with partner(s) of child bearing potential

Male participants with female partners of childbearing potential are eligible to participate if they agree to the following during the treatment and until the end of relevant systemic exposure.

- Inform any and all partner(s) of their participation in a clinical drug study and the need to comply with contraception instructions as directed by the investigator.
- Male participants are required to use a latex or synthetic condom for any sexual activity with WOCBP condom for study duration and until end of relevant systemic exposure defined as 225 days (approximately 33 weeks) after the end of study treatment. These durations have

been calculated using the upper limit of the half-live for relatlimab (27 days) and are based on the recommendations for men who are sexually active with WOCBP use contraception for 5 half-lives plus 90 days after the last dose of relatlimab. The contraception requirements for relatlimab apply for both treatment arms, nivolumab monotherapy and nivolumab/relatlimab combination therapy, despite the slightly shorter half-live of Nivolumab (25 days).

- Female partners of males participating in the study to consider use of effective methods of contraception until the end of relevant systemic exposure, defined as 225 days (approximately 33 weeks) after the end of treatment in the male participant.
- Male participants with a pregnant or breastfeeding partner must agree to remain abstinent from penile vaginal intercourse or use a male latex or synthetic condom during each episode of penile penetration during the treatment and until 225 days (approximately 33 weeks) after the end of study treatment.
- Refrain from donating sperm for the duration of the study treatment and until 225 days (approximately 33 weeks) after the end of study treatment.

### 18.4.4 Collection of pregnancy information

Guidance for collection of Pregnancy Information and outcome of pregnancy on the Pregnancy Surveillance Form is provided in Section 10.

## 18.5 APPENDIX V: Medications Associated with QT Prolongation

The list below is not meant to be all inclusive. Please consult individual drug labels for further information.

| |
|---|
| quinidine, procainamide, disopyramide, |
| amiodarone, sotalol, ibutilide, dofetilide, |
| erythromycins, clarithromycin, |
| chlorpromazine, haloperidol, mesoridazine, thioridazine, pimozide, |
| cisapride, bepridil, droperidol, methadone, arsenic, chloroquine, domperidone, |
| halofantrine, levomethadyl, pentamidine, sparfloxacin, lidoflazine |

Further information concerning QT prolongation drug list can be found on:

https://crediblemeds.org
