# Supplementary material for: Neoadjuvant nivolumab with or without relatlimab in resectable non-small-cell lung cancer: a randomized phase 2 trial
Source: Nat Med. 2024 Apr 30;30(6):1602–11. doi: 10.1038/s41591-024-02965-0 (PMC11186754; doi:10.1038/s41591-024-02965-0)
Supplement: Supplementary file 2 — Reporting Summary [file 41591_2024_2965_MOESM2_ESM.pdf]

nature portfolio

Corresponding author(s): Martin Schuler

Last updated by author(s): Mar 21, 2024

Reporting Summary

Nature Portfolio wishes to improve the reproducibility of the work that we publish. This form provides structure for consistency and transparency in reporting. For further information on Nature Portfolio policies, see our Editorial Policies and the Editorial Policy Checklist.

Statistics

For all statistical analyses, confirm that the following items are present in the figure legend, table legend, main text, or Methods section.

n/a	Confirmed	
☐	☒	The exact sample size (*n*) for each experimental group/condition, given as a discrete number and unit of measurement
☐	☒	A statement on whether measurements were taken from distinct samples or whether the same sample was measured repeatedly
☐	☒	The statistical test(s) used AND whether they are one- or two-sided<br>*Only common tests should be described solely by name; describe more complex techniques in the Methods section.*
☐	☒	A description of all covariates tested
☐	☒	A description of any assumptions or corrections, such as tests of normality and adjustment for multiple comparisons
☐	☒	A full description of the statistical parameters including central tendency (e.g. means) or other basic estimates (e.g. regression coefficient) AND variation (e.g. standard deviation) or associated estimates of uncertainty (e.g. confidence intervals)
☐	☒	For null hypothesis testing, the test statistic (e.g. *F*, *t*, *r*) with confidence intervals, effect sizes, degrees of freedom and *P* value noted<br>*Give P values as exact values whenever suitable.*
☒	☐	For Bayesian analysis, information on the choice of priors and Markov chain Monte Carlo settings
☐	☒	For hierarchical and complex designs, identification of the appropriate level for tests and full reporting of outcomes
☒	☐	Estimates of effect sizes (e.g. Cohen's *d*, Pearson's *r*), indicating how they were calculated

Our web collection on statistics for biologists contains articles on many of the points above.

Software and code

Policy information about availability of computer code

Data collection	Clinical data was collected at the study sites using the respective hospital information system. Clinical data were captured in the clinical database using a proprietary electronic Case Report System provided by Alcedis GmbH (https://www.alcedis.de/en), which serves as subcontactor of the sponsor.
Data analysis	All descriptive statistical analyses of clinical study data were performed by Alecdis GmbH using SAS statistical software (version 9.4).<br><br>Gene expression analysis<br>Nanostring data was normalized and cleaned using NanoTube (version 1.6.0), entailing three steps. First, counts were scaled by comparing the geometric mean of positive control features between samples. Secondly, genes where at least 50% of samples are less than 2 standard deviations above the mean of negative controls were removed. Thirdly, counts were scaled by comparing the geometric mean of housekeeping genes between samples. Afterwards, differential expression analysis was performed using the quasi-likelihood F-test approach of EdgeR (two-sided, version 3.40.0). First, genes differentially expressed between sample types (resected tumor vs. biopsy) were determined, while correcting for additive batch effects induced by pathological response (MPR=1/0) and tumor classification (LUAD, LUSC, LCNEC, sarcomatoid). Secondly, genes differentially expressed between MPR and no MPR were determined separately within each sample type and study arm. Reproducibility was ensured by implementing above analysis as a Snakemake workflow.<br><br>Genomic variant calling<br>Demultiplexing of sequenced reads was achieved using bcl2fastq (version 2.2). Further data analysis was performed using our open-source Snakemake workflow dna-seq-varlociraptor (version 3.24, https://github.com/snakemake-workflows/dna-seq-varlociraptor), entailing the following steps. Adapter trimming was performed using Cutadapt (version 4.1, https://doi.org/10.14806/ej.17.1.200). Quality was monitored using MultiQC (version 1.14) including FASTQC (version 0.11.9, https://www.bioinformatics.babraham.ac.uk/projects/fastqc/), Somalier

(version 0.2.1846), and samtools (version 1.1447). Reads were mapped to GRCh38 using bwa-mem (version 0.7.17, https://doi.org/10.48550/arXiv.1303.3997) and deduplicated using Picard-Tools (version 2.26). Base qualities were recalibrated using GATK (version 4.2). Single nucleotide variants (SNV) and small indels were detected using Freebayes (version 1.3.6, https://doi.org/10.48550/arXiv.1207.3907) and classified into events of interest (somatic in biopsy or resection, germline) using Varlociraptor (version 8.3). Variant calls were distinguished from noise by controlling the (Bayesian) local false discovery rate using Varlociraptor. Variant annotation (with impact, prior knowledge) was performed using VEP (version 109.3). Extraction of variants of interest was performed using vembrane (version 1.0). Specifically, for Figure 2 a, variants were filtered to be non-synonymous, having a REVEL score > 0.5 if available (i.e. being predicted as pathogenic), having a gnomad allele frequency < 0.2, being not marked as benign or likely benign in ClinVar and impacting one of the TCGA LUAD 500 cancer genes. Missing WES data was complemented with results from panel sequencing (TSO500) whenever available. To identify genes that had altered variant allele frequencies (VAFs) comparing the diagnostic biopsy and the resected tumor, genes defined by oncobk (https://www.oncokb.org/cancer-genes) were inspected. To adjust for the different tumor cell content between biopsies and resected tumors, probabilities were calculated that the variants were not present in the normal sample of the same patient and that the VAF had changed prior to surgery. Only variants that were not marked by ClinVar as benign or likely benign and had a REVEL score > 0.7 are reported in Supplementary Figure 3.

Inference of subclonal diversity
Tumor purity estimation
Prior estimates p1 and p2 of tumor purity of samples from resected tumors were obtained by two independent pathologists evaluating sections stained with hematoxylin and eosin (H&E). For the other samples, a posterior estimate of the tumor purity of each sample was obtained as follows: We plotted the somatic variant allele frequency (VAF) distribution of the pretherapeutic biopsy and the resected tumor samples of each patient. For this, the maximum a posteriori allele frequency estimates provided by Varlociraptor without adjusting for purity were used (i.e. no sample contamination assigned, see https://varlociraptor.github.io/docs/calling). The expectation is that without copy number variants any somatic variant may at most have a VAF equal to the tumor purity. Read sampling variance and copy number variation can generate peaks beyond the tumor purity. For resection samples, we proceeded as follows: Let v be the highest VAF of the distribution or a threshold for which higher VAFs could as well be explained by sampling or copy number variation. If v was consistent with the prior estimates (i.e. within the interval [p1,p2]) and the prior estimates were agreeing to a sufficient degree (p2-p1 ≤ 0.2) we reported v as the posterior purity. Otherwise, we considered the posterior purity as unknown (28/56 cases). For samples where the resected tumor had a posterior purity, we compared the distribution of the pretherapeutic biopsy and the resected tumor, and inferred a posterior estimate by scaling the biopsy distribution to match the shape of the resection distribution. Such scaling was possible in all investigated cases.

Subclonal diversity
For patients with posterior purity estimates, subclonal diversity was visualized in the following way: During tumor evolution, each somatic mutation that does not lead to cell death can be seen as an event generating a new subclone. We made the simplifying assumption that each non-lethal somatic mutation during development of the tumor generates one new subclone. Thus, the number of somatic variants can be seen as a proxy for the number of subclones, and each somatic variant can be considered as a representative of the subclone that originates in it. Note that this neglects the fact that multiple somatic variants can occur during one cell division. However, under the assumption that all considered samples have a similar somatic mutation rate, the subclone counts obtained would still be proportional to the true number of subclones, and thereby comparable across patients.
Thus, for each patient, we obtained the sufficiently relevant subclones by considering variants with posterior probability ≥ 0.95 according to Varlociraptor for being somatic in the pretreatment biopsy or in the resected tumor, and purity adjusted variant allele frequency ≥ 0.1. For being able to be certain that a variant is detectable in both, the pretreatment biopsy and the resected tumor, we further filtered them such that they would be in expectation represented by at least 2 reads if occurring at the same frequency in the respective other sample (pretreatment biopsy for resected tumor; resected tumor for pretreatment biopsy). Patients where both, pretreatment biopsy and resected tumor, had no such somatic variants/subclones after filtering were omitted as they would not allow any statement about subclonal gains and losses. Then, variants with VAF = 0.0 in the resected tumor but VAF ≥ 0.1 in the pretreatment biopsy were counted as "lost subclones" following study therapy. Variants with VAF = 0.0 in the pretreatment biopsy but VAF ≥ 0.1 in the resected tumor were counted as "gained subclones" following study therapy. Note that since the pretreatment biopsy may not represent the entire primary tumor, a "gain" is not distinguishable from an enrichment of a variant that was spatially missed in the biopsy.

References:
Class, C.A., Lukan, C.J., Bristow, C.A. & Do, K.A. Easy NanoString nCounter data analysis with the NanoTube. Bioinformatics 39, btac762 (2023).
Robinson, M.D., McCarthy, D.J. & Smyth, G.K. edgeR: a Bioconductor package for differential expression analysis of digital gene expression data. Bioinformatics 26, 139-140 (2010).
Mölder, F., et al. Sustainable data analysis with Snakemake. F1000Res 10, 33 (2021).
Ewels, P., Magnusson, M., Lundin, S. & Käller, M. MultiQC: summarize analysis results for multiple tools and samples in a single report. Bioinformatics 32, 3047-3048 (2016).
Pedersen, B.S., et al. Somalier: rapid relatedness estimation for cancer and germline studies using efficient genome sketches. Genome Med 12, 62 (2020).
Danecek, P., et al. Twelve years of SAMtools and BCFtools. Gigascience 10, giab008 (2021).
DePristo, M.A., et al. A framework for variation discovery and genotyping using next-generation DNA sequencing data. Nat Genet 43, 491-498 (2011).
Köster, J., Dijkstra, L.J., Marschall, T. & Schönhuth, A. Varlociraptor: enhancing sensitivity and controlling false discovery rate in somatic indel discovery. Genome Biol 21, 98 (2020).
McLaren, W., et al. The Ensembl Variant Effect Predictor. Genome Biol 17, 122 (2016).
Hartmann, T., Schröder, C., Kuthe, E., Lähnemann, D. & Köster, J. Insane in the vembrane: filtering and transforming VCF/BCF files. Bioinformatics 39, btac810 (2023).

All code developed and used in this study is open source. The Snakemake workflows for whole exome sequencing analysis and NanoString nCounter gene expression analysis can be found under the DOIs 10.5281/zenodo.10838511 and 10.5281/zenodo.10838908.

For manuscripts utilizing custom algorithms or software that are central to the research but not yet described in published literature, software must be made available to editors and reviewers. We strongly encourage code deposition in a community repository (e.g. GitHub). See the Nature Portfolio guidelines for submitting code & software for further information.

Data

Policy information about availability of data

All manuscripts must include a data availability statement. This statement should provide the following information, where applicable:

- Accession codes, unique identifiers, or web links for publicly available datasets
- A description of any restrictions on data availability
- For clinical datasets or third party data, please ensure that the statement adheres to our policy

The study protocol is provided with the supplemental materials. Once the study is formally completed, a Clinical Study Report with tabulated data listings is prepared, which will be considered for sharing upon request from qualified scientists, if there is legal authority to share the data and there is no likelihood of participant re-identification. De-identified raw data from gene expression profiling and whole exome sequencing have been deposited in the European Genome-Phenome Archive (EGA) with accession number EGAS00001007753. Requests should be submitted to the Office of Data Governance of the study sponsor, University Hospital Essen (https://www.uk-essen.de/), which also serves as Data Access Committee (DAC). Responses can be expected within 4 weeks.

Research involving human participants, their data, or biological material

Policy information about studies with human participants or human data. See also policy information about sex, gender (identity/presentation), and sexual orientation and race, ethnicity and racism.

Reporting on sex and gender	To describe the patient cohort, sex and gender is reported using the declaration of each study subject. This represent the sex and gender the respective study subject identifies herself or himself with.
Reporting on race, ethnicity, or other socially relevant groupings	Not applicable.
Population characteristics	The patient population is described in the manuscript and in the study protocol, which is provided with the supplemental material. In brief, adult patients (age above 18 years) with histologically or cytologically confirmed non-small cell lung cancer (NSCLC) eligible for anatomic resection, with the following specifications: Clinical stages I A3, I B, II and selected stage III A (T3 N1, T4 with satellite nodule in the same lung N0/N1, selected T1a-T2b N2 cases considered suitable for primary surgical approach by the multidisciplinary tumor board) according to UICC 8th edition.
Recruitment	Study patients were recruited from the patient populations of the study sites, which reflect the full spectrum of the populations of the three cities and regions. Patients potentially eligible according to the study inclusion and exclusion criteria were offered trial participation by the principal investigators or their delegates at the three enrolling sites. No additional measures were in place to exclude selection bias.
Ethics oversight	The study was approved by the responsible ethics committees and competnent regulatory authorities at each participating study site and country. In the legislature of the study sponsor and study site Essen the Ethics Committee of the Medical Faculty of the University Duisburg-Essen, Essen, Germany, granted primary approval on September 10, 2019 (19-8828-AF). The competent regulatory authority in the legislature of the study sponsor and study site Essen, the Paul-Ehrlich-Institut (Federal Institute for Vaccines and Biomedicines), Langen, Germany, granted primary approval on November 27, 2019 (EudraCT-Nr. 2109-007278-29, Vorlage-Nr. 3834/01). For study site Hasselt, approval was granted by the Ethics Committee OLV Ziekenhuis VZW, Aalst, Belgium (EudraCT-Nr. 2109-007278-29 Pilot 262-SM001, Reference 202/082), and the Federal Agency for Medicines and Health Products, Brussels, Belgium (EudraCT-Nr. 2109-007278-29 Pilot 262, 1240640 M). For study site Amsterdam, approval was granted by the METC - The Netherlands Cancer Institute, Antoni van Leeuwenhoek (NKI-AVL), Amsterdam, The Netherlands (NL72532.031.20), and by the Centrale Commissee Mensgebonden Onderzoek, The Hague, The Netherlands (Decree NL72532.031.21 CA).

Note that full information on the approval of the study protocol must also be provided in the manuscript.

Field-specific reporting

Please select the one below that is the best fit for your research. If you are not sure, read the appropriate sections before making your selection.

☒ Life sciences ☐ Behavioural & social sciences ☐ Ecological, evolutionary & environmental sciences

For a reference copy of the document with all sections, see nature.com/documents/nr-reporting-summary-flat.pdf

Life sciences study design

All studies must disclose on these points even when the disclosure is negative.

Sample size	Based on published results of a study with preoperative nivolumab each study arm included up to 30 evaluable patients with the expectation that at least 26 of 30 patients treated in each study arm will undergo curatively intended surgery within 6 weeks of initiation of study treatment. At maximum 4 of 30 patients may experience a delay of curatively intended surgery beyond day 43 (with study treatment being administered on day 1), either due to toxicities or disease progression, to declare the study arm feasible. Continuous monitoring of prespecified stopping boundaries was applied to facilitate early termination of non-feasible study arms to reduce patient risks. Fruther details can be reviewed in the clinical study protocol (Supplementary information).

Reference:
Forde, P.M., et al. Neoadjuvant PD-1 Blockade in Resectable Lung Cancer. N Engl J Med 378, 1976-1986 (2018).

Data exclusions

No data were excluded from this report. One patient could not be analyzed for secondary and exploratory endpoints as curatively intended resection was not performed due to intraoperative detection of pleural carcinosis. Details are presented in the article.

Replication

Per protocol this study prospectively enrolls up to 30 patients per treatment arm. This may be viewed as "30 replicates" of the respective study intervention.

Randomization

Randomization was performed by by Alcedis GmbH (https://www.alcedis.de/en), which serves as subcontactor of the sponsr, using a computer system. No stratification was applied.

Blinding

As this is a non-comparative study, blinding is not required.

Reporting for specific materials, systems and methods

We require information from authors about some types of materials, experimental systems and methods used in many studies. Here, indicate whether each material, system or method listed is relevant to your study. If you are not sure if a list item applies to your research, read the appropriate section before selecting a response.

Materials & experimental systems

n/a	Involved in the study
☐	☒ Antibodies
☒	☐ Eukaryotic cell lines
☒	☐ Palaeontology and archaeology
☒	☐ Animals and other organisms
☐	☒ Clinical data
☒	☐ Dual use research of concern
☒	☐ Plants

Methods

n/a	Involved in the study
☒	☐ ChIP-seq
☐	☒ Flow cytometry
☒	☐ MRI-based neuroimaging

Antibodies

Antibodies used

Therapeutic antibodies:
The investigational medical products, nivolumab and relatlimab, were provided by the manufacturer, Bristol Myers Squibb. During the conduct of the study, nivolumab was globally approved for patient treatment in several cancer entities including non-small-cell lung cancer. Relatlimab was still an investigational agent, but has since been approved for the treatment of patients with melanoma. All relevant information was provided by the investigator brochures of nivolumab and relatlimab, which were regularly updated by the manufacturer, and approved by the respective regulatory authorities.

Diagnostic antibodies:
PD-L1: supplier name: Dako, catalog number: M3653, clone name: 22C3 , lot number: 11221493, platform: Ventana Benchmark Ultra, antigen retrieval: boiling in CC1 48 min, incubation with primary antibody: 1:40 for 60 min, Optiview detection system

CD8: supplier name: Dako, catalog number: M7103, clone name: C8/144B, lot number: 20055137, platform: Ventana Benchmark Ultra, antigen retrieval: boiling in CC1 40 min, incubation with primary antibody: 1:150 for 24 min, Optiview detection system

Antibody panel for detecting CD8 T cells in peripheral blood:

Antibody	Fluorochrome	Clone	Isotype	Dilution	Source	Catalog#
CD3	ECD	UCHT1	Mouse IgG1, k	1:50	Beckman-Coulter	A07748
CD4	AF700	OKT4	Mouse IgG2b, k	1:100	BioLegend	317425
CD8	APC/Cy7	SK1	Mouse IgG1, k	1:100	BioLegend	344713
GrzB	BV421	QA18A28	Rat IgG1, k	1:200	BioLegend	396413

Antibody panel for myeloid immune cell populations in tumor tissue cell suspensions:

Antibody	Fluorochrome	Clone	Isotype	Dilution	Source	Catalog#	LOT #
CD11c	BV650	3.9	Mouse IgG1, k	1:100	Biolegend	301637	B329910
HLA-DR	BV421	L243	Mouse IgG2, k	1:100	Biolegend	307635	B360315
CD4	Per CP/Cy5.5	RPA-T4	Mouse IgG1, k	1:200	Biolegend	300529	B313462
CD3	AF700	UCHT1	Mouse IgG1, k	1:200	Biolegend	300424	B363398
CD8	BV510	SK1	Mouse IgG1, k	1:200	Biolegend	344731	B293257
CD66b	PE	6/40C	Mouse IgG1, k	1:100	Biolegend	392903	B340558
CD19	PE/Cy 7	HIB19	Mouse IgG1, k	1:100	Biolegend	302216	B368441
CD24	APC	ML5	Mouse IgG2a, k	1:100	Biolegend	311117	B333887
CD206	BV605	15-2	Mouse IgG1, k	1:100	Biolegend	321119	B342527
CD123	PE/Cy5	6H6	Mouse IgG1, k	1:200	Biolegend	306008	B281793

CD56	PE/Dazzle594	HCD56	Mouse IgG1, k	1:200	Biolegend	318347	B315298
CD16	APC/Fire750	3G8	Mouse IgG1, k	1:200	Biolegend	302059	B370797
CD14	BV785	M5E2	Mouse IgG2, k	1:200	Biolegend	301839	B360456
CD45	AF488	2D1	Mouse IgG1, k	1:250	Biolegend	368536	B324537

Antibody panel for T-cell immune cell populations in tumor tissue cell suspensions:

Antibody	Fluorochrome	Clone	Isotype	Dilution	Source	Catalog#	LOT #
CD3	AF700	SK7	Mouse IgG1, k	1:100	Biolegend	300424	B363398
CD4	PerCP/Cy 5.5	RPA-T4	Mouse IgG1, k	1:100	Biolegend	300529	B313462
CD196	BV650	G034E3	Mouse IgG2b, κ	1:100	Biolegend	353426	B318067
CD39	BV605	A1	Mouse IgG1, k	1:100	Biolegend	328236	B339983
CD25	BV421	BC96	Mouse IgG1, k	1:100	Biolegend	302630	B365978
CD127	APC	A019D5	Mouse IgG1, k	1:100	Biolegend	351316	B366604
CD8	BV510	SK1	Mouse IgG1, k	1:100	Biolegend	344732	B362160
CD183	BV785	G825H7	Mouse IgG1, k	1:100	Biolegend	353737	B361913
CD194	PE/Dazzle594	L291H4	Mouse IgG1, k	1:100	Biolegend	359420	B359566
CD45	AF488	2D1	Mouse IgG1, k	1:200	Biolegend	368535	B353778
CD19	PE/Cy 7	HIB19	Mouse IgG1, k	1:100	Biolegend	302216	B368441

Validation

Therapeutic antibodies:
All relevant information for nivolumab and relatlimab can be obtained in the Summary of Product Characteristics (SmpC) provided by the manufacturer, Bristol Myers Squibb. In addition, investigator brochures (IB) of nivolumab and relatlimab were provided to the investigators, which were regularly updated by the manufacturer, and approved by the respective regulatory authorities.

Diagnostic antibodies:
All diagnostic antibodies were commercially available and were applied according to the manufacturers' instructions as detailed above. Validation was performed per DIN EN ISO/IEC 17020 / ISO 15189 criteria. On-slide positive controls were used throughout on every slide.

Clinical data

Policy information about clinical studies
All manuscripts should comply with the ICMJE guidelines for publication of clinical research and a completed CONSORT checklist must be included with all submissions.

Clinical trial registration

NCT04205552

Study protocol

The clinical study protocol is provided with the supplementary materials.

Data collection

Patients were enrolled at the lung cancer centers of three study sites, Essen (Germany), Hasselt (Belgium) and Amsterdam (The Netherlands) between March 4, 2020 and July 15, 2022. Data were collected from the hospital documentation and information systems of the study sites by the principal investigators and their delegates, including study physicians and trained and certified study personal of the clinical trial centers. Data were entered into the study data base using electronic case report forms as described above. Source data were verified at the study sites by monitors, who are trained and certified personnel of the sponsor CRO (University Medicine Essen Study Center GmbH) or its subcontractors.

Outcomes

All primary and secondary study endpoints were defined according to the research aims of the study. They were prespecified in the clinical study protocol.

The primary study endpoint is the number of patients undergoing curatively intended surgery of non-small cell lung cancer within 43 days of initiation of study therapy.

Secondary endpoints include:
• Objective response rate (RECIST 1.1) prior to surgery
• Pathological response rate (complete pathological responses defined as absence of viable tumor cells on routine hematoxylin and eosin staining of resected tumors and lymph nodes; rate of major pathological responses defined as 10% or less viable tumor cells on routine hematoxylin and eosin staining of resected tumors)
• R0 resection rate
• Disease-free survival rate at 12 months per RECIST 1.1
• Overall survival rate at 12 months
• Safety and tolerability of preoperative immunotherapy
• Morbidity and mortality within 90 days of curative surgery

The primary endpoint was continuously monitored by the study statistician. At maximum 4 of 30 patients may experience a delay of curatively intended surgery beyond day 43 (with study treatment being administered on day 1), either due to toxicities or disease progression, to declare the study arm feasible. Continuous monitoring of prespecified stopping boundaries was applied to facilitate early termination of non-feasible study arms to reduce patient risks.

All secondary parameters were evaluated in an explorative or descriptive manner. Radiographic and nuclear imaging assessments at base line were conducted within standard of care at the study sites. Specifically, all 60 patients underwent whole body imaging by FDG-PET/CT. For exclusion of brain metastases, 41 patients underwent contrast-enhanced brain MRI scanning, 18 patients

underwent contrast-enhanced brain CT scanning (due to contraindications or intolerance of MRI imaging, or unavailability of an MRI slot within the protocol-defined screening period). In one patient with stage I B NSCLC no brain imaging was performed per Dutch guidelines. All patients underwent CT or PET/CT imaging immediately prior to surgery. Radiographic response was evaluated at the study sites following RECIST version 1.1. For exploratory analyses, nuclear imaging data were acquired prior to surgery. Histology and biomarker studies were conducted within standard of care at the study sites. PD-L1 expression by tumor cells was assessed locally using the primary antibody clone 22C3 (DAKO/Agilent M3653) following validated protocols with continuous external quality assurance (QUIP, UK NEQAS, NordiQC).

Exploratory endpoints are assessed in tumor and lymph node samples, blood cells, plasma and serum.

Plants

Seed stocks

Not applicable.

Novel plant genotypes

Not applicable.

Authentication

Not applicable.

Flow Cytometry

Plots

Confirm that:

☒ The axis labels state the marker and fluorochrome used (e.g. CD4-FITC).

☒ The axis scales are clearly visible. Include numbers along axes only for bottom left plot of group (a 'group' is an analysis of identical markers).

☒ All plots are contour plots with outliers or pseudocolor plots.

☒ A numerical value for number of cells or percentage (with statistics) is provided.

Methodology

Sample preparation

Peripheral blood immune cells:
cryo-preserved peripheral blood mononuclear cells were thawed and rested overnight in RPMI medium supplemented with 10% fetal calf serum (FCS), 100 U/ml penicillin and 100 µg/ml streptomycin (PAA Laboratories) at 37°C in a 5% CO2 atmosphere. Antibody staining of cell surface molecules (30min, 4°C) was followed by fixation and permeabilization for staining of intracellular markers (30 min, 4 °C).

Single cell suspensions from resected tumors:
Tumor tissue was put in 1 ml of digestion medium (DMEM/F12/HEPES solution supplemented with penicillin/streptomycin and 1% bovine serum albumin and containing collagenase, hyaluronidase and DNAse I) and cut into small pieces. In order to facilitate dissociation the tissue was incubated for 40 minutes at 37 °C and pipetted every 10 minutes during the incubation period. The resulting cell suspension was transferred to a 50 ml centrifuge tube and centrifuged at 300×g for 10 minutes at ambient temperature. The pellet was resuspended in trypsin/EDTA and incubated for 5 minutes at ambient temperature. After inactivation of the trypsin by DMEM/F12/HEPES solution containing 10% FCS, the cell suspension was again triturated and filtered through a 40 µm cell strainer. After washing the filter with 50 ml PBS the cells were centrifuged at 400×g for 5 minutes at ambient temperature. Following one more washing step with phosphate-buffered saline, cell number and viability was measured using the NucleoCounter NC-3000 and one to two million cells per vial were cryopreserved in FCS-containing 10% DMSO.

Instrument

Peripheral blood immune cells:
Gallios flow cytometer (Beckman Coulter, Krefeld, Germany)

Single cell suspensions from resected tumors:
CytoFLEX LX (Beckman Coulter, Krefeld, Germany)

Software

Peripheral blood immune cells:
Kaluza software (Beckman Coulter), CytExpert V2.3 software (Beckman)

Single cell suspensions from resected tumors:
CytExpert V2.3 (Beckman Coulter, Krefeld, Germany) and FlowJo Software V10 (Tree Star, Ashland, USA)

Cell population abundance

Peripheral blood immune cells:
Samples containing 200,000 cells were stained with antibody panels for surface and intracellular markers. The minimum

abundance of CD8+ T cell subsets presented in the report was above 300 cells.

Single cell suspensions from resected tumors:
Two aliquots, each containing 500,000 cells, were stained with one of the two antibody panels for surface markers. The abundance of the specific cell populations presented in the report ranged from 6 to several hundred cells. Of note, in one patient no neutrophil granulocytes were identified in the sample.

Gating strategy	The gating strategies are graphically represented in Supplementary Figure 2.

☒ Tick this box to confirm that a figure exemplifying the gating strategy is provided in the Supplementary Information.
